# Amyloid β oligomers inhibit growth of human cancer cells

**DOI:** 10.1371/journal.pone.0221563

**Published:** 2019-09-11

**Authors:** Bozena Pavliukeviciene, Aiste Zentelyte, Marija Jankunec, Giedre Valiuliene, Martynas Talaikis, Ruta Navakauskiene, Gediminas Niaura, Gintaras Valincius

**Affiliations:** 1 Department of Bioelectrochemistry and Biospectroscopy, Institute of Biochemistry, Life Sciences Center, Vilnius University, Vilnius, Lithuania; 2 Department of Molecular Cell Biology, Institute of Biochemistry, Life Sciences Center, Vilnius University, Vilnius, Lithuania; University of South Alabama Mitchell Cancer Institute, UNITED STATES

## Abstract

Effects of amyloid beta (Aβ) oligomers on viability and function of cell lines such as NB4 (human acute promyelocytic leukemia), A549 (human lung cancer (adenocarcinomic alveolar basal epithelial tumor)) and MCF-7 (human breast cancer (invasive breast ductal carcinoma)) were investigated. Two types of Aβ oligomers were used in the study. The first type was produced in the presence of oligomerization inhibitor, hexafluoroisopropanol (HFIP). The second type of amyloids was assembled in the absence of the inhibitor. The first type preparation was predominantly populated with dimers and trimers, while the second type contained mostly pentadecamers. These amyloid species exhibited different secondary protein structure with considerable amount of antiparallel β sheet structural elements in HFIP oligomerized Aβ mixtures. The effect of the cell growth inhibition, which was stronger in the case of HFIP Aβ oligomers, was observed for all cell lines. Tests aiming at elucidating the effects of the amyloid species on cell cycles showed little differences between amyloid preparations. This prompts us to conclude that the effect on the cancer cell proliferation rate is less specific to the biological processes developing inside the cells during the proliferation. Therefore, cell growth inhibition may involve interactions with the peripheral parts of the cancer cells, such as a phospholipid membrane, and only in case of the NB4 cells, where accumulation of amyloid species inside the cells was detected, one may imply the opposite. In general, cancer cells were much less susceptible to the damaging effects of amyloid oligomers compared to earlier observations in mixed neuronal cell cultures.

## Introduction

Short, 4.5 kDa amyloid-β (Aβ) peptide is produced in the brain. It is a by-product of the biochemical processing of the abundant amyloid precursor protein (APP), which functions in the brain. Despite its involvement in numerous biological processes, its function and biochemical processing are not fully understood [1, 2]. The normal processing of APP is carried out by the α-secretase which releases a large soluble extracellular domain sAPP-α which possibly undergoes further degradation by extracellular proteases [3]. Abnormal processing of β- and γ-secretases yields typically 39–43 residue peptides, of which Aβ(1–40) and Aβ(1–42) were mostly studied in the context of the Alzheimer’s disease etiology. Therefore modulation of the activity of β- and γ-secretases is seen as an early preventive measure in Alzheimer’s disease [4].

Recent scientific data indicates that Alzheimer’s patients have a lower incidence of lung cancer, however they are more susceptible to developing glioblastoma [5]. Although neuroblastoma and glioma cells are one of the most frequently studied model cancer systems of *in vitro* toxicity of Aβ species [6–8], mechanistic insights of interrelationship between Alzheimer‘s and other types of cancer are still scarce. To this date Aβ(1–42) was found to trigger autophagic cell death in both human glioma and human neuroblastoma cell lines [9]. Brain cancer cells were used in assessing protective properties of various compounds such as melatonin against the toxic effects of Aβ species [10]. Numerous works demonstrated cell specificity of Aβ induced biological events. For example human neuroblastoma but not human embryonic kidney cells were capable of taking up presumably monomeric (not reported what oligomer form was used) Aβ at nanomolar concentration range. This raises the question if other than brain tumor cells are affected by the Aβ species? Simultaneous development of neurodegenerative processes and cancer is quite possible at older age so the answer to this question may be important from the standpoint of understanding complex processes leading to a multimorbidity.

It is known that Aβ peptide exhibits strong propensity for oligomerization, which leads to formation of insoluble fibrils. The oligomerization is a complex process involving parallel/series stages resulting in polymorphous mixtures of amyloid species including globular and/or annular oligomers, protofibrils and fibrils [11–13].

Amyloid species as shown in numerous studies affect physiology and function of the brain in animal models [14]. A particular effect on brain is strongly dependent on the degree of oligomerization as well as morphology. For example, Aβ*56 oligomers (56 kDa molecular mass species) impair memory without neuronal loss in middle-aged mice [14]. Soluble Aβ oligomers exhibit strong synaptotoxic effect [15], while insoluble Aβ fibrils promote microglia activity and trigger inflammation in the brain [6, 16]. Injection of synthetic Aβ oligomer preparations into mice accelerates *tau* hyperphosphorylation and leads to tangle formation reminiscent of the Alzheimers disease hallmark found in *post mortem* brains [17]. It can be expected though that in living brain various Aβ species are present simultaneously. Because precise quantification of various oligomer forms in organism is currently unavailable, the exact molecular map of the disease involving Aβ species is unknown.

*In vitro* toxicity tests allow in principle controlling compositions of Aβ oligomers in experiments. Experiments using primary cell lines show strong dependence of biological effects on the type of the oligomers [7, 18]. Soluble oligomers at concentrations exceeding physiological levels trigger oxidative stress [6] and apoptosis [19]. Small 2−5 nm, well characterized preparations of soluble oligomers induce death of neurons by necrosis [20]. Size-dependent nature of the cytotoxicity of Aβ oligomers was demonstrated [20]. Large Aβ oligomers [20] or a mixture of oligomeric Aβ species triggered autophagy processes in mixed neuronal-glial cultures from rat *cerebellum* [21]. The interactions of cell membranes with amyloid oligomers are considered as one of the central events in Alzheimers etiology. Such interaction leads to lysis, impairment of the homeostasis and cell death [22–24]. Despite evidence of the toxicity of Aβ to cell cultures, large amount of experimental studies were performed without reporting protocols to control oligomeric compositions of Aβ (see e.g. [6]). Therefore our approach was to investigate the effect of Aβ oligomers on human cancer cells using characterized amyloid β preparations.

## Materials and methods

### Preparation of Aβ(1–42) oligomers

#### HFIP protocol (protocol-I)

0.4 mg Aβ(1–42) were dissolved in 160 μl HFIP. The solution was kept in an open vial until all HFIP was fully evaporated. Aβ(1–42) residues were dissolved in 33 μL of 0.1 M NaOH at pH 12 and incubated for 15 min followed by the addition of 800 μL of PBS buffer (0.1 M NaCl, 0.01 M NaH_2_PO_4_, pH 7.4) and were kept undisturbed at room temperature for 24 hours. Until further use, samples were stored at 4°C.

#### HFIP—free protocol (protocol-II)

0.4 mg Aβ(1–42) were directly dissolved in 33 μL of 0.1 M NaOH at pH 12 and incubated for 15 min, followed by the addition of 800 μL of PBS buffers (0.1 M NaCl, 0.01 M NaH_2_PO_4_, pH 7.4) and were kept undisturbed at room temperature for 24 hours. Samples were stored at 4°C until further use.

#### HFIP—free protocol with Ab(1–42) FAM-labeled

0.4 mg Aβ(1–42) and 2.6 μg Aβ(1–42) FAM-labeled were directly dissolved in 41.8 μL of 0.1 M NaOH at pH 12 and incubated for 15 min, followed by the addition of 800 μL of PBS buffers (0.1 M NaCl, 0.01 M NaH_2_PO_4_, pH 7.4) and were kept undisturbed at room temperature for 24 hours. Samples were stored at 4°C until further use.

In all experiments the synthetic Aβ(1–42) and Aβ(1–42) FAM-labeled peptide was purchased from the American Peptide Company (USA).

The amyloid oligomer solutions were filtered through the Millipore 0.22 μm filter before biological assays or biophysical tests were performed. All data presented in the current work refers to 0.22 μm filtered preparations unless stated otherwise. Upon request by one of the reviewers and to ensure the fibril amyloid components are not present in preparations additional series of experiments were carried out with the centrifuge-filtered samples using Amicon centrifugal filters with the molecular weight cut-off (MWCO) at 100 kDa. The experiments using centrifuge filtered amyloid samples were performed using amyloid peptides purchased from GenScript (Hong Kong) We found the data obtained on filter-centrifuged samples qualitatively replicates data on samples filtered only by 0.22 μm filters.

### High performance liquid chromatography

High performance liquid chromatography (HPLC) analysis was performed on a Perkin Elmer system that consists of a microprocessor controlled Perkin Elmer model 200 eluent delivery pump and a fixed wavelength Perkin Elmer model 200 UV-VIS spectrophotometer detection system. Samples were injected via a Perkin Elmer model 200 autosampler injector valve fitted with a 15 μl volume injector loop. The sample concentration was 10 μM. Separation was performed on a 150 mm long x 4.6 mm inner diameter column (Bio SEC-3, Agilent) with 3 μm silica absorbent. Mobile phase was isocratic 0.1 M NaCl, 0.01 M NaH_2_PO_4_ buffer at pH 7.4, flow rate of 0.25 ml/min, at a pressure of 34 bar (or 496 psi). Column temperature was maintained constant at 30 ^o^C. All other parts of the system were maintained at room temperature (22 ± 2 ^o^C). Data collection and handling were carried out by the manufacturer (Perkin Elmer) provided software. Peptide UV spectrophotometric detection was carried out at 275 nm wavelength. Seven protein standards were used to construct the calibration curve relating the retention time and the molecular weight of amyloid species (see supporting information S1 Appendix).

### Atomic force microscopy

The physical properties and morphology of the prepared of Aβ(1–42) oligomers were observed by atomic force microscopy (Dimension Icon AFM system; Bruker, USA), operated in tapping mode in air. Model ScanAsyst Air or FESP (Bruker, USA) microcantilevers were used in this work. About 30 μl of a 10 μM Aβ(1–42) solution (HFIP or HFIP-free protocols) was spotted on freshly cleaved mica (V-4 grade, SPI Supplies, USA), incubated at room temperature for 10 min and rinsed with deionized water (Millipore Inc., USA), then blown dry with a nitrogen stream. Typically, the surface was scanned with scan size 1 μm x 1 μm, 1024 samples/line and images were acquired at 0.4 Hz scan rate. After scanning, the raw images were processed with Nanoscope v1.41 (Bruker, USA) image processing software. Images were flattened by 3^rd^ order polynomial fit and statistically evaluated with particle analysis feature. Several images were used for each sample to determine the average height and height distribution.

### Fourier transform infrared spectroscopy (FTIR)

Infrared spectra were recorded on FTIR spectrometer Vertex 80v (Bruker, Germany) equipped with the liquid nitrogen cooled MCT narrow band detector. The spectral resolution was set at 2 cm^-1^. Spectra were acquired by co-adding 400 scans. The sample chamber and the spectrometer were evacuated during the measurements. FTIR spectra were recorded in transmission mode. Samples were deposited at CaF_2_ substrate from 100 μM solution and dried in air; blank CaF_2_ substrate was used as a reference. Parameters of the bands were determined by fitting the experimental contour with Gaussian-Lorentzian form components using the GRAMS/AI 8.0 (Thermo Scientific, USA) software.

### Cell lines, culture and plating

Human acute promyelocytic leukemia cells NB4, lung cancer cell line A549 and breast cancer cells MCF-7 were used in this study. NB4 cells were maintained in RPMI 1640 + GlutaMAX medium supplemented with 10% fetal bovine serum, 100 U/ml penicillin and 100 mg/ml streptomycin (Gibco, Grand Island, NY, USA) in a humidified incubator at 37 ºC with 5% CO_2_. A549 and MCF-7 cells were cultivated at the same conditions in DMEM + GlutaMAX medium (Gibco, Grand Island, NY, USA) with indicated supplements. For growth inhibition (GI) assessment exponentially growing cells were seeded in 96 well plates at a density of 0.5∙10^6^ cells/ml (NB4) and in 96 well plates at a density 1.0∙10^4^ (A549 and MCF-7) cells per well. For cell cycle and apoptosis analysis cells were seeded in 6 well plates at a density of 1∙10^6^ (NB4) and 0.45∙10^6^ (A549 and MCF-7) cells per well. For immunocytochemical analysis cells were seeded in 24 well plates at a density of 0.25∙10^6^ (NB4 in suspension, mounted on cover-slips after labeling with DAPI) and 0.1∙10^6^ (A549 and MCF-7 directly on cover-slips) cells per well.

### Chemosensitivity testing

Amyloids were tested in triplicates at different concentrations in the range of 0.5−5 μM. For negative control, cells were treated with an equivalent amount of solvent (v/v). For NB4, A549 and MCF-7 cell viability evaluation MTT assay was used. Drug treatment was started 24 h after plating. Cells were incubated with agents for 24 and 48 hours. Then cell culture medium was replaced with medium without phenol red and MTT reagent (Sigma, St. Louis, USA) was added to the medium (20% of the culture medium volume from 5 mg/ml stock solution) and cells were further incubated for 2 h. After incubation, the medium was discarded and 50 μl of DMSO was added to each well. The absorbance of each well was measured at 540 nm using Infinite M200 PRO (Tecan, Mannedorf, Switzerland) spectrometer. Data was expressed as GI (%) values with SDs.

### Cell cycle analysis by DNA content

Cells (1–1.5∙10^6^ cells/sample) were washed twice by centrifugation (200 rcf, 5 min, 4°C) in PBS, then resuspended in 1 ml of ice cold PBS. Later they were vortexed gently, dropwise slowly adding 3.3 ml of cold absolute ethanol and stored at -20°C overnight. Before staining cells were centrifuged at 200 rcf, 10 min, 4°C, washed twice with cold PBS, then resuspended in 500 μl PBS, containing 50 μg/ml PI and 50 μg/ml RNAse A (Sigma, St. Louis, USA) and incubated for 30 min in the dark at 37°C. Specimens were flow cytometrically analyzed with FACSCanto II, using FACSDiva Software 6.0 (BD Biosciences, CA, USA). Ten thousands events were gathered for each sample. Non-specific fluorescence was monitored using PI untreated control cells.

### Cell death analysis using Annexin V binding

Cells were harvested by centrifugation (200 rcf, 5 min, 4°C) and washed with PBS. For cell death evaluation the ApoFlowEx^®^ FITC Kit (EXBIO, Prague, Czech Republic) was used. Annexin V staining was performed according to the manufacturer‘s instructions. Specimens were flow cytometrically analyzed with FACSCanto II, using FACSDiva Software 6.0. Ten thousands events were gathered for each sample. Non-specific fluorescence was monitored using Annexin V and PI untreated control cells.

### Fluorescence analysis

For amyloid localization in human cancer cells the NB4, A549 and MCF-7 cell lines were cultivated with FAM-labeled amyloids for 4, 24 and 48 hours. After indicated time of treatment cells were rinsed three times with phosphate buffer (PBS, pH 7.5) and fixed for 15 min in PBS supplemented with 4% (w/v) paraformaldehyde. Then cells were rinsed again for three times with PBS (pH 7.5) and permeabilized for 20 min in 0.2% Triton-X/PBS. After incubation cells were rinsed four times with 1% PBS/BSA and nuclei were labeled using DAPI for 10 min. After incubation cells were rinsed five times with 1% PBS/BSA. Cover-slips with cells were dried and mounted on slides and analyzed with Zeiss Axio Observer Z1 (Carl Zeiss, Germany).

### Statistical analysis

The data are presented as means ± SD of three or more independent experiments. For MTT statistical analysis two-way ANOVA was used in GraphPad Prism.

## Results

### Characterization of the size, morphology and molecular properties of amyloid oligomers

**Fig 1:**
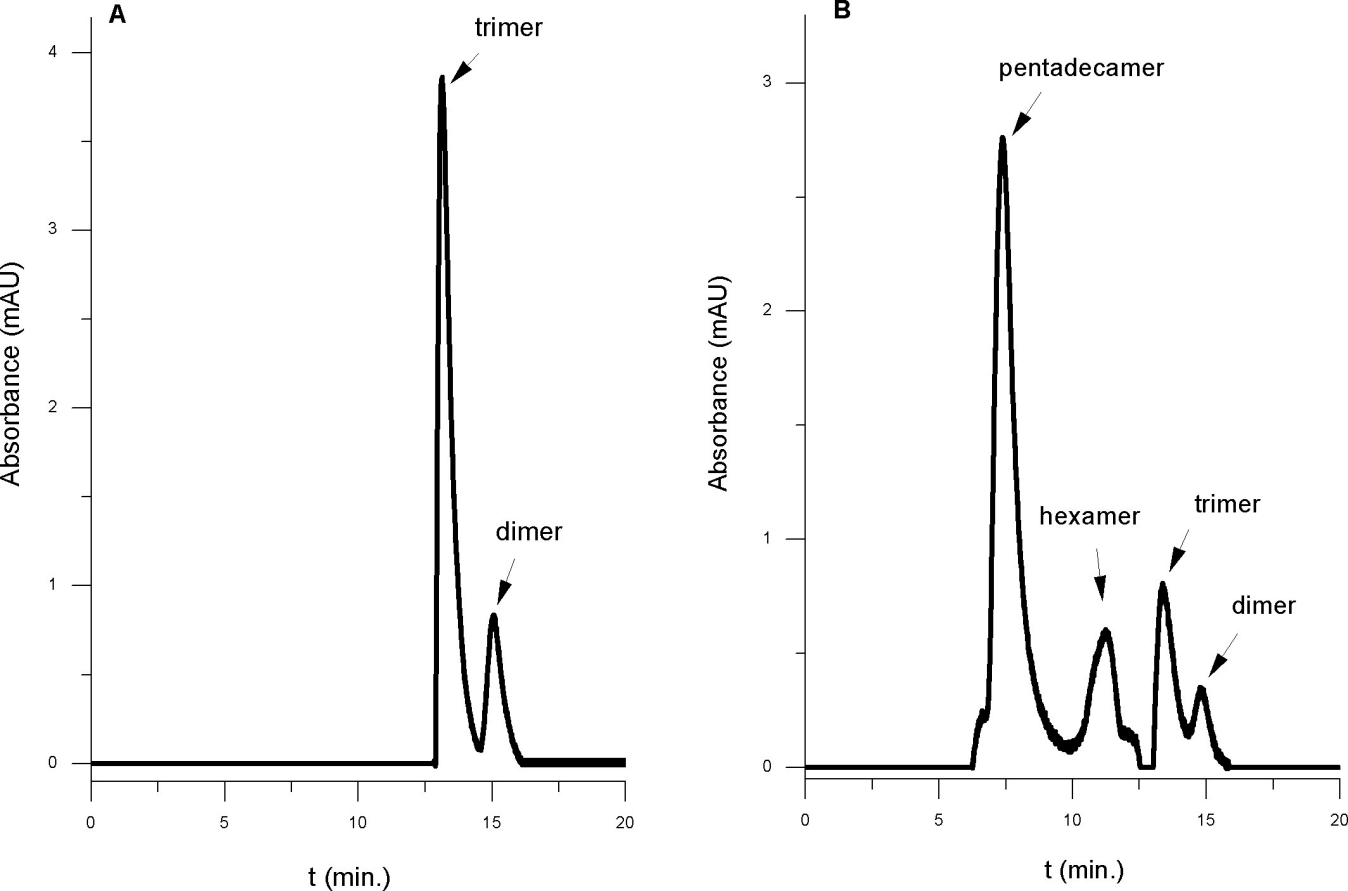
SEC–HPLC chromatograms of amyloids preparation. (A)–Aβ(1–42)–HFIP protocol preparation; (B)–Aβ(1–42)–HFIP-free protocol preparation. The sample concentration was 10 μM. Mobile phase– 0.1 M NaCl, 0.01 M NaH_2_PO_4_ buffer at pH 7.4 and flow rate of 0.25 ml/min.

We applied SEC HPLC to estimate the approximate molecular size distributions of Aβ(1–42) oligomer species. As shown in Fig 1, HFIP–inhibited amyloid species comprise a mixture of dimers and trimers of Aβ(1–42) (Fig 1A). The trimer peak is nearly 3-fold larger suggesting trimer as a dominant component of the HFIP-inhibited mixture. The exact molar ratio of oligomers remains unknown because the extinction coefficients of the particular amyloid component are not known. The elution profile changes significantly in the absence of the oligomerization inhibitor HFIP (Fig 1B). In this case, the dominant peak eluting at t ≈ 7.1 min corresponds to oligomers with the approximate MW 67 kDa (see supporting material S1 Appendix for the HPLC molecular weight calibration experiments). This peak is most likely dominated by a significant amount of 15-mers of Aβ(1–42) in the tested mixtures. Alongside smaller species, presumably, hexamers, trimers and dimers are also present at lower concentration levels.

**Fig 2:**
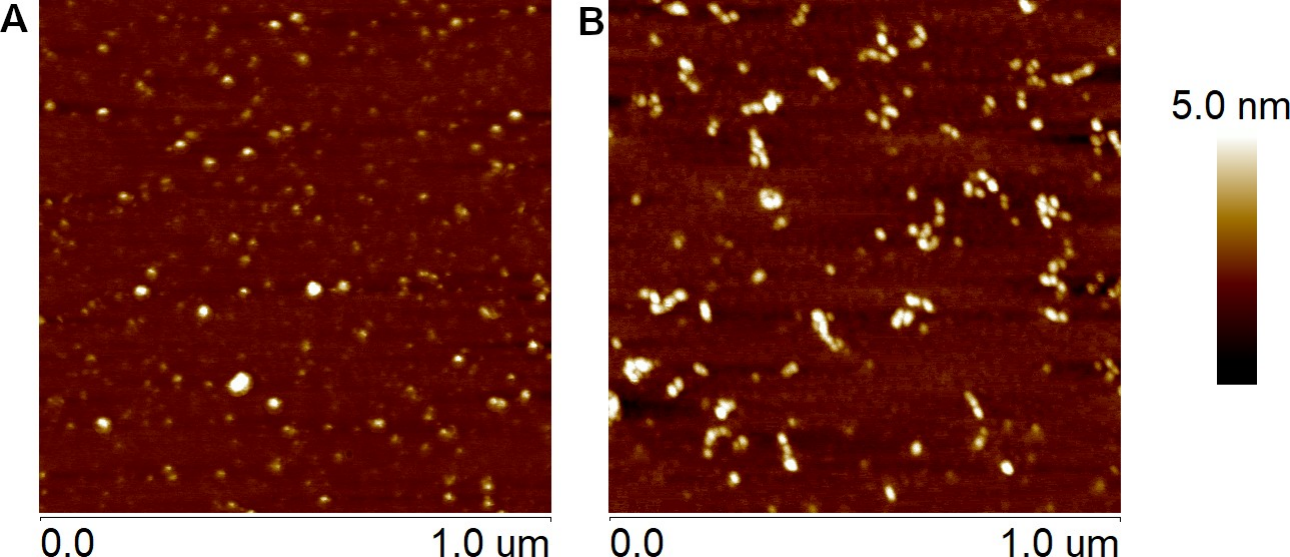
**Representative AFM images of adsorbed 10 μM Aβ(1–42) oligomers prepared by HFIP protocol (A) and HFIP-free protocol (B).** Mica surfaces (1 μm^2^) were visualized after 10 min incubation with preparations.

**Fig 3:**
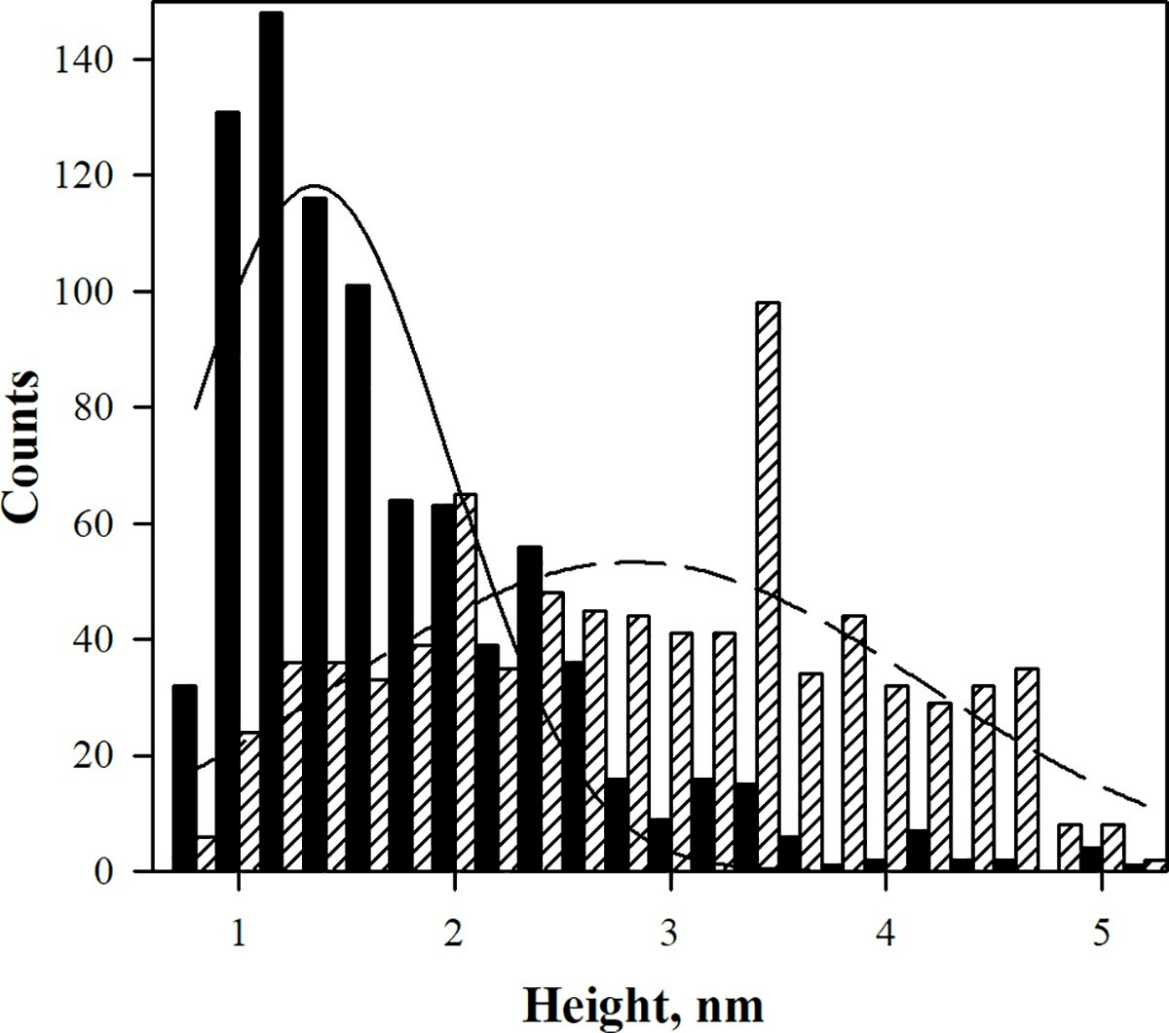
The actual height and Gauss approximation distributions of Aβ(1–42) adsorbed on mica. HFIP-protocol (*black* box and line) and HFIP-free protocol (box with diagonal lines and dashed line).

We probed the morphology of differently assembled amyloid β oligomers by the AFM. Fig 2 displays typical morphological features obtained using amyloid preparations by the HFIP and HFIP-free protocols. A broad range of structures including protofibrils, which contain bead by bead assemblies of multiple globules, and small, spherical oligomers was visualized for both protocols. To evaluate differences produced by these two protocols the topography images were analyzed by the Particle Analysis feature with Nanoscope software. Data analyses are summarized in Fig 3. The height distribution revealed that the average height of Aβ(1–42) produced by the HFIP protocol is 1.73 ± 0.68 nm and the HFIP-free protocol, 2.76 ± 1.04 nm, respectively, Fig 3, in preparations which were assemble under the same condition on the same day as ones used for HPLC experiment. We must acknowledge that the AFM data includes significant standard deviations. Also, in some experimental data sets we observed very similar height distributions (such like shown in Fig 3) for both types of amyloid preparations. These facts may cast doubt on the distinguishability of two types of preparations by the AFM methodology, which in contrast to HPLC uses samples obtained by the adsorption on mica process. Centrifuge filtering though the 100 kDa filter essentially did not affect amyloid size distributions of amyloid preparations as it can be seen from the data in supporting material S2 Appendix.

**Fig 4:**
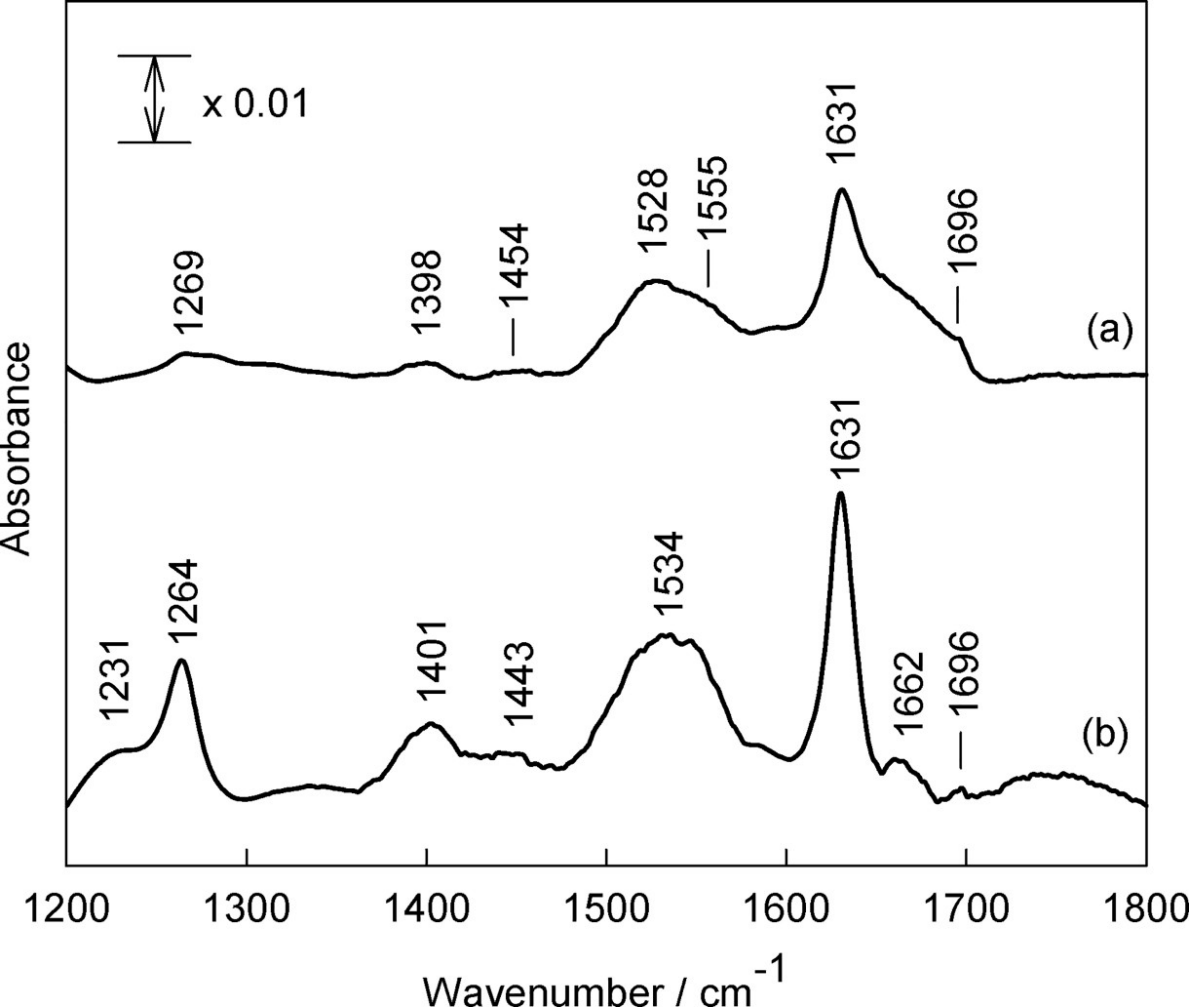
FTIR spectra of Aβ(1–42) oligomers of different size. FTIR spectra of HFIP protocol (a) and HFIP-free protocol (b) Aβ(1–42) oligomers deposited at CaF_2_ substrate in the spectral region of 1200−1800 cm^−1^.

**Fig 5:**
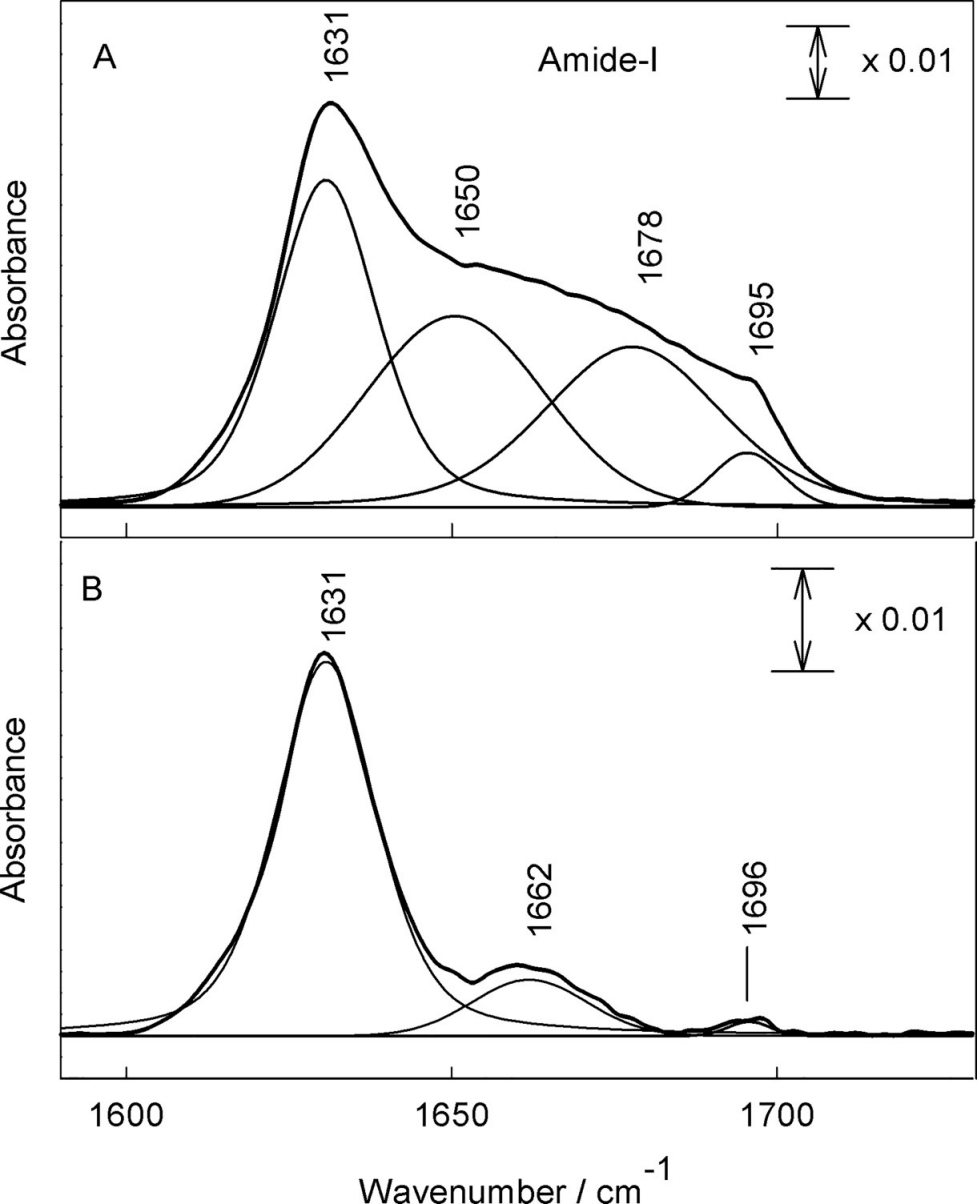
Comparison of Aβ(1–42) oligomers in Amide-I spectral region. FTIR absorption spectra with fitted Gaussian-Lorentzian form components in Amide-I spectral region: (A) spectra of Aβ(1–42)–HFIP protocol, and (B) Aβ(1–42)–HFIP-free protocol; both deposited at CaF_2_ substrate.

**Fig 6:**
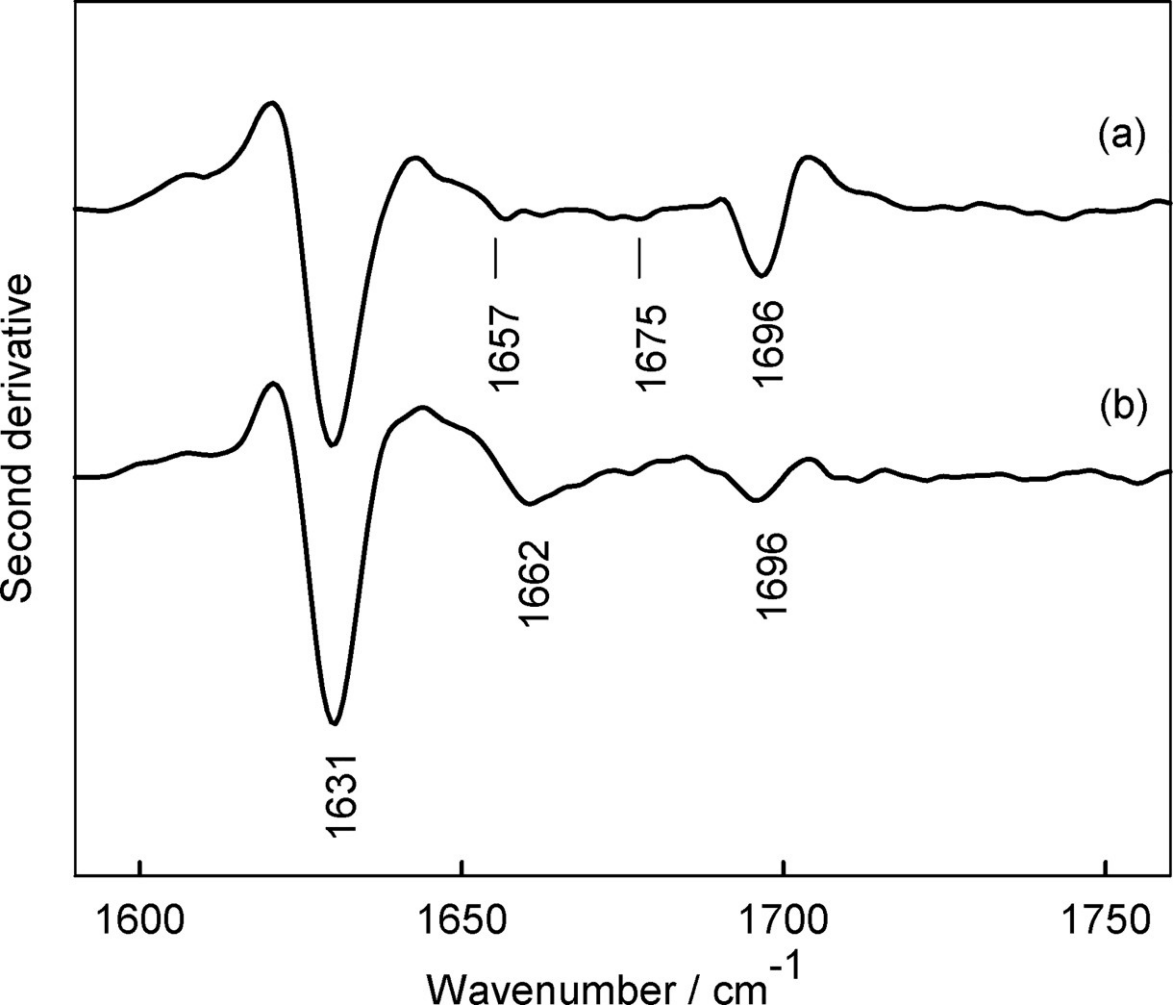
Second derivative spectra of Amide-I band. (a) Amide-I band of HFIP protocol, and (b) HFIP-free protocol of Aβ(1–42) oligomers deposited at CaF_2_ substrate in the spectral region of 1590−1760 cm^−1^.

The molecular structure of β-amyloid oligomers has been probed by infrared spectroscopy. Fig 4 compares FTIR spectra of oligomers prepared by HFIP and HFIP-free protocol in the frequency region from 1200 to 1800 cm^–1^. The amide group vibrational modes are visible near 1231–1269 (Amide-III), 1528–1555 (Amide-II), and 1631–1696 cm^–1^ (Amide-I) [25–30]. The broad band near 1398–1401 cm^–1^ is due to an asymmetric deformation vibration, δ_as_(CH_3_), of side chains (Ala, Ile, Leu, Val) methyl groups [31]. The low intensity bands near 1443–1454 cm^–1^ correspond to scissoring deformation vibration of side chains methylene groups, δ(CH_2_). Vibrational modes of amide group are sensitive to the secondary structure conformation [25–30]. Thus, inspection of Amide-II spectral region of Aβ(1–42) oligomers prepared by the HFIP protocol (small aggregates) (Fig 4(A)) reveals presence of the dominant band near 1528 cm^–1^ due to the β-sheet secondary structure and shoulder near 1555 cm^–1^ associated with α-helix conformation [32]. In the case of large Aβ(1–42) oligomers (prepared by HFIP-free protocol) the broad band centered near 1534 cm^–1^ dominates in the Amide-II spectral region (Fig 4(B)) indicating that β-sheet conformation prevails for this type of peptide aggregates. More quantitative analysis of secondary structure conformations was prepared by considering the Amide-I spectral region. It is accepted that Amide-I mode is more useful for secondary structure determination [26]. Amide-II mode arises from highly coupled N–H bending (60%) and C–N stretching (40%) vibrations, while the Amide-I vibrational mode is considerably less coupled; this mode is associated with predominant C = O stretching vibration (80%) with minor contribution from C–N out-of-phase stretching motion, C–C–N deformation, and N–H in-plane bending vibrations [26]. Fig 5 compares FTIR spectra of Aβ(1–42) oligomers formed using different protocols in the Amide-I spectral region. Experimental spectra were fitted with Gaussian-Lorentzian form components, which can be assigned to different secondary structure elements of peptides [26, 31, 33]. To determine the approximate position of fitted components, the second derivative spectra were calculated (Fig 6). The relative fraction of secondary structure elements derived from the analysis of Amide-I band and assignments of the components are given in Table 1. In both cases β-sheet is a dominant motive, visible as an intense band at 1631 cm^–1^ and less intensity high frequency band near 1695–1696 cm^–1^. Presence of the high frequency component is an indication of antiparallel β-sheet configuration [34−36]. The relative fraction of β-sheet components determined from the integrated intensities of corresponding infrared bands increases from 42.9 ± 2.0 to 79.4 ± 9.4% comparing small and larger oligomers, respectively (Table 1). Importantly, small Aβ(1–42) oligomers exhibit considerably higher integrated intensity of α-helical structural domain in comparison with larger ones, 36.6 ± 3.0% and 20.5 ± 9.4%, respectively.

**Table 1:** Amide-I peak positions and integrated intensities with corresponding band assignments of Aβ(1–42) peptide.

Peptide Aβ(1–42)	α-helix cm^−1^ (%)	β-sheet cm^−1^ (%)	β-sheet organization index	Unordered helix + random cm^−1^ (%)
Aβ(1–42)—HFIP protocol	1650 (36.6 ± 3.0)	1631 / 1696 (42.9 ± 2.0)	0.154 ± 0.02	1678 (20.4 ± 5.1)
Aβ(1–42)—HFIP-free protocol	1662 (20.5 ± 9.4)	1631 / 1695 (79.4 ± 9.4)	0.054 ± 0.02	− (−)

FTIR spectra obtained on centrifuge-filtered samples showed no essential differences in the secondary structure of amyloid species. More details can be found in the supporting material S3 Appendix.

### Effect of Amyloid species on cancer cells

#### Cancer cell growth inhibition after treatment with amyloids

Prepared amyloids were tested for their antiproliferative activity using three different human cancer cell lines: acute promyelocytic leukemia cells NB4, lung cancer cell line A549 and breast cancer cells MCF-7. After 24 and 48 hour treatment, the effect of compounds was evaluated using MTT assay according to the manufacturer’s instructions as described in Materials and Methods section.

**Fig 7:**
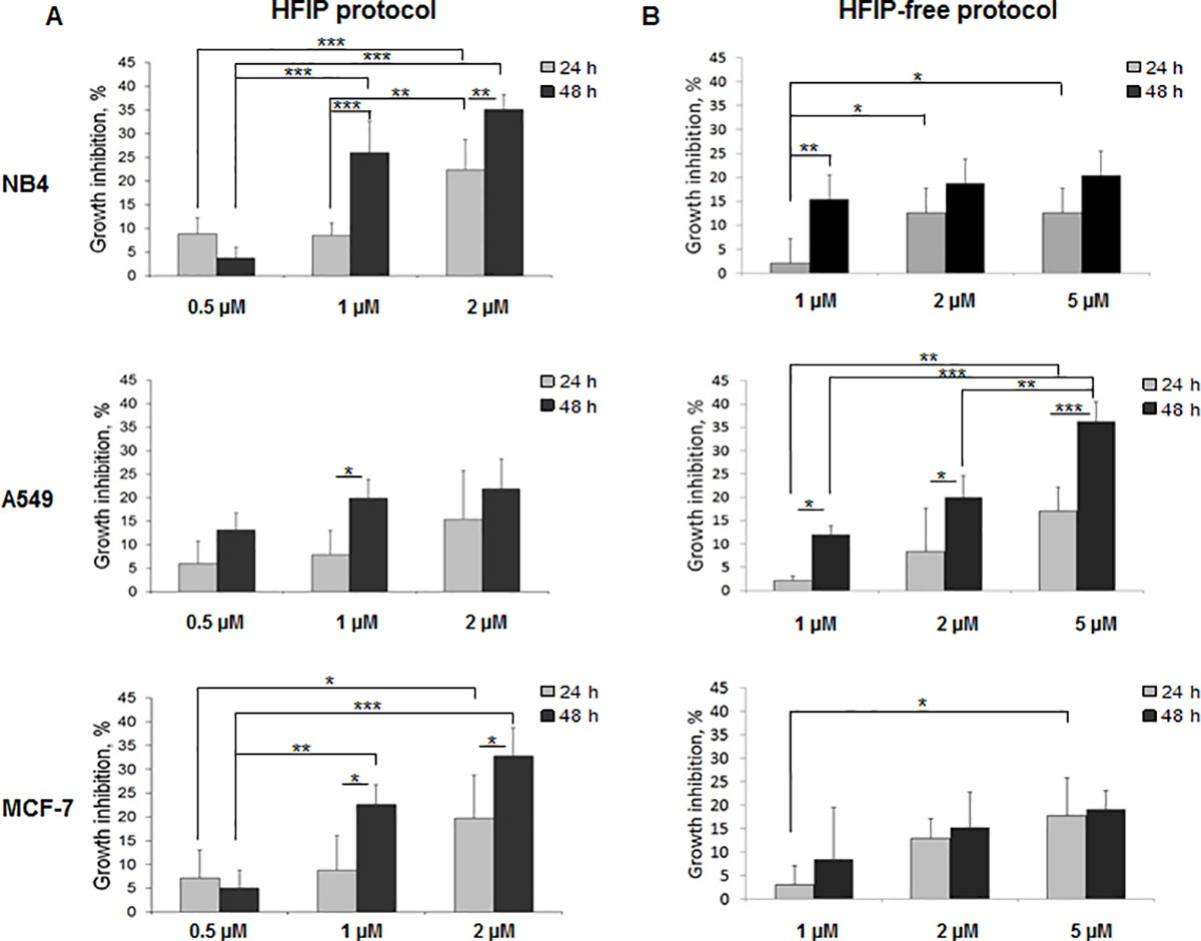
Evaluation of growth inhibition of cancer cell lines treated with amyloids. The anti-proliferative activities of amyloids were tested using the MTT assay as previously described. (A)–growth inhibition of cells treated with HFIP protocol amyloids, concentration range 0.5–2 μM. (B)–growth inhibition of cells treated with HFIP-free protocol amyloids, concentration range 1–5 μM. P ≤0.05 (*), P ≤0.01 (**), P ≤0.001 (***) indicate significant differences, if not indicated otherwise the difference was not significant.

Obtained results (Fig 7) indicate that amyloids prepared by using HFIP protocol inhibited cancer cell growth more in comparison with amyloids, prepared by using HFIP-free protocol. Regarding the differences of amyloid preparation protocols more statistically significant effects were observed on NB4 and MCF-7 cells when using HFIP protocol amyloids, in contrast the effects of HFIP-free protocol amyloids were more statistically significant on A549 cells. We detected that after 24 hour treatment with 2 μM of HFIP protocol amyloids cell growth in NB4 and MCF-7 cell lines was inhibited by 20%, whereas after 24 hour treatment with HFIP-free protocol amyloids growth inhibition reached 13%. The same tendencies were observed after 48 hour treatment, the cell growth inhibition was increased up to 2 times when cells were treated with Aβ oligomers, prepared using HFIP protocol.

In summary, all used Aβ oligomer preparations were non-toxic (the observed growth inhibition didn’t exceed 50% after 48 h) to investigated cancer cell lines. However, the decrease in cell proliferation activity was observed after treatment with Aβ oligomers, prepared using HFIP and HFIP-free protocols.

#### The effect of amyloids on cell cycle distribution

**Fig 8:**
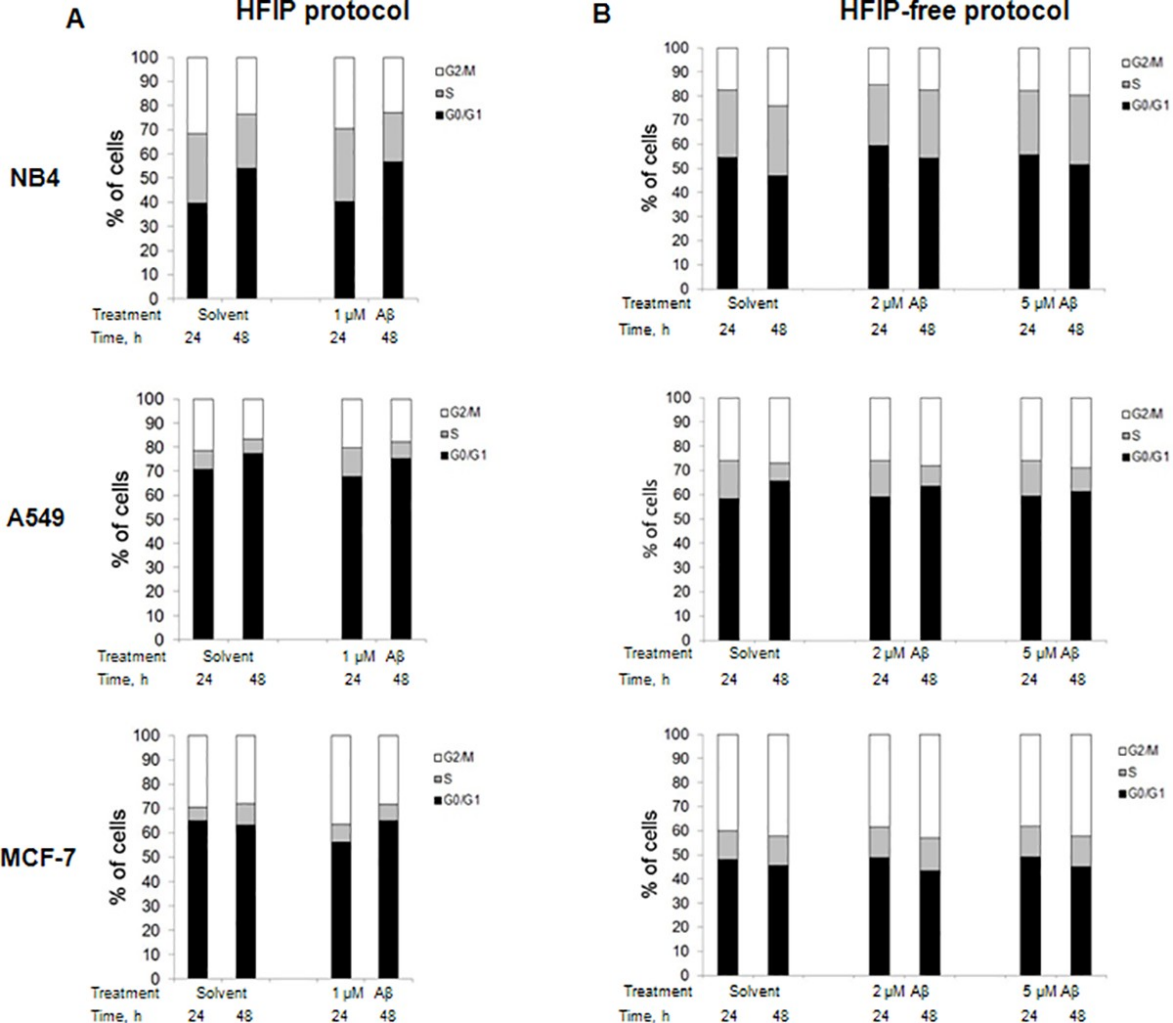
Distribution of cell cycle phases of cancer cells treated with amyloids. Amyloid activity on cell cycle distribution was analyzed by flow cytometry, as detailed in the Materials and methods section. (A)–cell cycle of cells treated with 1 μM HFIP protocol amyloids and solvent for negative control. (B)–cell cycle of cells treated with 2–5 μM HFIP-free protocol amyloids and solvent for negative control.

In order to determine, if cell growth inhibition was mediated by the cell cycle arrest, flow cytometric cell cycle analysis was performed. NB4, A549 and MCF-7 cells were exposed to 1 μM Aβ oligomers prepared by the HFIP protocol or to 2 μM and 5 μM Aβ oligomers prepared by the HFIP-free protocol. Control cells were incubated with appropriate amount of solvent. Results are presented in Fig 8.

It was shown that HFIP protocol preparations induced a slight NB4 cells arrest in G0/G1 cell cycle phase. After 48 hour incubation with 1 μM of Aβ oligomers G0/G1 cell cycle phase was increased app. by 3% (compared to solvent control). The same effect was evident for amyloids prepared using HFIP-free protocol. For instance, after 48 hour treatment with 2 μM Aβ oligomers of HFIP-free protocol, the arrest of NB4 cells in G0/G1 cell cycle phase was greater than in solvent control app. by 7%.

We demonstrated that in human lung cancer cell line A549 after 24 hour treatment with amyloids prepared by HFIP protocol the cell accumulation in S phase was increased app. by 4% and after 48-hours of treatment cell arrest in G2/M cell cycle phase was observed. The effect of amyloids prepared by the HFIP-free protocol also had a mild tendency to arrest A549 cells in S phase. In addition, the same tendency of cell arrest in G2/M phase was registered. After 48-hour treatment with 2 μM and 5 μM of amyloids (HFIP-free protocol) cell accumulation in G2/M phase increased by 2–3% compared to solvent control.

MCF-7 cells treated with 1 μM amyloids (HFIP protocol) for 24 hours showed cell arrest in G2/M phase (more than 7% compared to solvent control). In contrast, HFIP-free protocol amyloids showed tendency to arrest MCF-7 cells in G0/G1 cell cycle phase after 24 hours of treatment. In general, Aβ oligomer effect on cancer cell cycle distribution was not very significant. Only a mild tendency of NB4 cell cycle arrest in G0/G1 cell cycle phase was observed after treatment with Aβ oligomers prepared using HFIP and HFIP-free protocols. In contrast, in lung cancer cells A549, as well as in breast cancer cells MCF-7, Aβ oligomers slightly increased cell accumulation in S or G2/M phases.

#### Cell death of cancer cells treated with amyloids

All cancer cell lines were treated with 2 μM HFIP protocol amyloids and 5 μM HFIP-free protocol amyloids for 24 and 48 hours. Control cells were incubated with appropriate amount of solvent.

**Fig 9:**
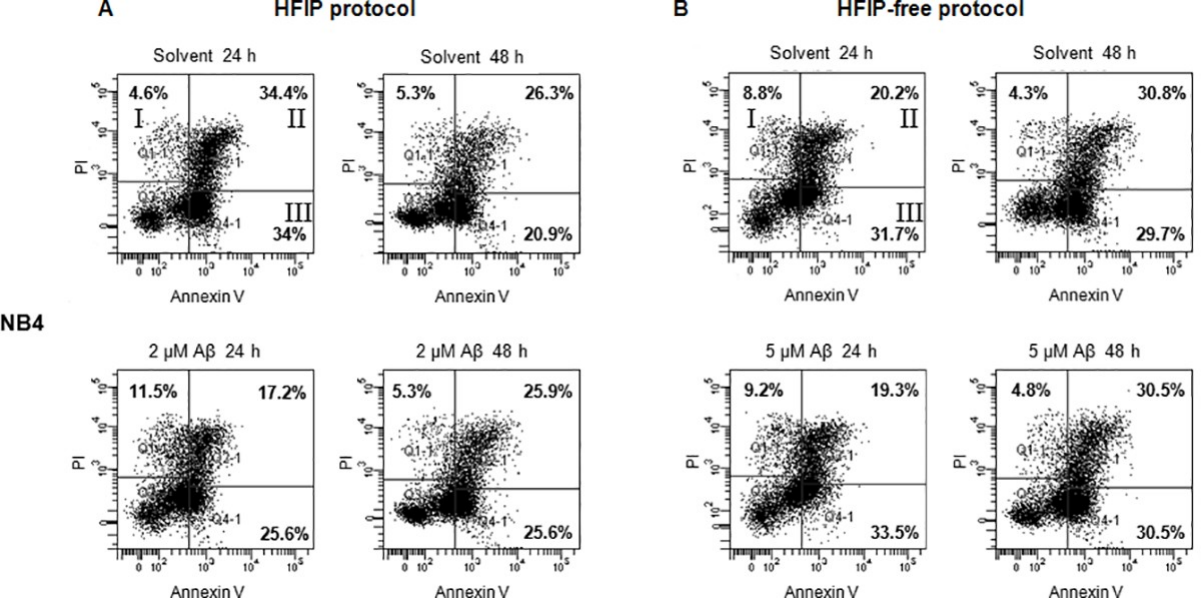
Induction of cancer cell death by amyloids. The pathway of cell death induced by amyloids was analyzed by flow cytometry. Representative scatter plots showing Annexin V and PI staining of NB4 cells, I–necrosis (Annexin V-/PI+), II–late apoptosis (Annexin V+/PI+), III–early apoptosis (Annexin V+/PI-). (A)–NB4 cells treated with 2 μM of HFIP protocol amyloids and solvent for negative control. (B)–NB4 cancer cells treated with 5 μM of HFIP-free protocol amyloids and solvent for negative control.

Obtained data indicate, that Aβ oligomers are nontoxic to investigated cancer cell lines. In NB4 cells after treatment with HFIP protocol preparations only a mild increase in necrotic cell subpopulation (Annexin V-/PI+) was detected (Fig 9A): after 24 hours treatment with 2 μM Aβ oligomers number of necrotic cells increased approximately by 7%. The observed tendencies of solvent and amyloid activity on early (Annexin V+/PI-) and late apoptosis (Annexin V+/PI+) do not indicate that amyloid preparations play major role in NB4, A549 and MCF-7 cells apoptotic death (see S4 Appendix).

#### Fluorescence analysis of cancer cells treated with amyloids

**Fig 10:**
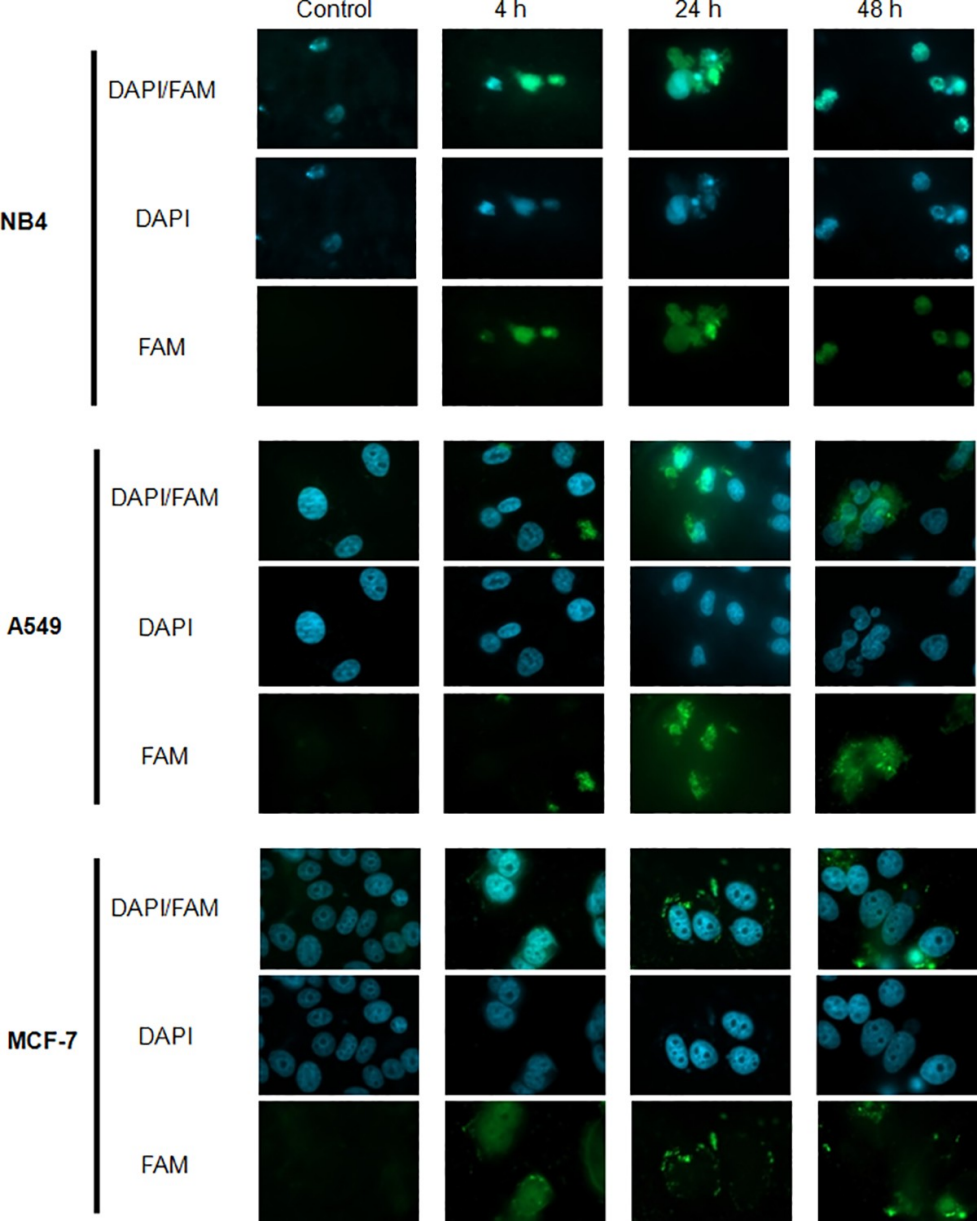
Fluorescence analysis of cancer cells with FAM-labeled amyloids. Amyloid accumulation in cancer cells after certain periods of time (4, 24, 48 h) and control cells without amyloid treatment. FAM-labeled amyloids are shown in green, DAPI stained nuclei are shown in blue. Magnification x63.

To elucidate the accumulation areas of amyloids in cancer cells, NB4, A549 and MCF-7 cell lines were cultivated with FAM-labeled amyloids for 4, 24 and 48 hours. Fig 10 presents fluorescent microscopy images. MCF-7 cell specimens show how amyloids hovered over the cells after 4, 24 and 48 hours of incubation and with time aggregated into the cells membrane. This kind of behavior of amyloids was not very intensive in A549 cell line, but still could be detected. In contrast, in NB4 cells FAM-labeled amyloids were detected mainly in the area of the nucleus.

## Discussion

Our HPLC data indicate clear effect of HFIP on oligomerization outcome. As seen in Fig 1, the HFIP-free medium produces a range of amyloid species spanning from dimers to pentadecamers. The molecular weight estimate performed via calibration of the chromatographic column as described in the supporting material S1 Appendix suggests pentadecamers are the main component of the HFIP-free amyloid preparation. In contrast, HPLC data indicates just two major components in HFIP assembled preparations. According to calibration (see S1 Appendix) those components are dimer and trimer with the clear dominance of the latter form of oligomer.

The size of amyloid β(1–42) monomer in solution was reported and its hydrodynamic radius was found to be ~ 1.7 nm [37]. The group of Lyubchenko [38] reported images of cross-linked amyloid β(1–42) (Phe 10, Tyr42) oligomers (monomers–decamers) adsorbed on modified mica surface by AFM. The height for the monomer, trimer and pentamer as visualised by AFM was found to be approx. 0.6, 0.8 and 1.2 nm, respectively [38]. Our AFM data set presented in Fig 2 clearly indicates a dominance of smallest species, presumable monomers, dimers and trimers in amyloid deposits on mica (Fig 3). In contrast, HFIP-free preparations are dominated by species with AFM heights from 2.5 to 3.5 nm. Very likely this interval may correspond to pentadecamer population, which, as shown by the HPLC data, is dominant fraction in solution. As noted before AFM data exhibits poor reproducibility compared to the HPLC, so the HPLC methodology is preferable for the precise characterization of the amyloid oligomer species in mixtures.

As shown earlier, small 3−5 nm oligomers assembled in the presence of HFIP exhibited significant toxicity in the primary mixed neuronal cultures [20]. In contrast, large (>5 nm) spherical oligomers triggered no necrosis, however, facilitated long-term autophagy effects [20]. Both oligomer forms were dominated by the β-sheet (43%) and disordered (32%) peptide fragments [20]. In the current work, FTIR data indicates similar secondary structure content (39% β-sheet, and 29% disordered) in HFIP prepared β-amyloid oligomers (Fig 5). However, HFIP-free oligomers exhibit quite different secondary structure, which is dominated at 87% by the β-sheet peptide fragments with the below detection limit content of the disordered structures. In addition, oligomers prepared with HFIP tend to form higher amount of α-helix (32%), compared to HFIP-free oligomers containing only 20% of α-helix structures.

Recent structure-toxicity studies have revealed clear correlation between the amyloid toxicity and presence of antiparallel β-sheet secondary structure; peptides with parallel β-sheet configuration are non-toxic [36, 39−41]. While secondary structure of small (toxic, 1−2 nm) and larger (non-toxic, 4−5 nm) oligomers can be sensitively probed by CD spectroscopy, this technique is not able to discriminate the antiparallel and parallel β-sheet configuration [20]. In contrast, FTIR spectroscopy provides a possibility to determine the so-called β-sheet organization index (A_1695_/A_1631_) which correlates with the relative amount of antiparallel β-sheet structure [41]. Table 1 indicates nearly 3-fold higher β-sheet organization index for Aβ(1–42) oligomers prepared using HFIP. This allows us to conclude that the content of antiparallel β-sheet structural elements is higher in HFIP-assembled compared to HFIP-free β-amyloid oligomers. Conversely, parallel β-sheet structure is more abundant in larger peptides, consisting approximately of 15 monomers.

In conclusion, FTIR along with the size exclusion HPLC data shows that the HFIP assembled oligomers are dominated by the relatively low molecular mass species (dimers, trimers). Our attempt to apply centrifuge-filtering using MWCO 100 kDa indicated no significant changes neither to FTIR data (S3 Appendix) nor to AFM oligomer size distributions (S2 Appendix), which suggests that if large (>150 kDa, according to HPLC data) oligomers were present in amyloid preparations their contribution to physical parameters of oligomer mixtures was miniscule. According to the FTIR data the antiparallel, intramolecular β-sheets dominate HFIP-assembled oligomers (Table 1, β-sheet organization index). In contrast, HFIP-free oligomers consist mainly of pentadecamers with some hexamers and smaller species. This preparation is dominated by parallel intermolecular β-sheets (Table 1, β-sheet organization index), which are the main structural component of HFIP-free oligomers.

The amyloid preparations were characterized right before starting the biological experiments. One cannot preclude possibility of oligomerization processes was continuing in biological media during the time span of the biological tests. Even though such process cannot be excluded we argue that the processes in biological buffer were significantly slower because of the dilution of the samples. In biological medium the monomer based concentration of Aβ(1–42) was from 20 to 200 times smaller than in oligomerization buffers. Also, both buffers contained similar concentrations of inorganic salts. Nevertheless, biological buffer composition as a factor that may influence action of oligomer preparations described in the current study needs to be taken into account if the reproduction of our results would be attempted. Also, upon request by one of the reviewers we attempted to test if the presence of large oligomers (>150 kDa as measured by HPLC) may affect biological effects observed in the current study. Consistent with physical characterization data we observed only minimal quantitative differences in cell culture responses to both 0.22 μm and 150 kDa filtered amyloid samples (see S5 Appendix for details). Such results indicate minor biological effects of larger than 150 kDa oligomeric species at concentrations which were beyond the detection limits of techniques used in the current work.

It is known that β-amyloids affect cells, especially neurons, and can cause various diseases [42–44]. The effects are different, while the structural peculiarities of the species which were used for testing remained mainly unexplored. One of the most studied effect of amyloids on cells is cell cytotoxicity. Several studies demonstrated cytotoxicity in neurons and neuroblastoma cells [20, 45–48]. Our study shows that human hematological (NB4) and solid (A549, lung and MCF-7, breast) cancer cells are sensitive to β-amyloids as well (Fig 7). We have shown that the level of sensitivity depends on the protocol of preparation of amyloids and the type of cell line. All three cancer cell lines we used were more sensitive to HFIP protocol amyloids, which we showed have distinct molecular structure and size (vide ultra).

Information about cell cycle distribution changes in cell cultures are affected by β-amyloids is scarce. Frasca and colleagues [49] demonstrated that β-amyloid peptide fragment (25–35) modified neuroblastoma cell cycle profiles by markedly increasing the number of cells in the S phase and reducing the population of the G2/M phase. Bhaskar et al. [50] showed that primary cultures of cortical neurons incubated with Aβ(1–42) oligomers were able to re-enter the cell cycle. In addition, this phenomenon was associated with activation of Akt and mTOR signaling pathways.

In this study, we determined that the phase of the cancer cell cycle arrest upon treatment with Aβ oligomers does not depend on the preparation protocol ‒ it lies in cancer cell line itself, since all cell lines demonstrated different manner. In addition, a slight induction of NB4 cell necrosis after 24 hour treatment with HFIP protocol preparations was observed. This data is consistent with previous studies in other cell lines [51, 52]. Results of fluorescence analysis suggest that β-amyloids maintain a tendency to aggregate into A549 and MCF-7 cancer cell membranes. This could lead to membrane disruption and loss of cell viability [22, 53]. However, in NB4 cells FAM-labeled amyloids were mainly detected in the area of the nucleus. Such phenomenon may be explained (at least partially) due to the differences in plasma membrane composition of investigated cell lines, intrinsic characteristics of different cancer cells or may be related to the type specificity of cancer cells (hematological or solid). The results of FAM-labeled amyloid localization in NB4 cells also correlate with growth inhibition, cell cycle analysis and Annexin V and PI staining results, indicating different and more pronounced effect of β-amyloids on NB4 cells, compared to cells of solid tumors.

It is known that amyloid precursor protein (APP) and its family members amyloid precursor-like protein 2 (APLP2) expression is aberrantly altered in many types of cancers, e.g., pancreatic, colon, breast, prostate, lung, and others. Moreover, these proteins participate in various molecular pathways in cancer cells, including both pro-growth and pro-invasion functions [54]. In addition, some cancers (especially hepatic) were shown to be associated with increased peripheral blood levels of β-amyloids [55]. Epidemiological studies have revealed that patients with a history of cancer have a slightly lower risk of developing Alzheimer’s disease and inversely, patients with Alzheimer’s disease are not so cancer susceptible [56–58]. Such inverse epidemiological relationship between cancer and Alzheimer’s disease raises a question about biological mechanisms that interconnects both conditions. However, data related to β-amyloids effects on cancer cells, especially, in the context of the structural features of the amyloid species are lacking. Our results demonstrate that β-amyloids, especially ones with relatively low content of β-sheet structures but high antiparallel β-sheet organization index inhibit growth of hematological and solid cancer cells. The inhibition effect itself differs for different cell lines, which obviously related to peculiar characteristics of different cancer cells.

## Supporting information

S1 Appendix SEC HPLC column calibration. (DOCX)

S2 Appendix Effect of centrifuge filtering to morphology of amyloid oligomers prepared using HFIP protocol. (DOCX)

S3 Appendix Effect of centrifuge filtering with MECO 150 kDa on the secondary structure of amyloid oligomers. (DOCX)

S4 Appendix Cell death in cancer cells after amyloid treatment. (DOCX)

S5 Appendix Effect of centrifuge filtered amyloid preparations on cancer cell growth. (DOCX)

S1 Dataset (XLSX)

S2 Dataset (DOCX)

S3 Dataset (XLSX)

S4 Dataset (DOCX)

S5 Dataset (PPTX)
