# Supplementary material for: Amyloid β oligomers inhibit growth of human cancer cells
Source: PLoS One. 2019 Sep 11;14(9):e0221563. doi: 10.1371/journal.pone.0221563 (PMC6738617; doi:10.1371/journal.pone.0221563)
Supplement: S1 Appendix — (DOCX) [file pone.0221563.s001.docx]

Amyloid β oligomers inhibit growth of human cancer cells

**Bozena Pavliukeviciene^1^, Aiste Zentelyte^2^, Marija Jankunec^1^, Giedre Valiuliene^2^, Martynas Talaikis^1^, Ruta Navakauskiene^2^, Gediminas Niaura^1^, Gintaras Valincius^1^***

^1^Department of Bioelectrochemistry and Biospectroscopy, Institute of Biochemistry, Life Sciences Center, Vilnius University, Vilnius, Lithuania

^2^Department of Molecular Cell Biology, Institute of Biochemistry, Life Sciences Center, Vilnius University, Vilnius, Lithuania

* gintaras.valincius@gmc.vu.lt

Supporting information

S1 Appendix: SEC HPLC column calibration.

The calibration of the SEC column was performed with seven standard proteins under optimized conditions (30 °C; proteins concentration was 10 µM; mobile phase used was 0.1 M NaCl, 0.01 M NaH_2_PO_4_ buffer at pH 7.4 at 0.25 ml/min at a pressure of 34 bar (or 496 psi); absorbance measured at 275 nm). The molecular weight and SEC HPLC evaluation time of the protein standards you can see in Table S1 or Fig S1.

BSA was purchased from Thermo Fisher Scientific. Thrombin, Peroxidase, Pepsine, Lysozyme, Cytochrome C were obtained from Sigma-Aldrich. Parasin was from the American Peptide Company (California, USA).

**Table S1.** Protein standards.

|  | ***MW, kDa*** | ***Evaluation time, min*** |
| --- | --- | --- |
| ***Thrombin*** | 72 | 7.06 |
| ***BSA*** | 66 | 7.275 |
| ***Peroxidase*** | 44 | 8.980833 |
| ***Pepsine*** | 34.62 | 9.375 |
| ***Lysozyme*** | 14.3 | 13.04 |
| ***Cytochrome C*** | 12 | 13.35 |
| ***Parasin*** | 2 | 20.501 |

**Fig S1. HPLC calibration of Bio SEC-3 column with seven protein standards:** Thrombin, BSA, Peroxidase, Pepsine, Lysozyme, Cytochrome C, Parasin. The protein concentration was 10 µM. Mobile phase – 0.1 M NaCl, 0.01 M NaH_2_PO_4_ buffer at pH 7.4, flow rate of 0.25 ml/min. The R-squared was is 0.9980891483.
