# Supplementary material for: Amyloid β oligomers inhibit growth of human cancer cells
Source: PLoS One. 2019 Sep 11;14(9):e0221563. doi: 10.1371/journal.pone.0221563 (PMC6738617; doi:10.1371/journal.pone.0221563)
Supplement: S2 Appendix — (DOCX) [file pone.0221563.s002.docx]

Amyloid β oligomers inhibit growth of human cancer cells

**Bozena Pavliukeviciene^1^, Aiste Zentelyte^2^, Marija Jankunec^1^, Giedre Valiuliene^2^, Martynas Talaikis^1^, Ruta Navakauskiene^2^, Gediminas Niaura^1^, Gintaras Valincius^1^***

^1^Department of Bioelectrochemistry and Biospectroscopy, Institute of Biochemistry, Life Sciences Center, Vilnius University, Vilnius, Lithuania

^2^Department of Molecular Cell Biology, Institute of Biochemistry, Life Sciences Center, Vilnius University, Vilnius, Lithuania

* gintaras.valincius@gmc.vu.lt

Supporting information

S2 Appendix: Effect of centrifuge filtering to morphology of amyloid oligomers prepared using HFIP protocol.

Experimental AFM data of HFIP oligomers obtained before and after centrifuge-filtering at 100 kDa is shown in Figure S1 A and B respectively. As it follows from the image height distribution analysis (Fig S1, C) filtering does no change the size distribution of amyloid oligomer species. The highest probabilities of occurencies are observed for species with the average sizes between 1.3 and 1.8 nm.


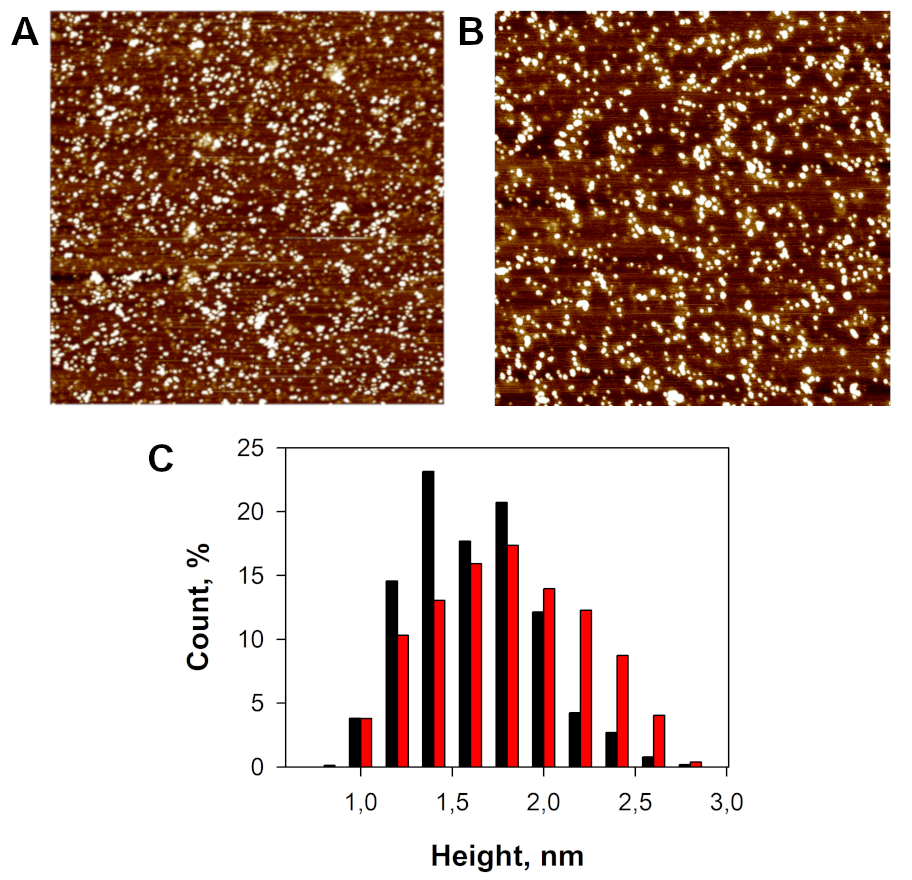

**Fig S1**. AFM topography images of aβ(1-42) prepared by HFIP protocol. A) non-filtered preparation 10.6 µM; B) filtered preparation 8.3 µM. Scan size 1 µm x 1 µm, 1024 pxl. C) The height distributions of non-filtered (*black* bars) and filtered (*red* bars) preparations.

Conclusion: centrifuge filtering does not significantly affect size distribution of oligomeric species prior and after centrifuge filtration.
