# Supplementary material for: Amyloid β oligomers inhibit growth of human cancer cells
Source: PLoS One. 2019 Sep 11;14(9):e0221563. doi: 10.1371/journal.pone.0221563 (PMC6738617; doi:10.1371/journal.pone.0221563)
Supplement: S3 Appendix — (DOCX) [file pone.0221563.s003.docx]

Amyloid β oligomers inhibit growth of human cancer cells

**Bozena Pavliukeviciene^1^, Aiste Zentelyte^2^, Marija Jankunec^1^, Giedre Valiuliene^2^, Martynas Talaikis^1^, Ruta Navakauskiene^2^, Gediminas Niaura^1^, Gintaras Valincius^1^***

^1^Department of Bioelectrochemistry and Biospectroscopy, Institute of Biochemistry, Life Sciences Center, Vilnius University, Vilnius, Lithuania

^2^Department of Molecular Cell Biology, Institute of Biochemistry, Life Sciences Center, Vilnius University, Vilnius, Lithuania

* gintaras.valincius@gmc.vu.lt

Supporting information

S3 Appendix: Effect of centrifuge filtering with MECO 150 kDa on the secondary structure of amyloid oligomers.





**Fig S1. Comparison of Aβ(1-42) oligomers in Amide-I spectral region.** FTIR absorption spectra with fitted Gaussian-Lorentzian form components in Amide-I spectral region: (A) spectra of Aβ(1-42) – HFIP protocol, and (B) Aβ(1-42) – HFIP-free protocol; both filtered and deposited at CaF_2_ substrate.

T**able S1. Amide-I peak positions and integrated intensities with corresponding band assignments of centrifuge filtered (MWCO 100 kDa) Aβ(1-42) peptides.**

| **Peptide Aβ(1-42)** | **α-helix**  **cm^−1^ (%)** | **β-sheet**  **cm^−1^ (%)** | **β-sheet**  **organization**  **index** | **Unordered helix + random**  **cm^−1^ (%)** |
| --- | --- | --- | --- | --- |
| Aβ(1-42) – HFIP protocol | 1651  (35.9 ± 4.2) | 1630 / 1693  (45.0 ± 3.6) | 0.156 ± 0.04 | 1670  (19.1 ± 7.5) |
| Aβ(1-42) – HFIP-free protocol | 1654  (21.0 ± 6.1) | 1628 / 1696  (67.5 ± 8.5) | 0.051 ± 0.04 | 1671  (11.5 ± 9.3) |

Fig S1 compares FTIR spectra in Amide-I spectral region of filtered Aβ(1-42) oligomers prepared with HFIP and HFIP-free protocol. Intensification of α-helix secondary structure for oligomers prepared by using HFIP protocol is clearly visible. In addition, the β-sheet organization index was found to be about 3 times higher for smaller oligomers (Table S1). Qualitatively the spectroscopic results are very similar prior and after centrifuge filtration with the molecular weight cut-off at 100 kDa.

Conclusion: centrifuge-filtering with the MWCO at 150 kDa does not affect secondary structure of amyloid preparations produced either with or without HFIP.
