# Supplementary material for: Amyloid β oligomers inhibit growth of human cancer cells
Source: PLoS One. 2019 Sep 11;14(9):e0221563. doi: 10.1371/journal.pone.0221563 (PMC6738617; doi:10.1371/journal.pone.0221563)
Supplement: S4 Appendix — (DOCX) [file pone.0221563.s004.docx]

Amyloid β oligomers inhibit growth of human cancer cells

**Bozena Pavliukeviciene^1^, Aiste Zentelyte^2^, Marija Jankunec^1^, Giedre Valiuliene^2^, Martynas Talaikis^1^, Ruta Navakauskiene^2^, Gediminas Niaura^1^, Gintaras Valincius^1^***

^1^Department of Bioelectrochemistry and Biospectroscopy, Institute of Biochemistry, Life Sciences Center, Vilnius University, Vilnius, Lithuania

^2^Department of Molecular Cell Biology, Institute of Biochemistry, Life Sciences Center, Vilnius University, Vilnius, Lithuania

* gintaras.valincius@gmc.vu.lt

Supporting information

S4 Appendix: Cell death in cancer cells after amyloid treatment.

The pathway of cell death induced by amyloids was analysed by flow cytometry as described in Materials and methods section.


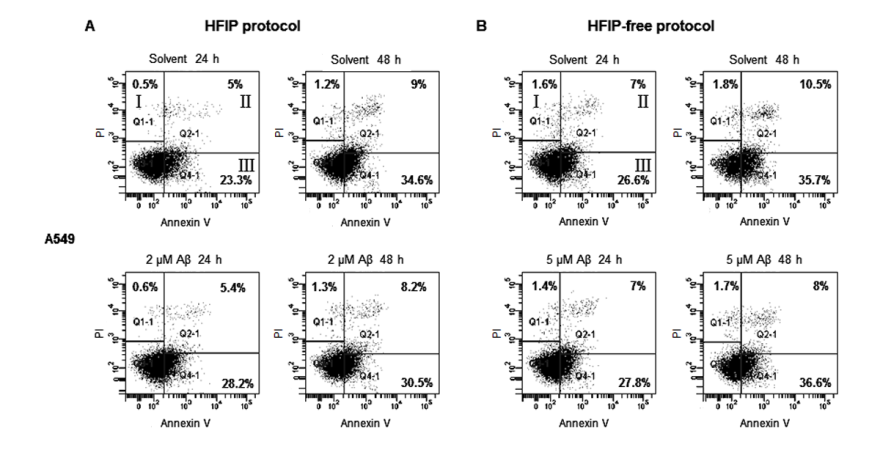


**Fig S1. Cell death analysis in A549 cancer cells.** Representative scatter plots showing Annexin V and PI staining of A549 cells, I – necrosis (Annexin V-/PI+), II – late apoptosis (Annexin V+/PI+), III – early apoptosis (Annexin V+/PI-). (A) – A549 cells treated with 2 µM of HFIP protocol amyloids and solvent for negative control. (B) – A549 cancer cells treated with 5 µM of HFIP-free protocol amyloids and solvent for negative control.


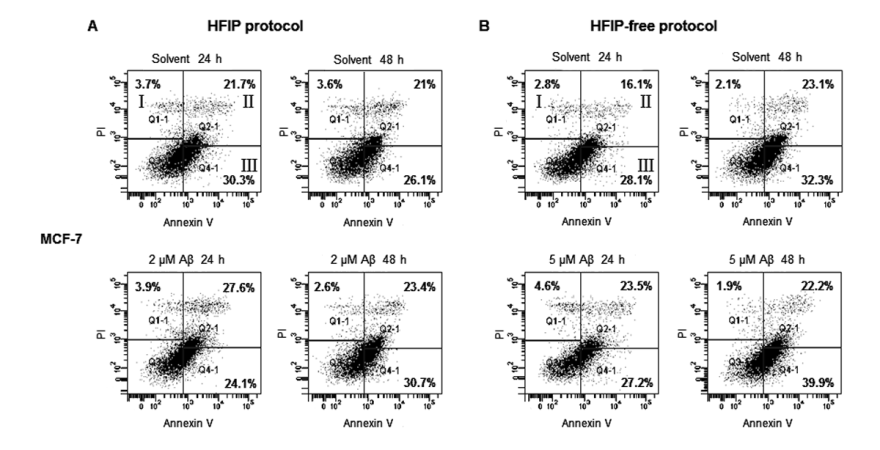


**Fig S2. Cell death analysis in MCF-7 cancer cells.** Representative scatter plots showing Annexin V and PI staining of MCF-7 cells, I – necrosis (Annexin V-/PI+), II – late apoptosis (Annexin V+/PI+), III – early apoptosis (Annexin V+/PI-). (A) – MCF-7 cells treated with 2 µM of HFIP protocol amyloids and solvent for negative control. (B) – MCF-7 cancer cells treated with 5 µM of HFIP-free protocol amyloids and solvent for negative control.
