# Supplementary material for: Amyloid β oligomers inhibit growth of human cancer cells
Source: PLoS One. 2019 Sep 11;14(9):e0221563. doi: 10.1371/journal.pone.0221563 (PMC6738617; doi:10.1371/journal.pone.0221563)
Supplement: S5 Appendix — (DOCX) [file pone.0221563.s005.docx]

Amyloid β oligomers inhibit growth of human cancer cells

**Bozena Pavliukeviciene^1^, Aiste Zentelyte^2^, Marija Jankunec^1^, Giedre Valiuliene^2^, Martynas Talaikis^1^, Ruta Navakauskiene^2^, Gediminas Niaura^1^, Gintaras Valincius^1^***

^1^Department of Bioelectrochemistry and Biospectroscopy, Institute of Biochemistry, Life Sciences Center, Vilnius University, Vilnius, Lithuania

^2^Department of Molecular Cell Biology, Institute of Biochemistry, Life Sciences Center, Vilnius University, Vilnius, Lithuania

* gintaras.valincius@gmc.vu.lt

Supporting information

S5 Appendix: Effect of centrifuge filtered amyloid preparations on cancer cell growth.

Effects of amyloid preparations on a growth of cancer cell lines NB4 and A549 were investigate using centrifuge-filtered (MWCO 100 kDa) oligomer samples. Qualitatively similar effects were observed in both centrifuge-filtered and plain preparations of amyloid oligomers. Specifically, as it follows from Fig S1, the effect of inhibition is consistently lower for A549 compared to NB4 cells. Such trend was observed for unfiltered samples as well (see the article, Fig 7). Similar effect is observed for HFIP-free preparations (Fig S2). The NB4 cells were considerably more susceptible to the inhibition by the amyloid species.


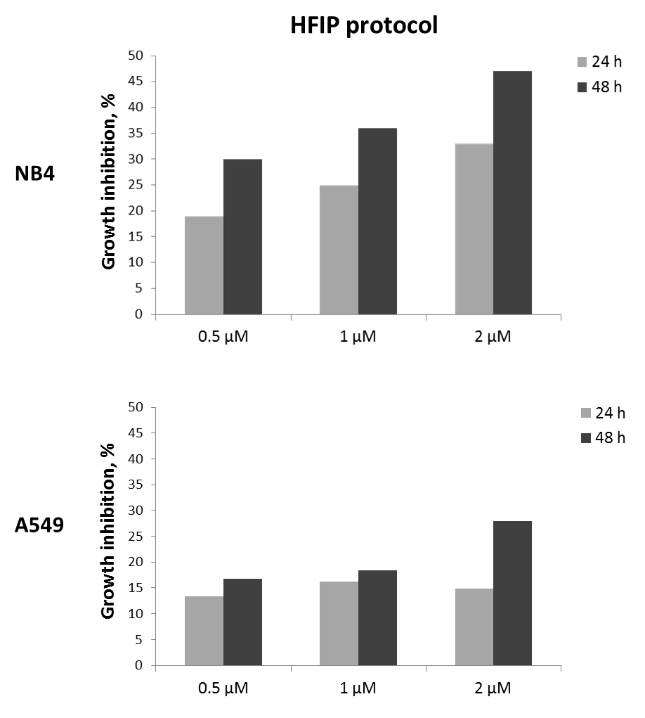


**Fig S1.** Growth inhibition of NB4 and A549 cancer cell lines treated with centrifuge-filtered amyloids. HFIP protocol amyloids, centrifuge-filtered through Amicon filters with MWCO 100 kDa, amyloid concentration range 0.5-2 µM.


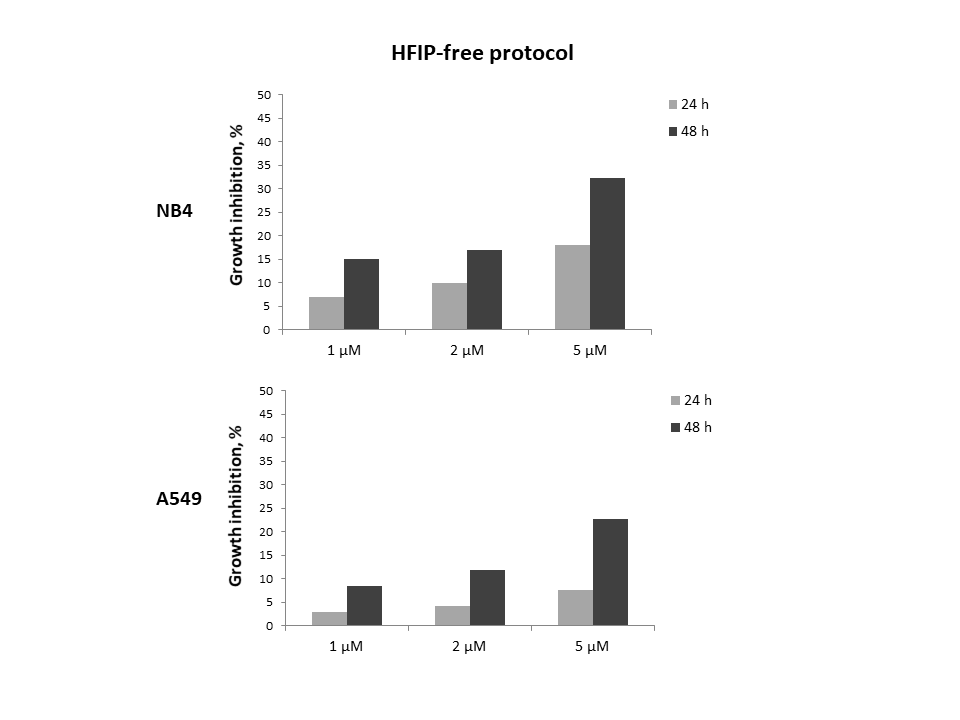


**Fig S2**. Growth inhibition of NB4 and A549 cancer cell lines treated with centrifuge-filtered amyloids. HFIP-free protocol amyloids, centrifuge-filtered through Amicon filters with MWCO 100 kDa, amyloid concentration range 1-5 µM.

Comparing the data in Figures 1S and 2S one may clearly see the difference in inhibition potential of HFIP and HFIP-free preparations. For both NB4 and A549 cell lines HFIP preparations (at the same concentration levels) exhibited considerably higher inhibition of the cell growth, which is full consistent with the data obtained with sample filtered only by 0.22 µm filters.

Conclusion: centrifuge filtering with the MWCO at 100 kDa does not affect biological function of amyloid preparations tested in the current study.
