# Supplementary material for: Amyloid β oligomers inhibit growth of human cancer cells
Source: PLoS One. 2019 Sep 11;14(9):e0221563. doi: 10.1371/journal.pone.0221563 (PMC6738617; doi:10.1371/journal.pone.0221563)
Supplement: S2 Dataset — (DOCX) [file pone.0221563.s007.docx]

1. **Data set to construct Fig. 2**

|  | HFIP protocol | HFIP-free protocol |
| --- | --- | --- |
| Height range, nm | Count | Count |
| 0,8 | 32 | 6 |
| 1 | 131 | 24 |
| 1,2 | 148 | 36 |
| 1,4 | 116 | 36 |
| 1,6 | 101 | 33 |
| 1,8 | 64 | 39 |
| 2 | 63 | 65 |
| 2,2 | 39 | 35 |
| 2,4 | 56 | 48 |
| 2,6 | 36 | 45 |
| 2,8 | 16 | 44 |
| 3 | 9 | 41 |
| 3,2 | 16 | 41 |
| 3,4 | 15 | 98 |
| 3,6 | 6 | 34 |
| 3,8 | 1 | 44 |
| 4 | 2 | 32 |
| 4,2 | 7 | 29 |
| 4,4 | 2 | 32 |
| 4,6 | 2 | 35 |
| 4,8 | 0 | 8 |
| 5 | 4 | 8 |
| 5,2 | 1 | 2 |

**Gauss distribution (curves)**

*HFIP protocol*

R^2^ = 0,848

Parameter Value StdErr CV(%) Dependencies

a 1,181e+2 1,022e+1 8,648e+0 0,3478883

b 6,218e-1 8,892e-2 1,430e+1 0,4945265

x0 1,350e+0 8,090e-2 5,991e+0 0,2672965

*HFIP-free protocol*

R^2^ = 0,522

Parameter Value StdErr CV(%) Dependencies

a 5,332e+1 5,444e+0 1,021e+1 0,3879667

b 1,357e+0 1,954e-1 1,440e+1 0,3925203

x0 2,819e+0 1,637e-1 5,806e+0 9,7196821e-3

1. **S2 Appendix. Figure S1**

Data set tp construct Figure S1. Filtered vs unfiltered through 100 kDa.

| Height range | Probabilities | |
| --- | --- | --- |
| nm | Non-filtered, % | Filtered, % |
| 0,8 | 0 | 0,1305 |
| 1 | 3,8161 | 3,7859 |
| 1,2 | 14,5707 | 10,3133 |
| 1,4 | 23,157 | 13,0548 |
| 1,6 | 17,693 | 15,9269 |
| 1,8 | 20,7285 | 17,3629 |
| 2 | 12,1422 | 13,9687 |
| 2,2 | 4,2498 | 12,2715 |
| 2,4 | 2,6886 | 8,7467 |
| 2,6 | 0,7806 | 4,047 |
| 2,8 | 0,1735 | 0,3916 |
