# Supplementary material for: Amyloid β oligomers inhibit growth of human cancer cells
Source: PLoS One. 2019 Sep 11;14(9):e0221563. doi: 10.1371/journal.pone.0221563 (PMC6738617; doi:10.1371/journal.pone.0221563)
Supplement: S4 Dataset — (DOCX) [file pone.0221563.s009.docx]

1. **Data set to construct Fig 7**

| **NB4 cells** | | | | | |
| --- | --- | --- | --- | --- | --- |
| HFIP protocol | | | HFIP-free protocol | | |
| GI, % | 24h | 48h | GI, % | 24h | 48h |
| 0,5 µM | 8,760±3,41 | 3,658±2,32 | 1 µM | 2,150±4,8 | 15,492±4,6 |
| 1 µM | 8,390±2,68 | 26±6,57 | 2 µM | 12,676±5,2 | 18,823±4,8 |
| 2 µM | 22,320±6,35 | 35,051±3,1 | 5 µM | 12,676±5,1 | 20,430±5,2 |
| **A549 cells** | | | | | |
| HFIP protocol | | | HFIP-free protocol | | |
| GI, % | 24h | 48h | GI, % | 24h | 48h |
| 0,5 µM | 5,985±4,66 | 13,015±3,66 | 1 µM | 2,182±0,92 | 11,994±1,87 |
| 1 µM | 7,847±5,08 | 19,781±4,08 | 2 µM | 8,402±9,17 | 19,981±4,71 |
| 2 µM | 15,277±10,38 | 21,827±6,38 | 5 µM | 17,074±5,06 | 36,339±4,1 |
| **MCF-7 cells** | | | | | |
| HFIP protocol | | | HFIP-free protocol | | |
| GI, % | 24h | 48h | GI, % | 24h | 48h |
| 0,5 µM | 7,181±5,86 | 5±3,86 | 1 µM | 3,067±4,02 | 8,545±10,96 |
| 1 µM | 8,819±7,2 | 22,581±4,2 | 2 µM | 12,898±4,19 | 15,32±7,48 |
| 2 µM | 19,762±8,95 | 32,786±5,95 | 5 µM | 17,787±8,01 | 19,11±3,93 |

1. **Data set to construct Fig 8**

| **NB4 cells** | | | | | | | |
| --- | --- | --- | --- | --- | --- | --- | --- |
| HFIP protocol | | | | HFIP-free protocol | | | |
| % of cells | G0/G1 | S | G2/M | % of cells | G0/G1 | S | G2/M |
| Solvent 24h | 39,6 | 28,9 | 31,4 | Solvent 24h | 54,6 | 27,8 | 17,6 |
| Solvent 48h | 53,9 | 22,6 | 23,4 | Solvent 48h | 46,8 | 29,1 | 24,1 |
| 1 µM 24h | 40,3 | 30,1 | 29,6 | 2 µM 24h | 59,4 | 25,4 | 15,2 |
| 1 µM 48h | 56,7 | 20,4 | 22,9 | 2 µM 48h | 54,2 | 28,2 | 17,6 |
|  |  |  |  | 5 µM 24h | 55,7 | 26,6 | 17,7 |
|  |  |  |  | 5 µM 48h | 51,6 | 28,8 | 19,6 |
| **A549 cells** | | | | | | | |
| HFIP protocol | | | | HFIP-free protocol | | | |
| % of cells | G0/G1 | S | G2/M | % of cells | G0/G1 | S | G2/M |
| Solvent 24h | 70,6 | 7,9 | 21,5 | Solvent 24h | 58,2 | 16 | 25,8 |
| Solvent 48h | 77,35 | 5,95 | 16,65 | Solvent 48h | 65,6 | 7,5 | 26,9 |
| 1 µM 24h | 67,8 | 11,8 | 20,3 | 2 µM 24h | 59,1 | 14,9 | 26 |
| 1 µM 48h | 75,3 | 6,9 | 17,75 | 2 µM 48h | 63,5 | 8,5 | 28 |
|  |  |  |  | 5 µM 24h | 59,4 | 14,75 | 25,85 |
|  |  |  |  | 5 µM 48h | 61,25 | 9,75 | 29 |
| **MCF-7 cells** | | | | | | | |
| HFIP protocol | | | | HFIP-free protocol | | | |
| % of cells | G0/G1 | S | G2/M | % of cells | G0/G1 | S | G2/M |
| Solvent 24h | 65,1 | 5,5 | 29,4 | Solvent 24h | 47,9 | 12 | 40,1 |
| Solvent 48h | 63,1 | 8,9 | 28 | Solvent 48h | 45,6 | 12,2 | 42,2 |
| 1 µM 24h | 56,3 | 7,1 | 36,6 | 2 µM 24h | 48,8 | 12,9 | 38,3 |
| 1 µM 48h | 65,05 | 6,7 | 28,25 | 2 µM 48h | 43,3 | 13,7 | 43 |
|  |  |  |  | 5 µM 24h | 49,1 | 12,7 | 38,2 |
|  |  |  |  | 5 µM 48h | 45,1 | 12,7 | 42,2 |

1. **Data set to construct S5 Appendix Fig S1 and Fig S2**

| **NB4 cells** | | | | | |
| --- | --- | --- | --- | --- | --- |
| HFIP protocol | | | HFIP-free protocol | | |
| GI, % | 24h | 48h | GI, % | 24h | 48h |
| 0,5 µM | 19 | 30 | 1 µM | 6,977 | 15,06 |
| 1 µM | 25 | 36 | 2 µM | 10 | 17 |
| 2 µM | 33 | 47 | 5 µM | 18 | 32,39 |
| **A549 cells** | | | | | |
| HFIP protocol | | | HFIP-free protocol | | |
| GI, % | 24h | 48h | GI, % | 24h | 48h |
| 0,5 µM | 9,485 | 12,578 | 1 µM | 3,002 | 8,345 |
| 1 µM | 13,153 | 17,015 | 2 µM | 4,139 | 11,946 |
| 2 µM | 17,064 | 29,162 | 5 µM | 7,552 | 22,75 |
