# Supplementary material for: Amyloid β oligomers inhibit growth of human cancer cells
Source: PLoS One. 2019 Sep 11;14(9):e0221563. doi: 10.1371/journal.pone.0221563 (PMC6738617; doi:10.1371/journal.pone.0221563)
Supplement: S5 Dataset — (PPTX) [file pone.0221563.s010.pptx]

## Slide 1
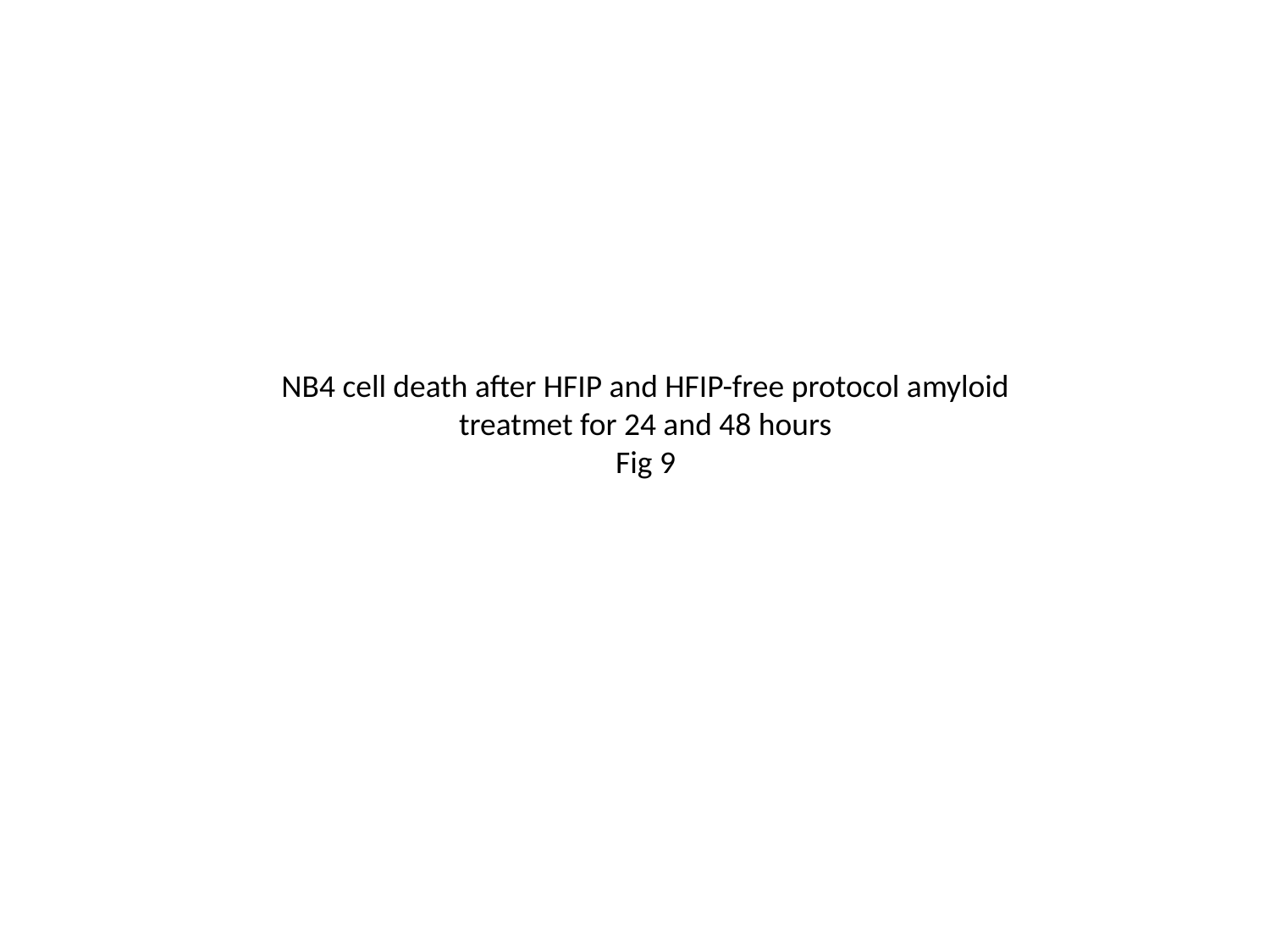

NB4 cell death after HFIP and HFIP-free protocol amyloid treatmet for 24 and 48 hours
Fig 9

## Slide 2
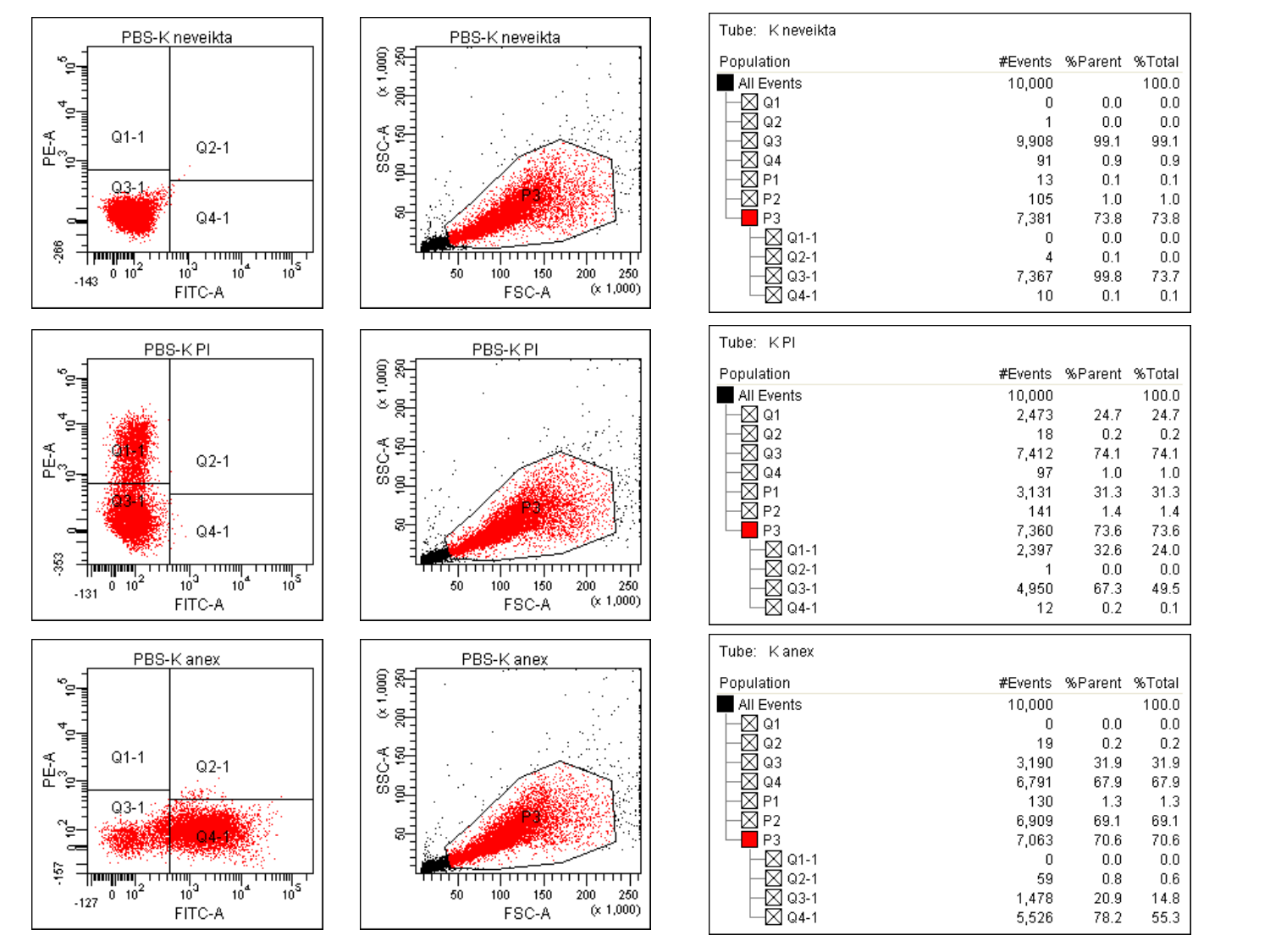

## Slide 3
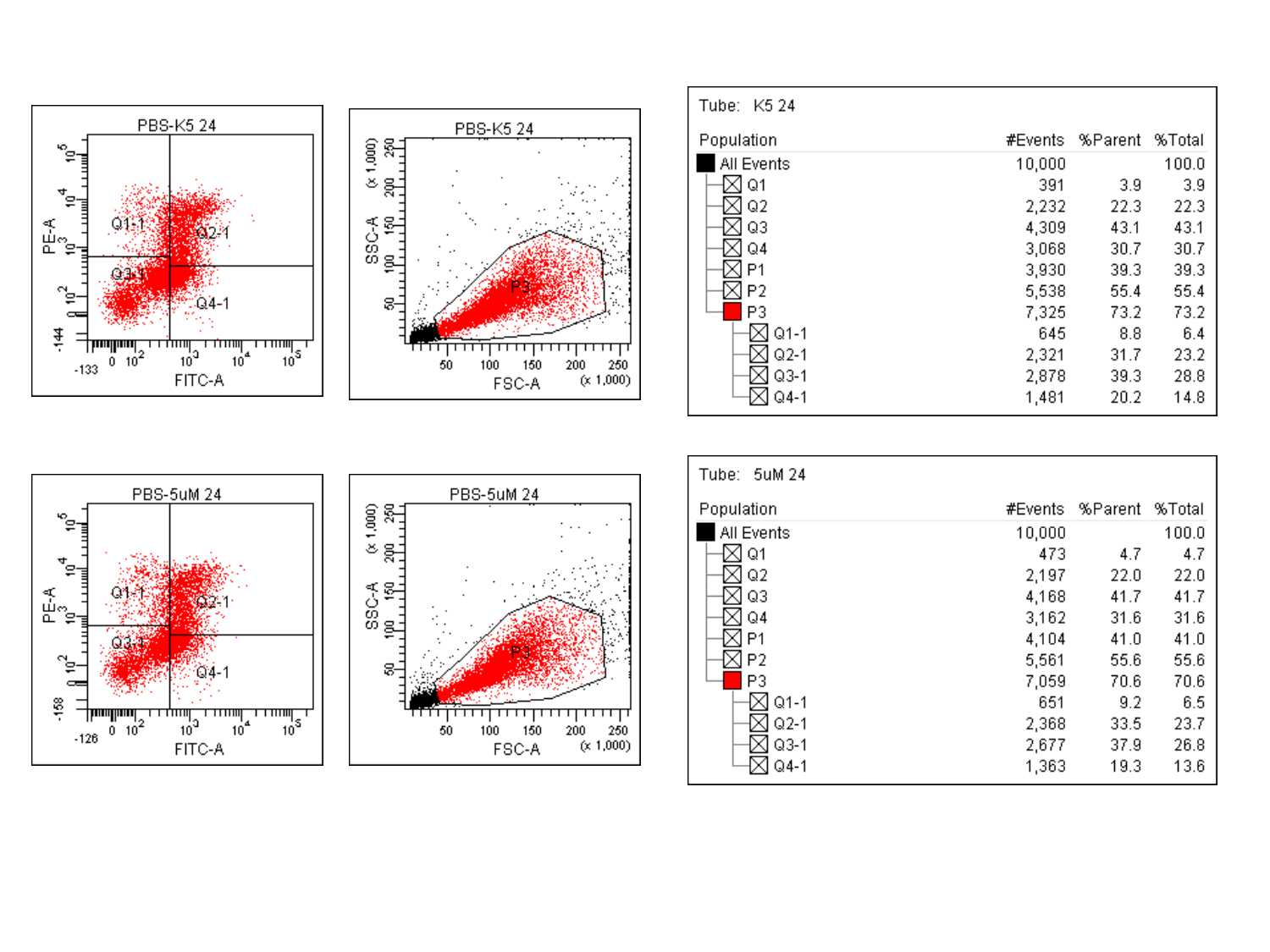

## Slide 4
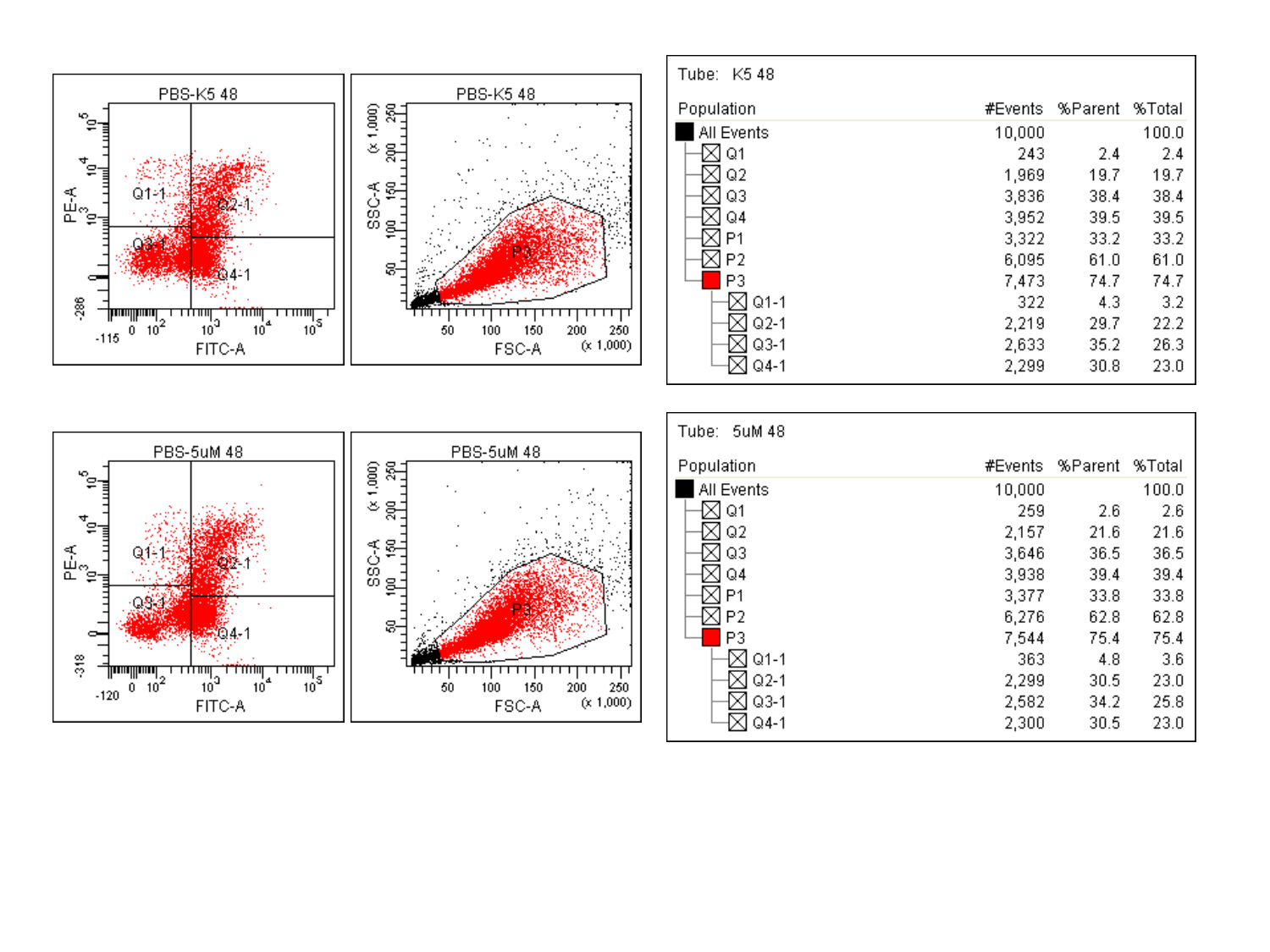

## Slide 5
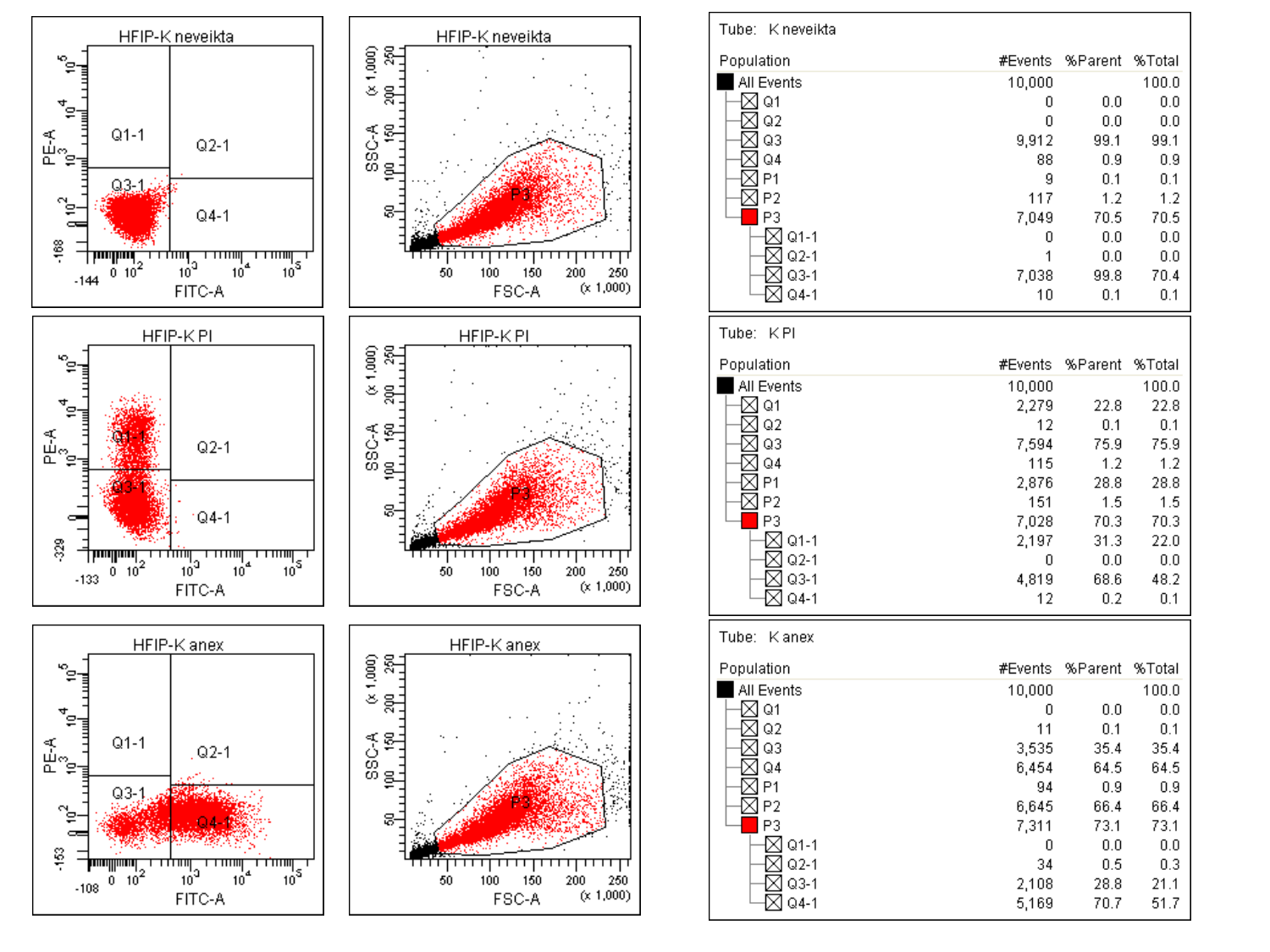

## Slide 6
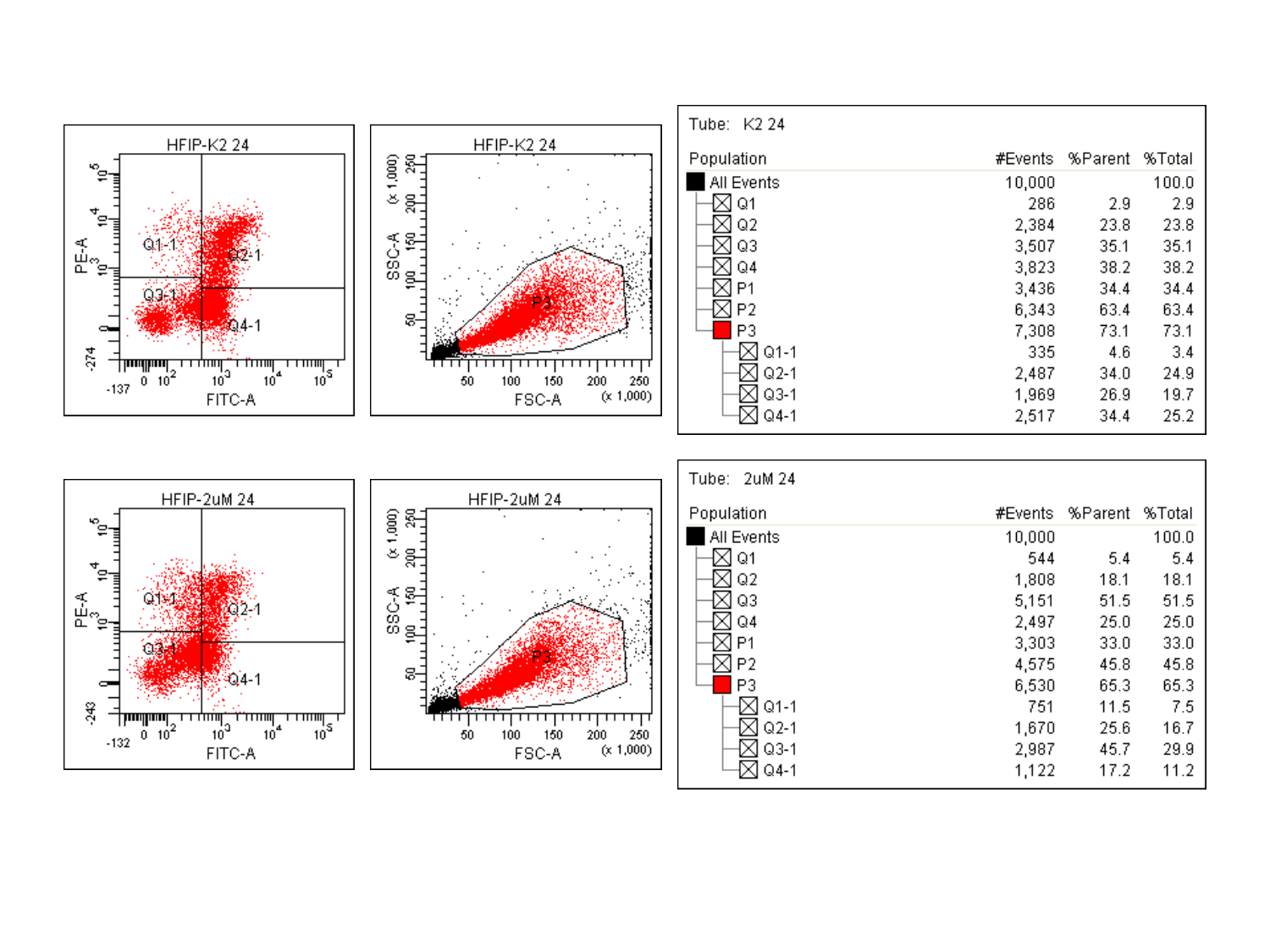

## Slide 7
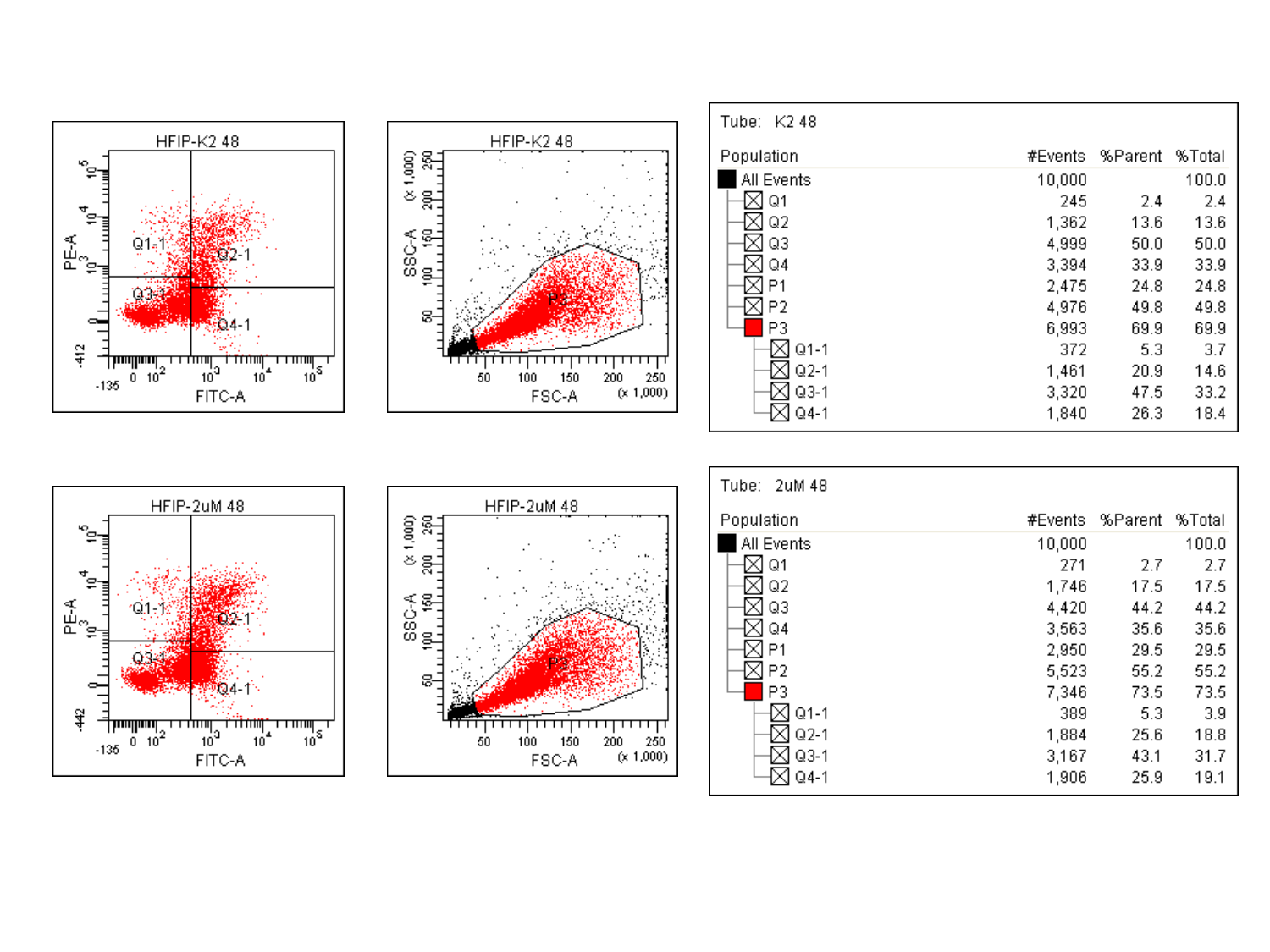

## Slide 8
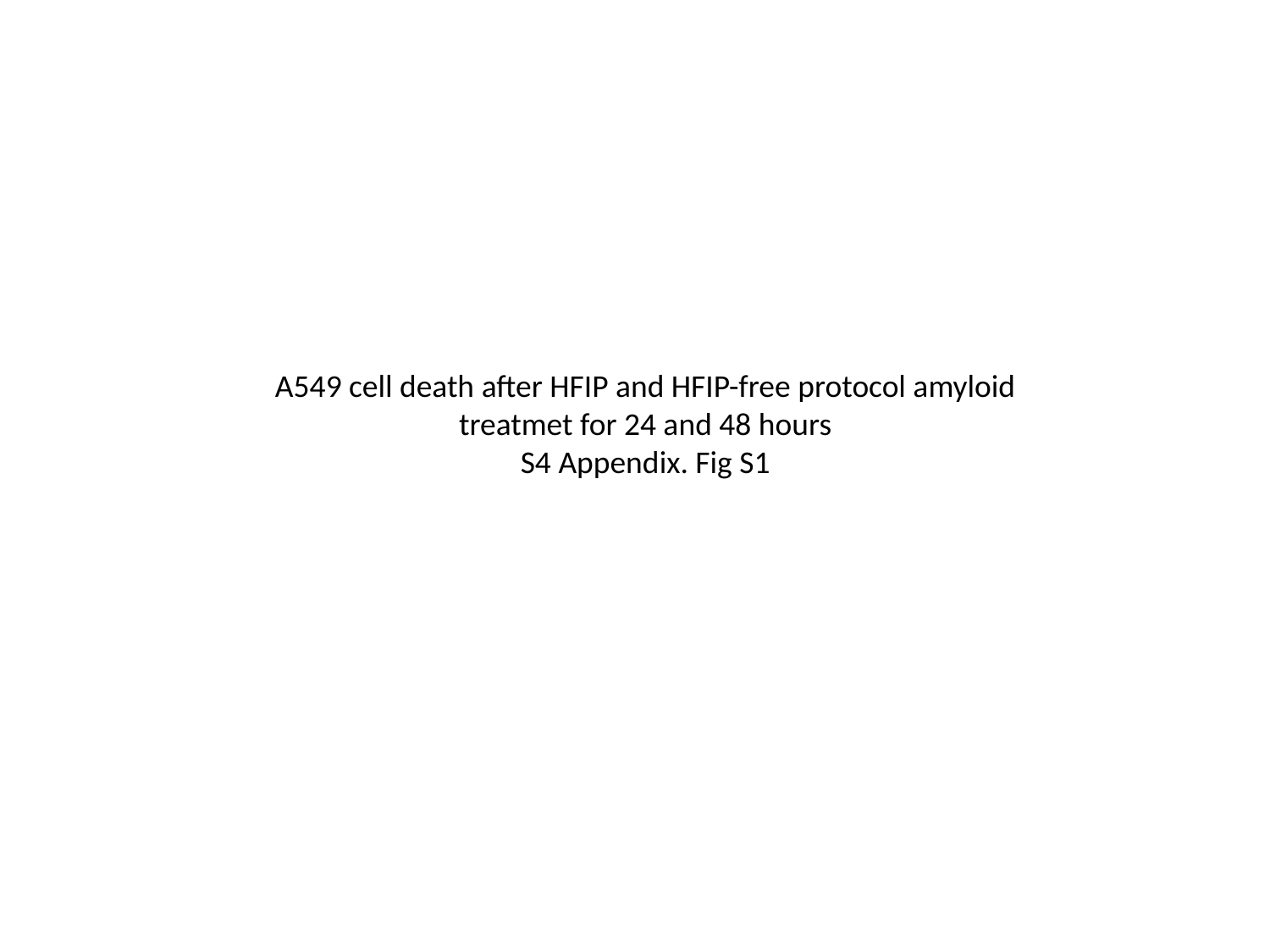

A549 cell death after HFIP and HFIP-free protocol amyloid treatmet for 24 and 48 hours
S4 Appendix. Fig S1

## Slide 9
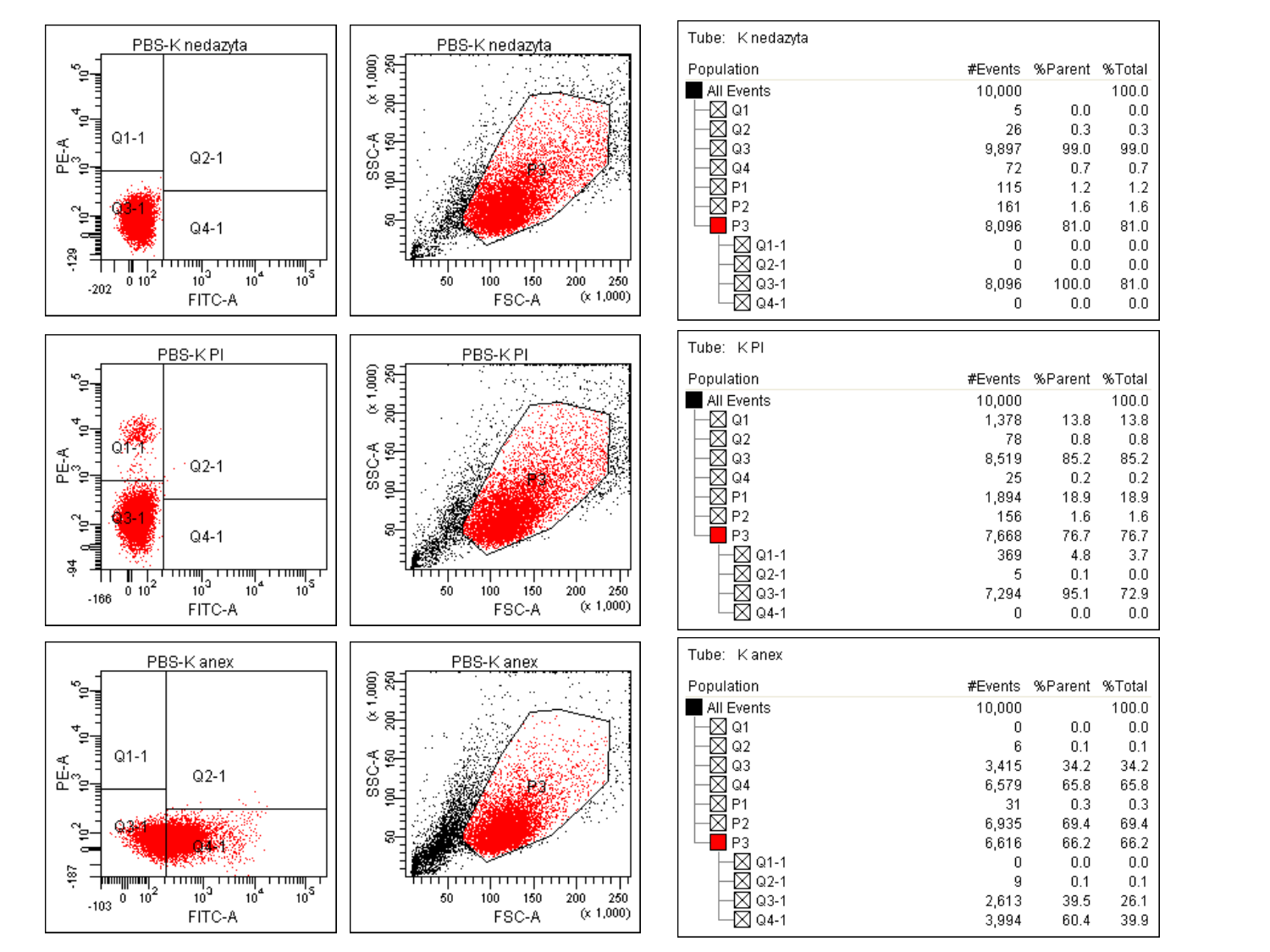

## Slide 10
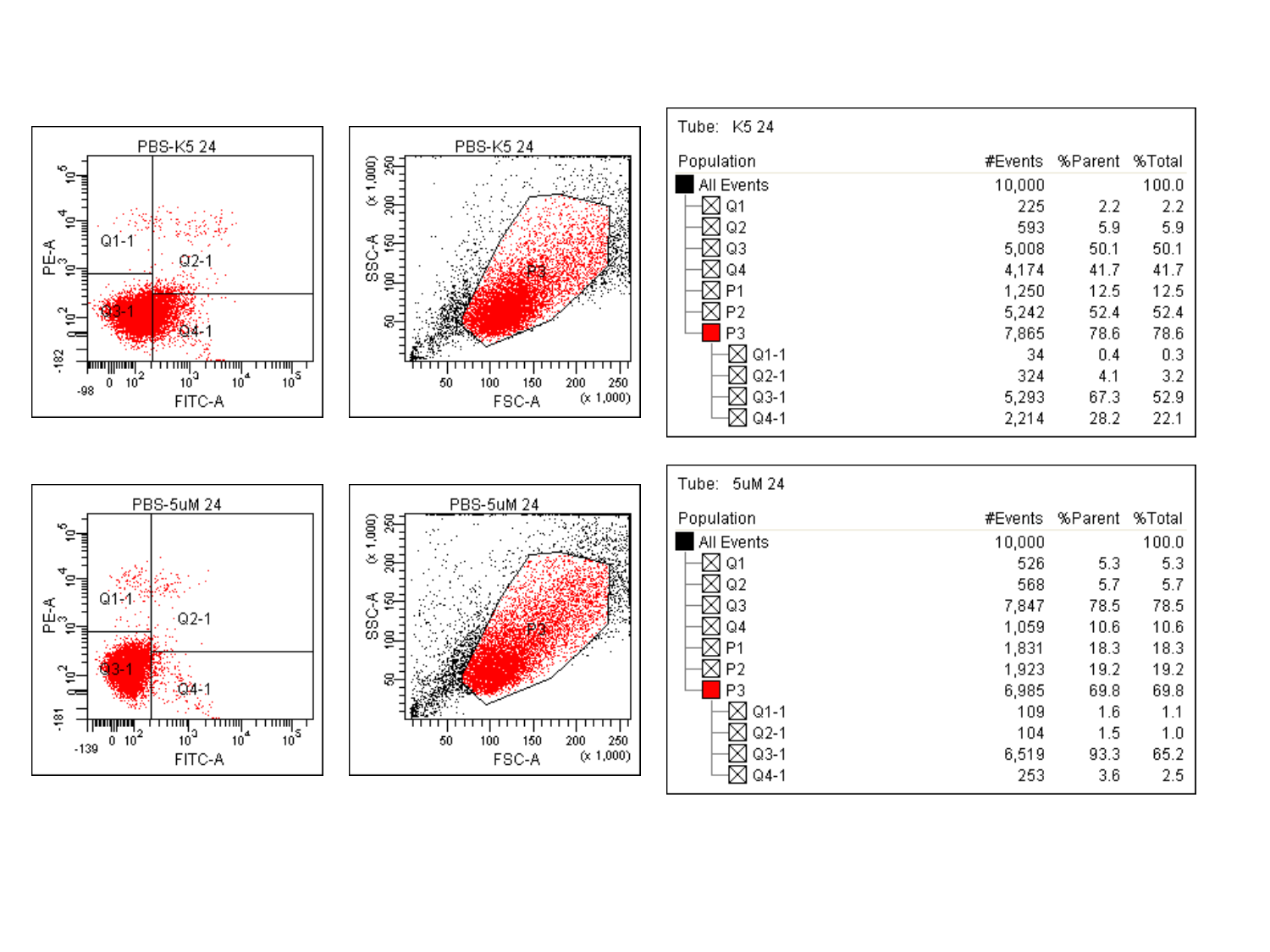

## Slide 11
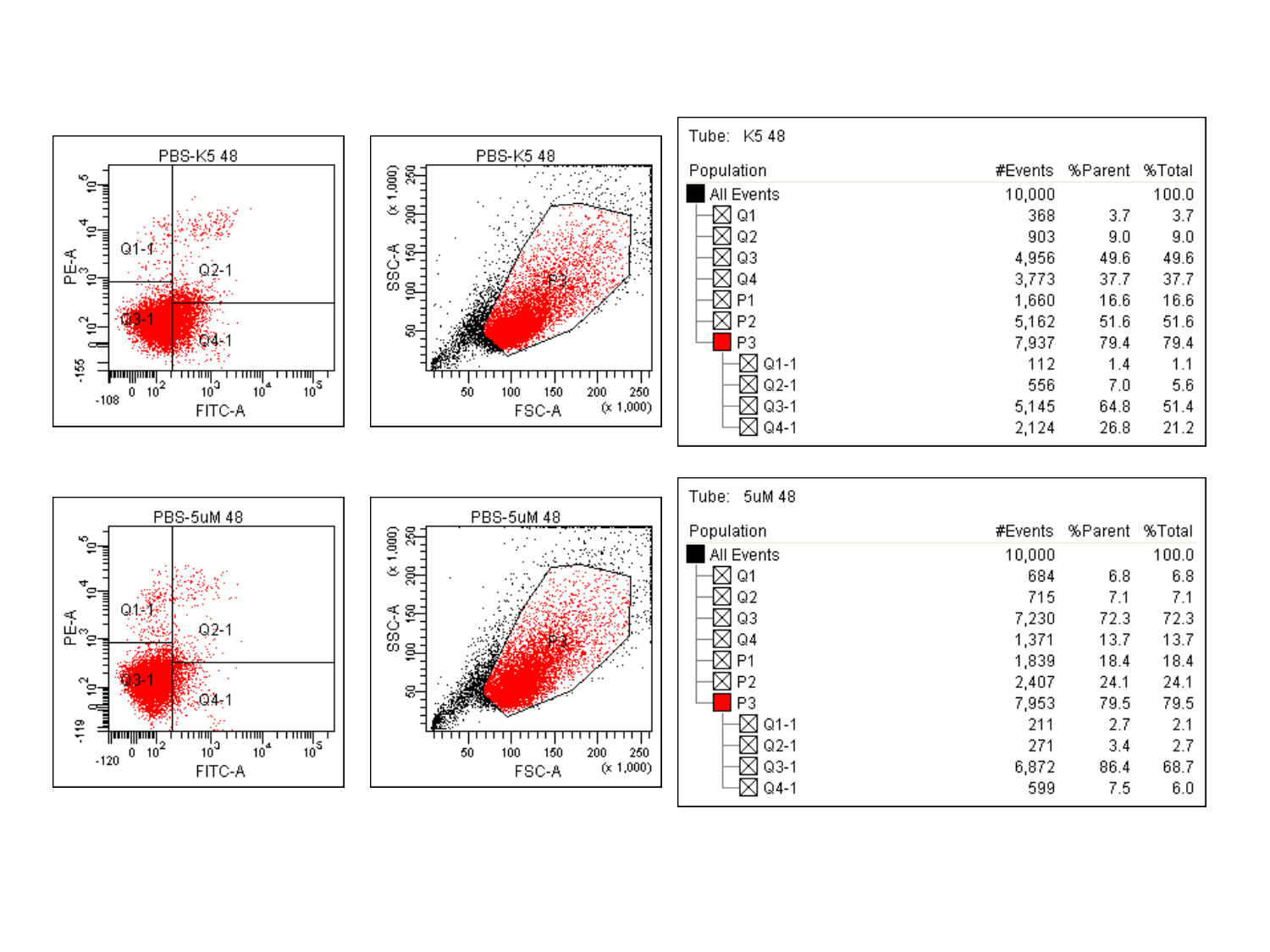

## Slide 12
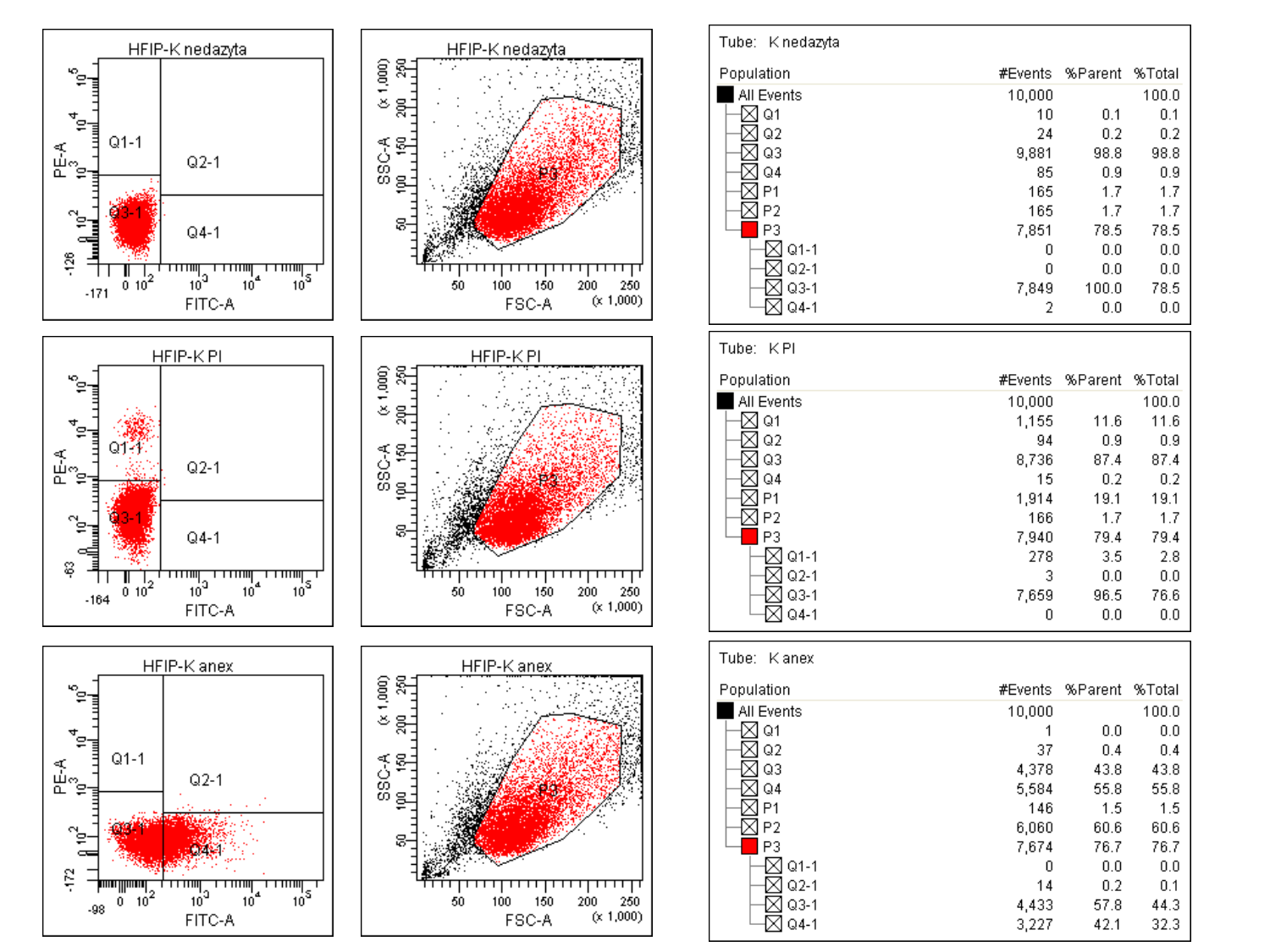

## Slide 13
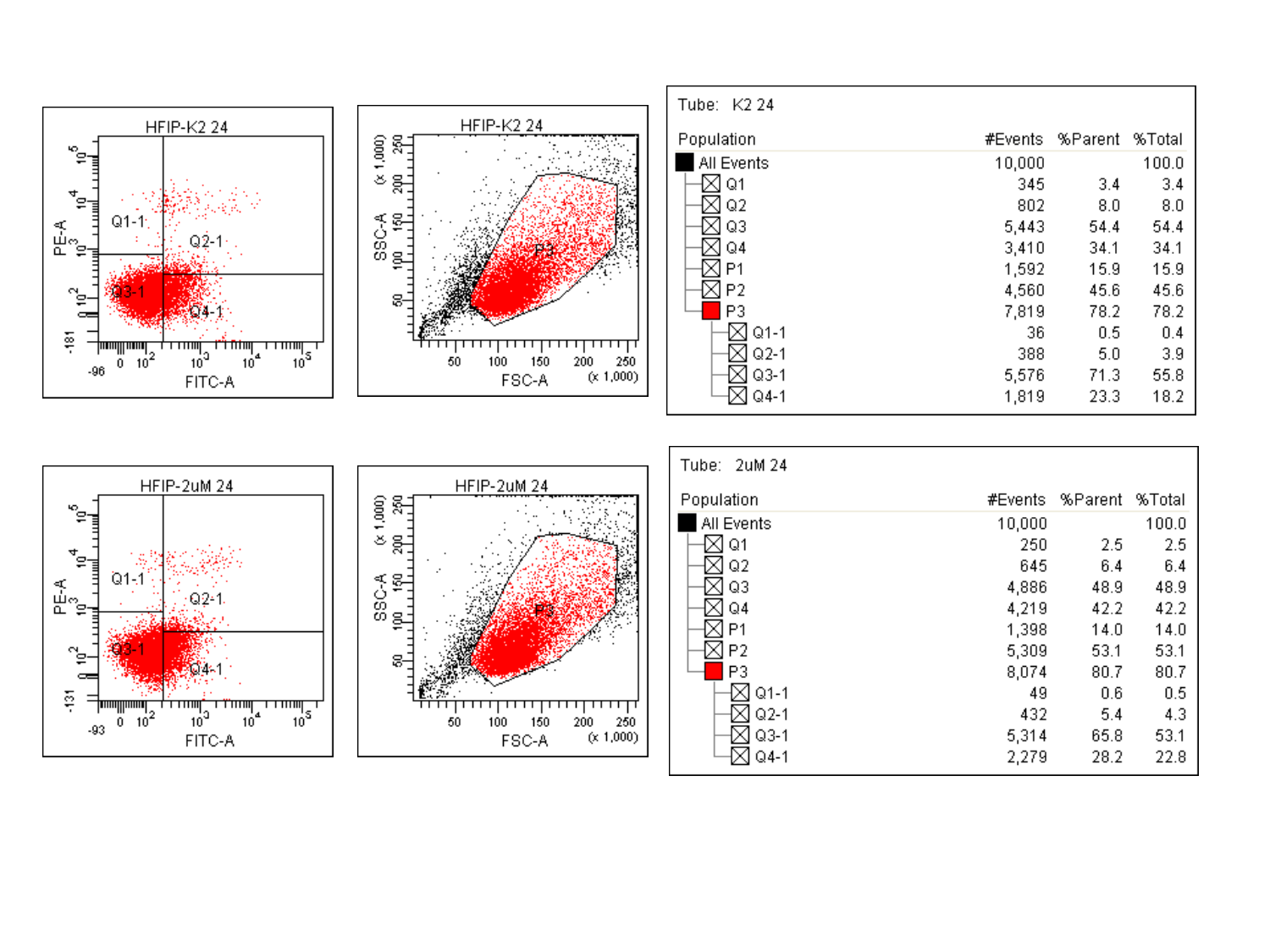

## Slide 14
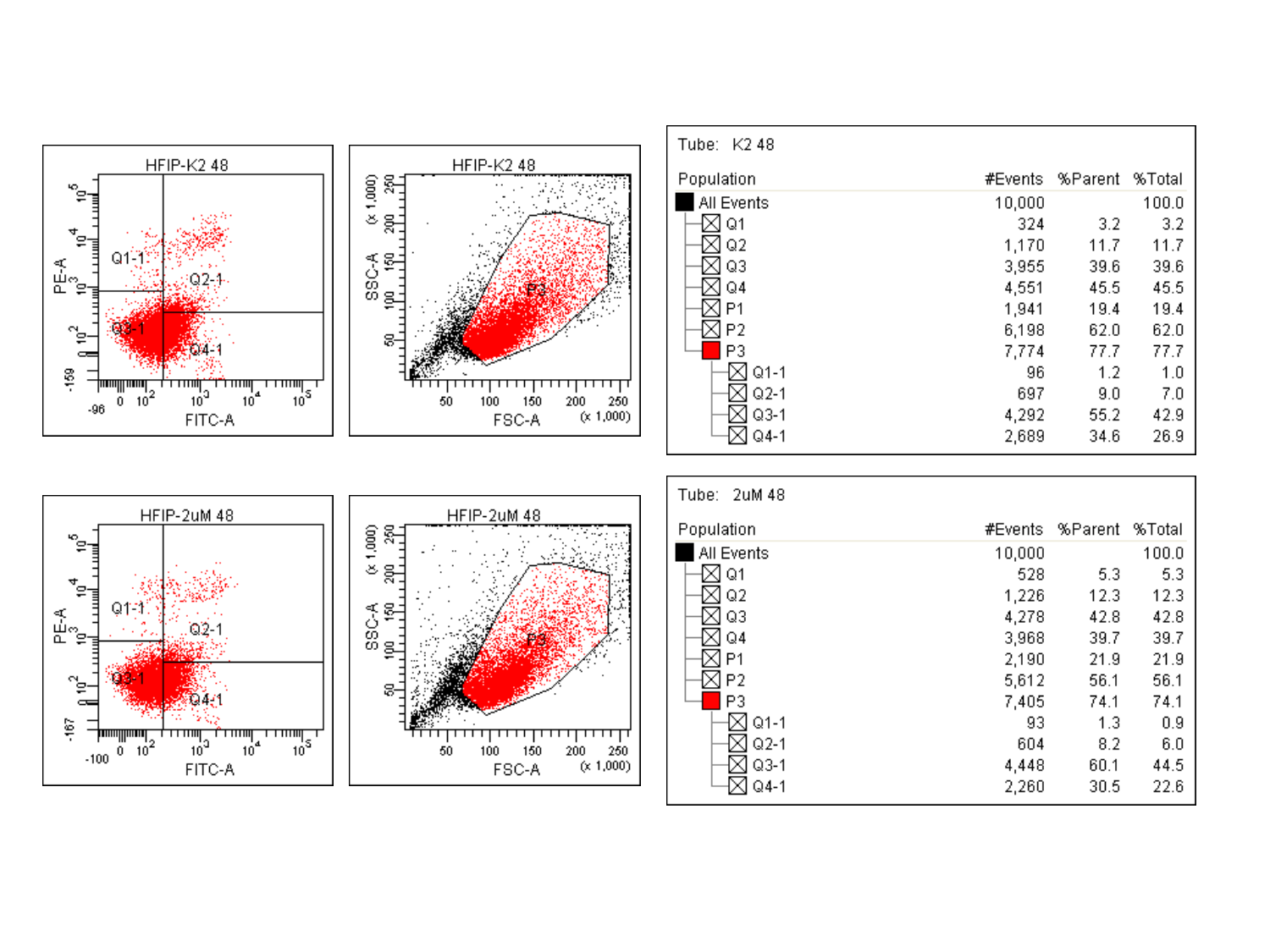

## Slide 15
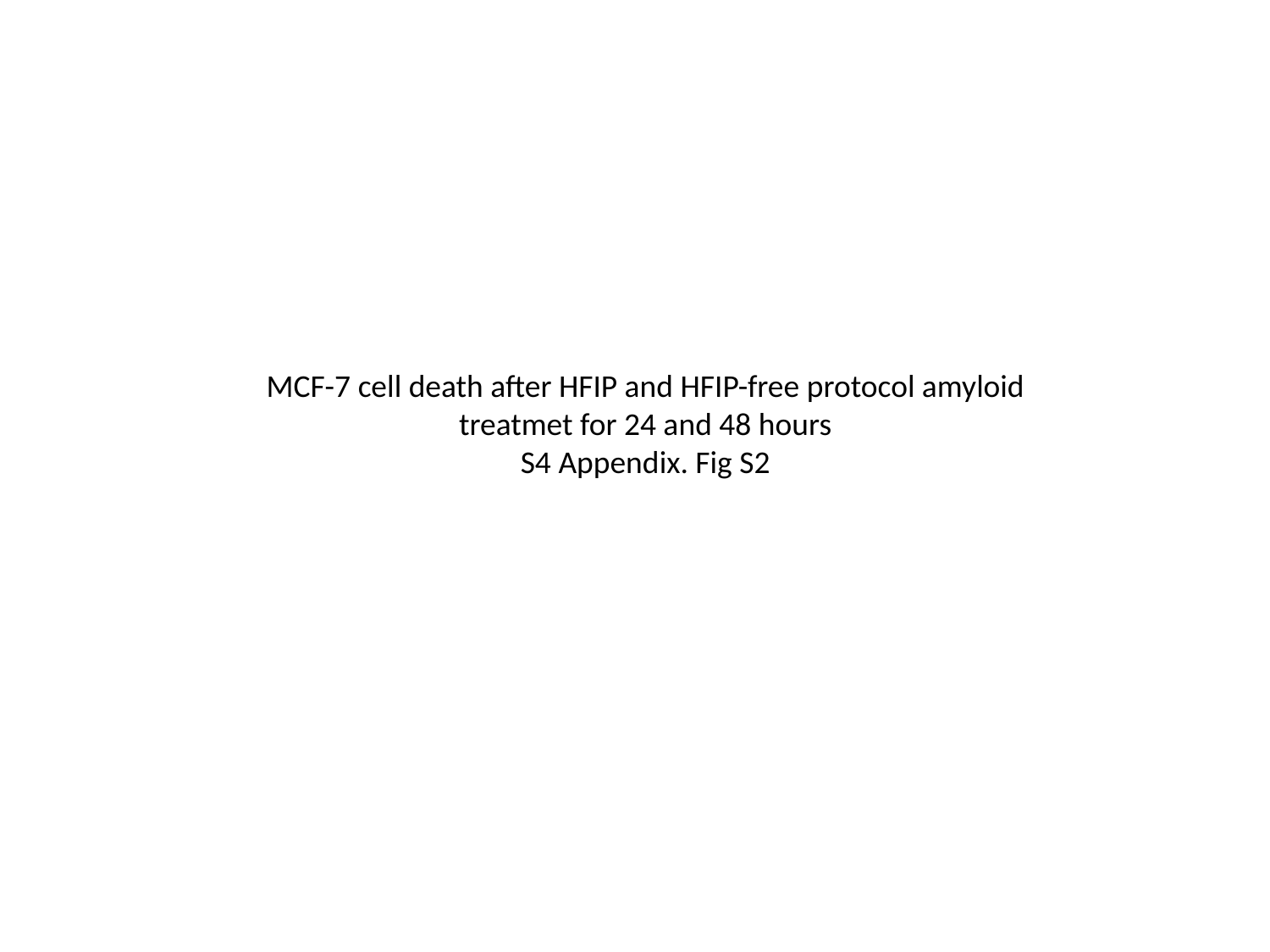

MCF-7 cell death after HFIP and HFIP-free protocol amyloid treatmet for 24 and 48 hours
S4 Appendix. Fig S2

## Slide 16
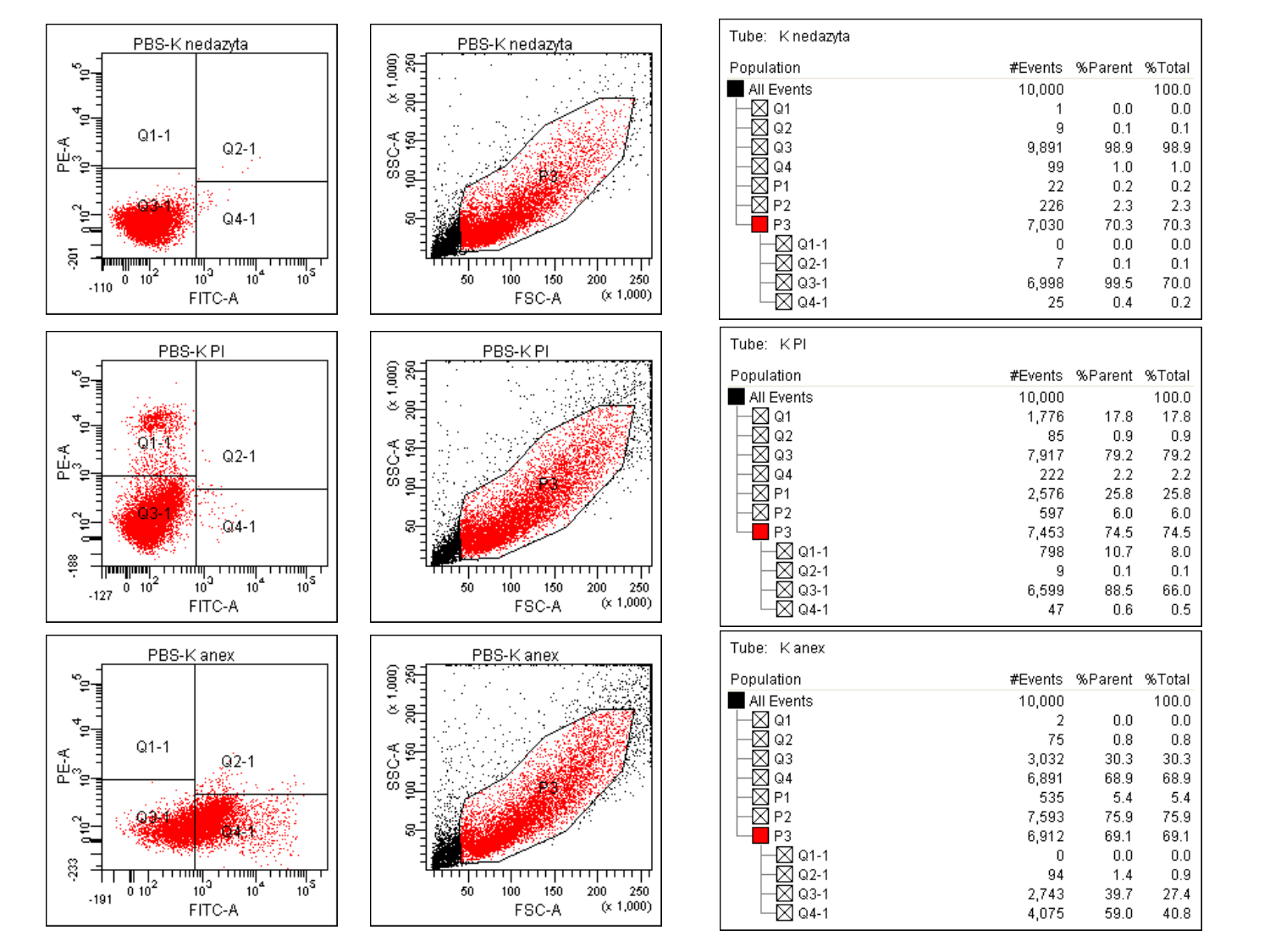

## Slide 17
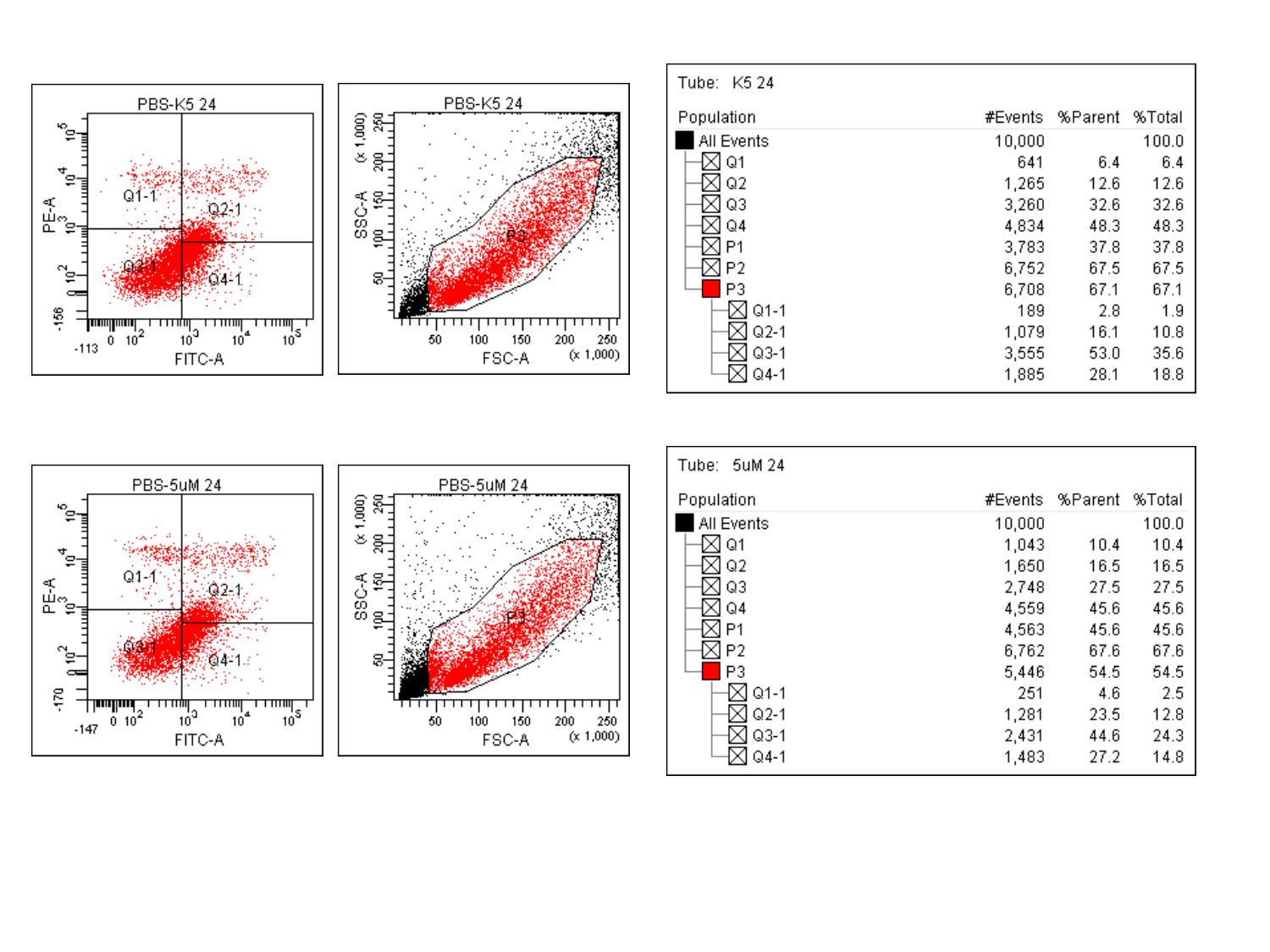

## Slide 18
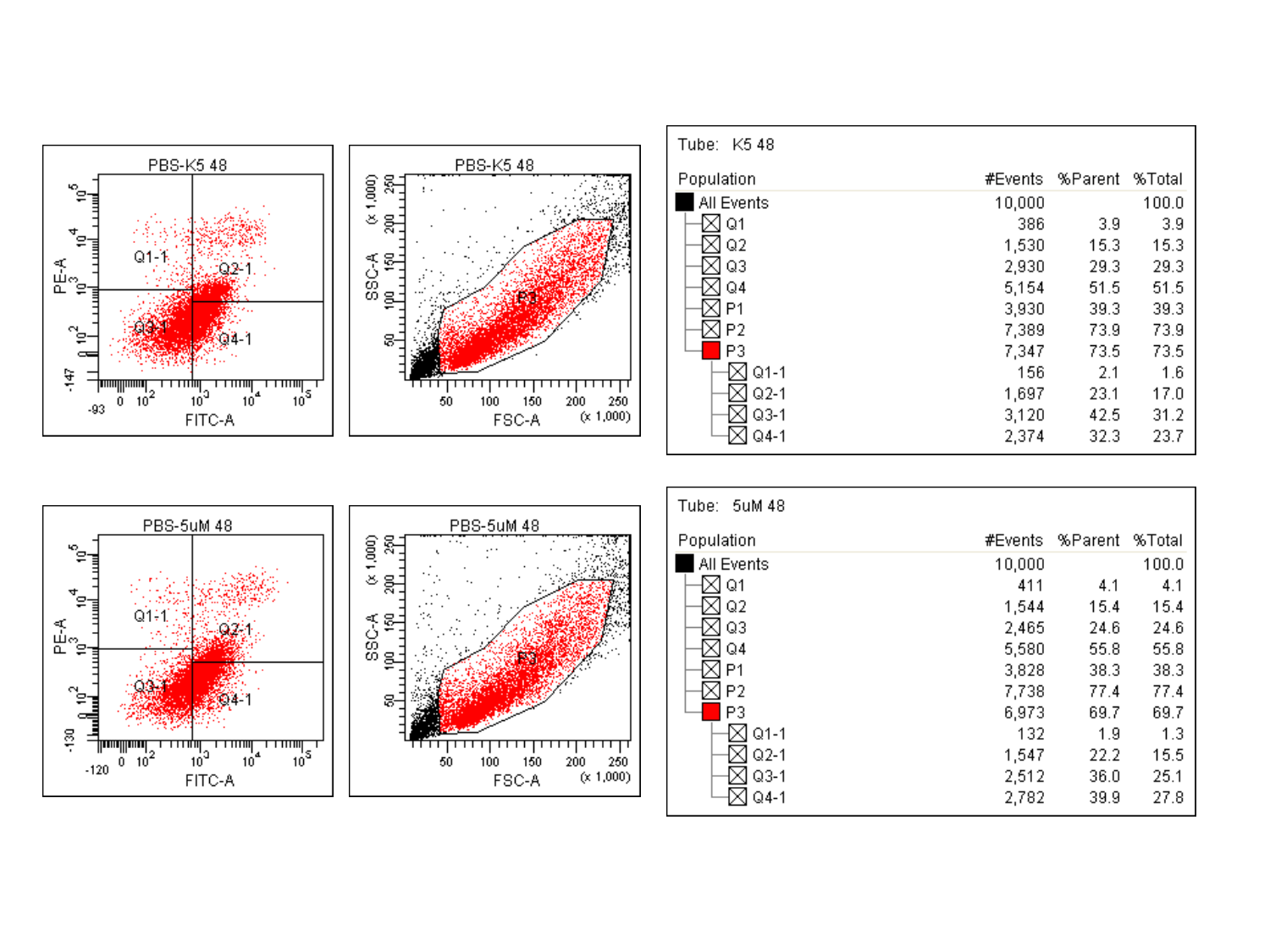

## Slide 19
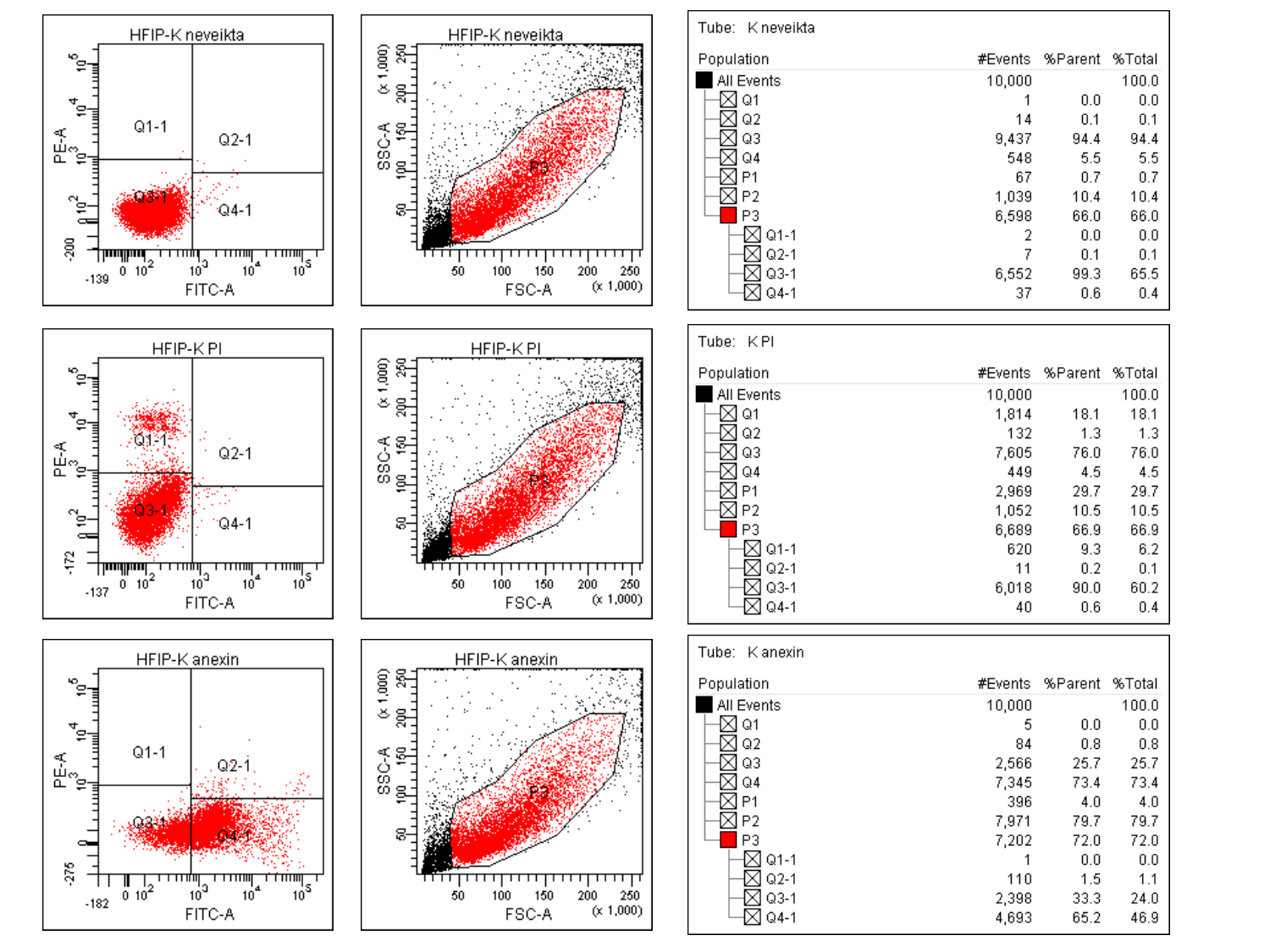

## Slide 20
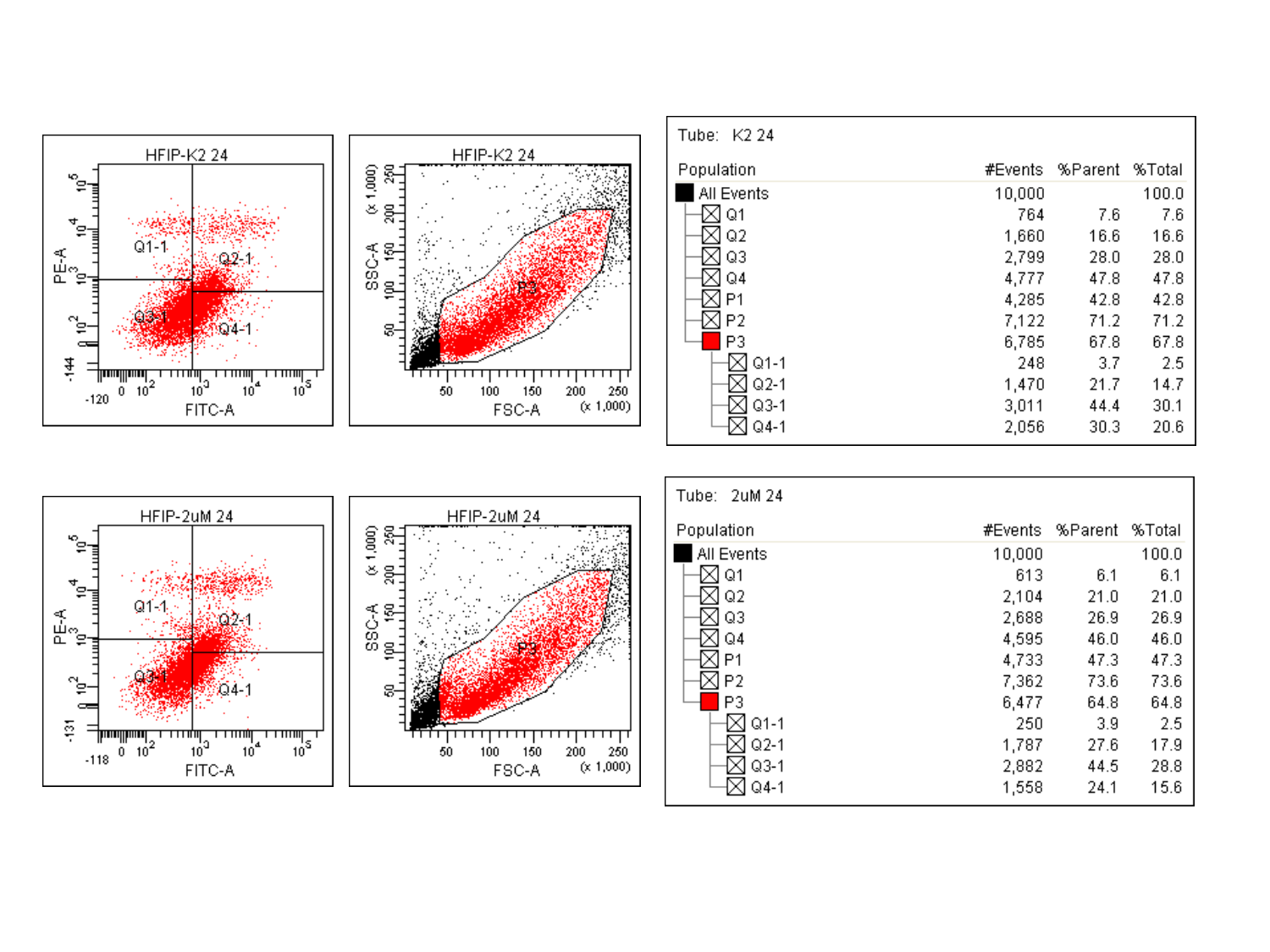

## Slide 21
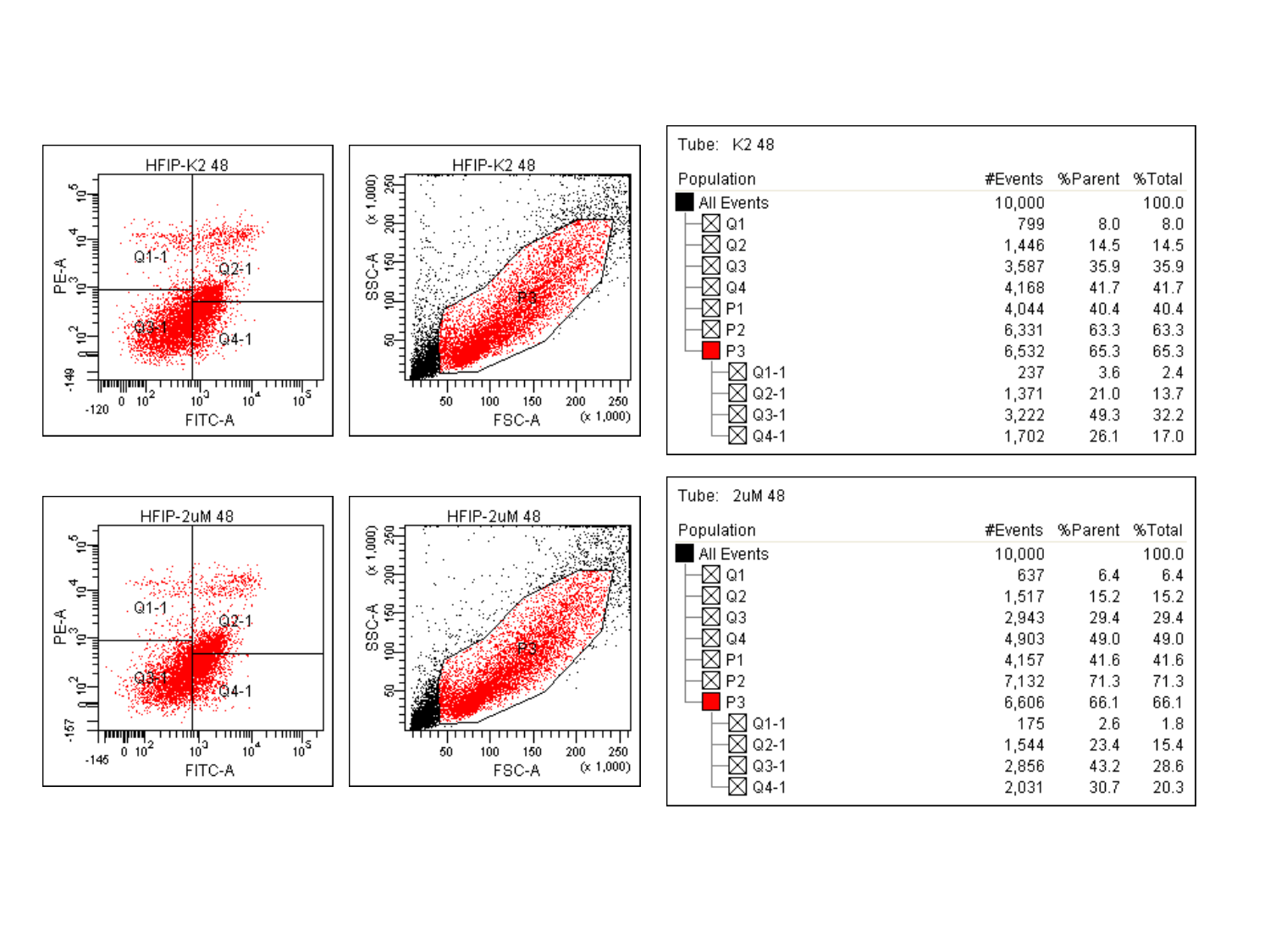

## Slide 22
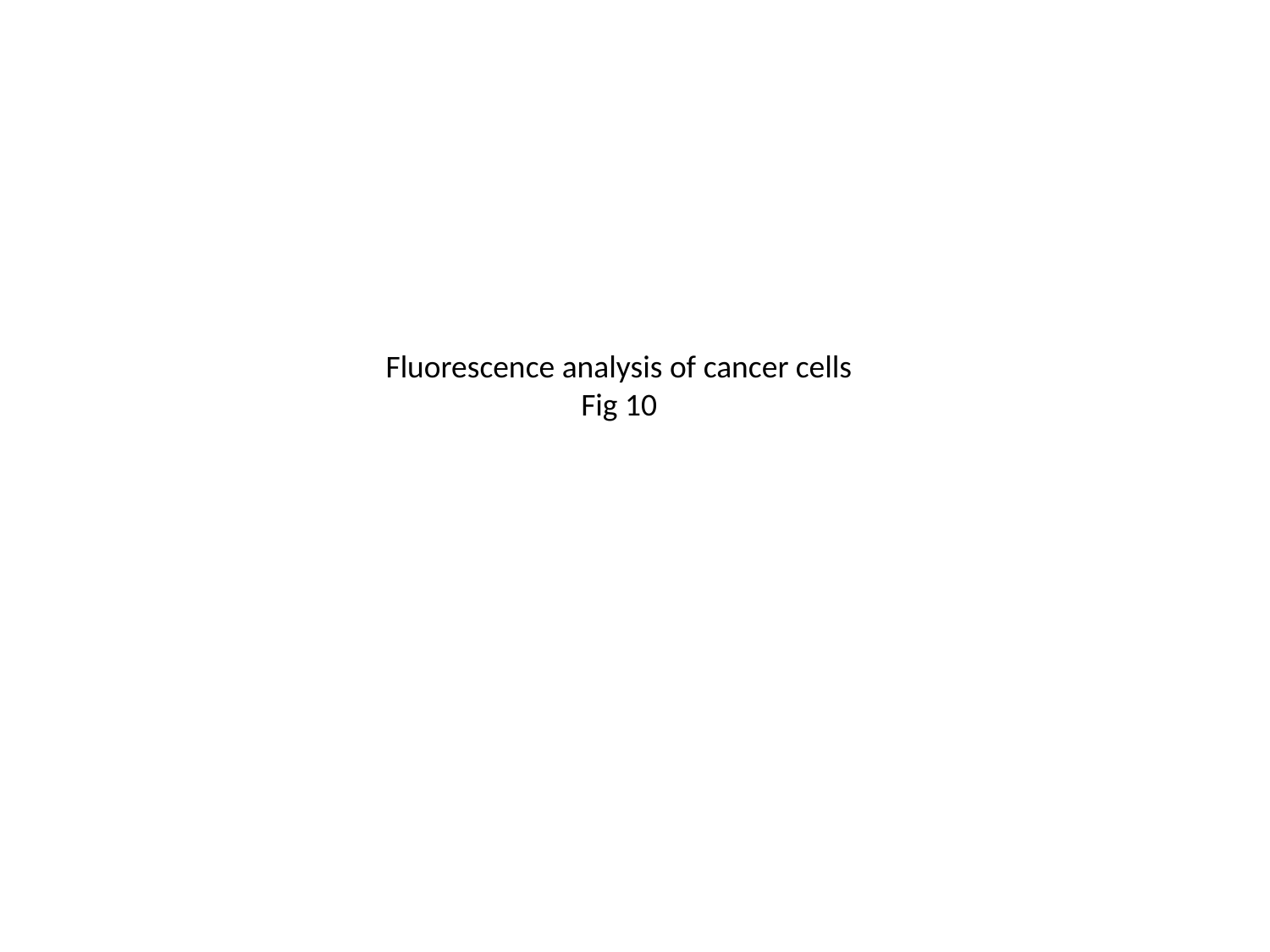

Fluorescence analysis of cancer cells
Fig 10

## Slide 23
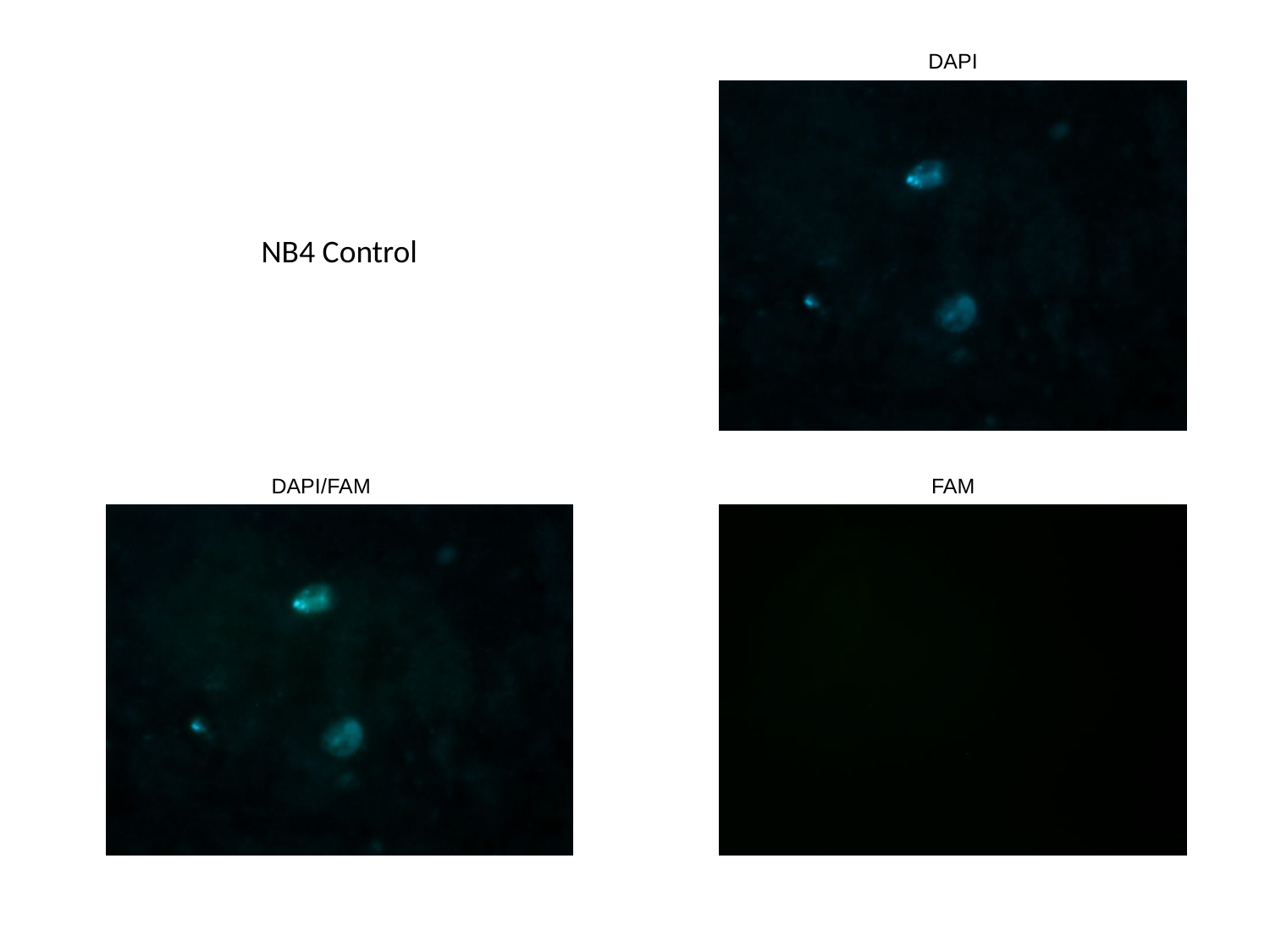

DAPI
NB4 Control
FAM
DAPI/FAM

## Slide 24
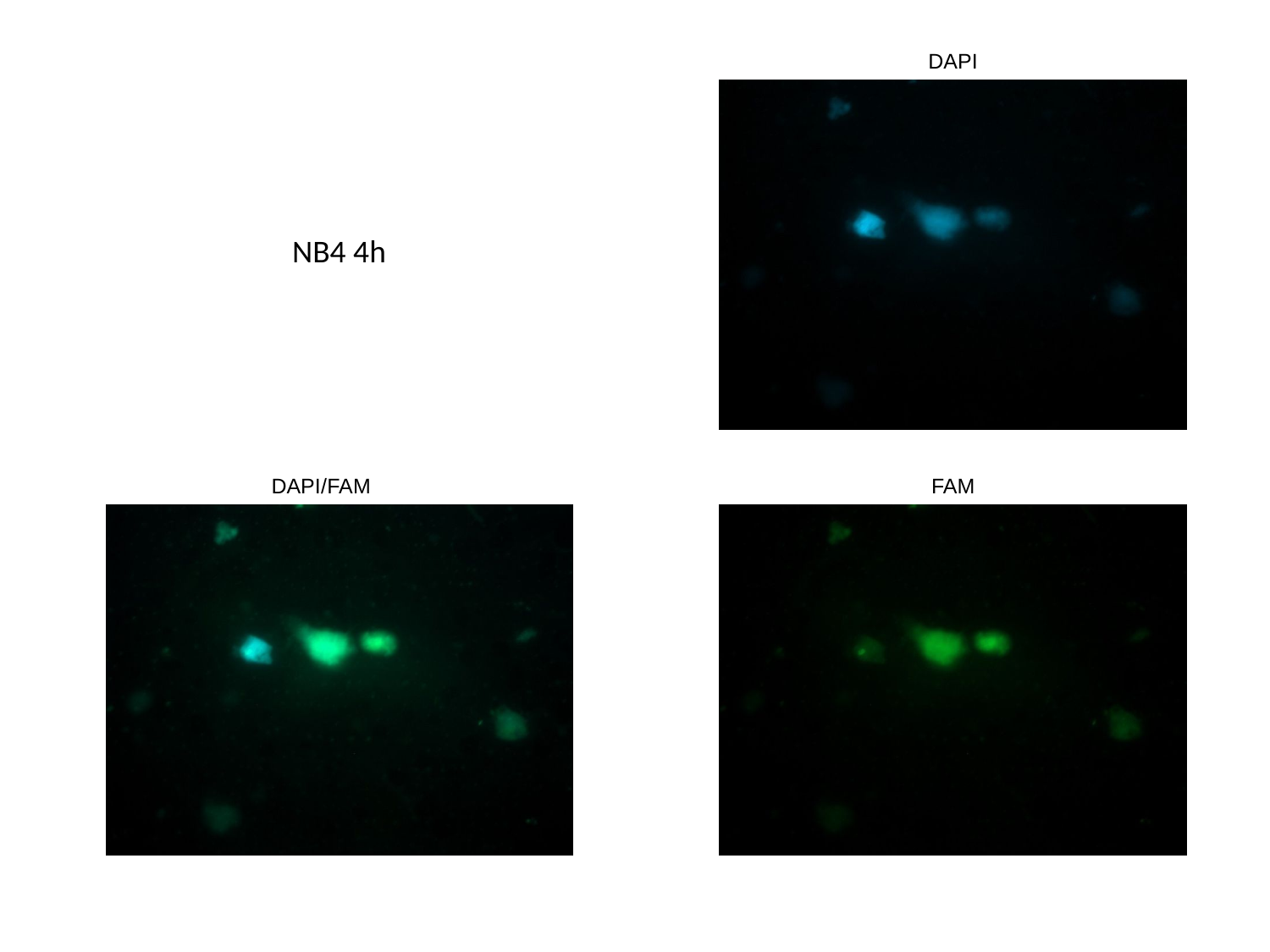

DAPI
NB4 4h
FAM
DAPI/FAM

## Slide 25
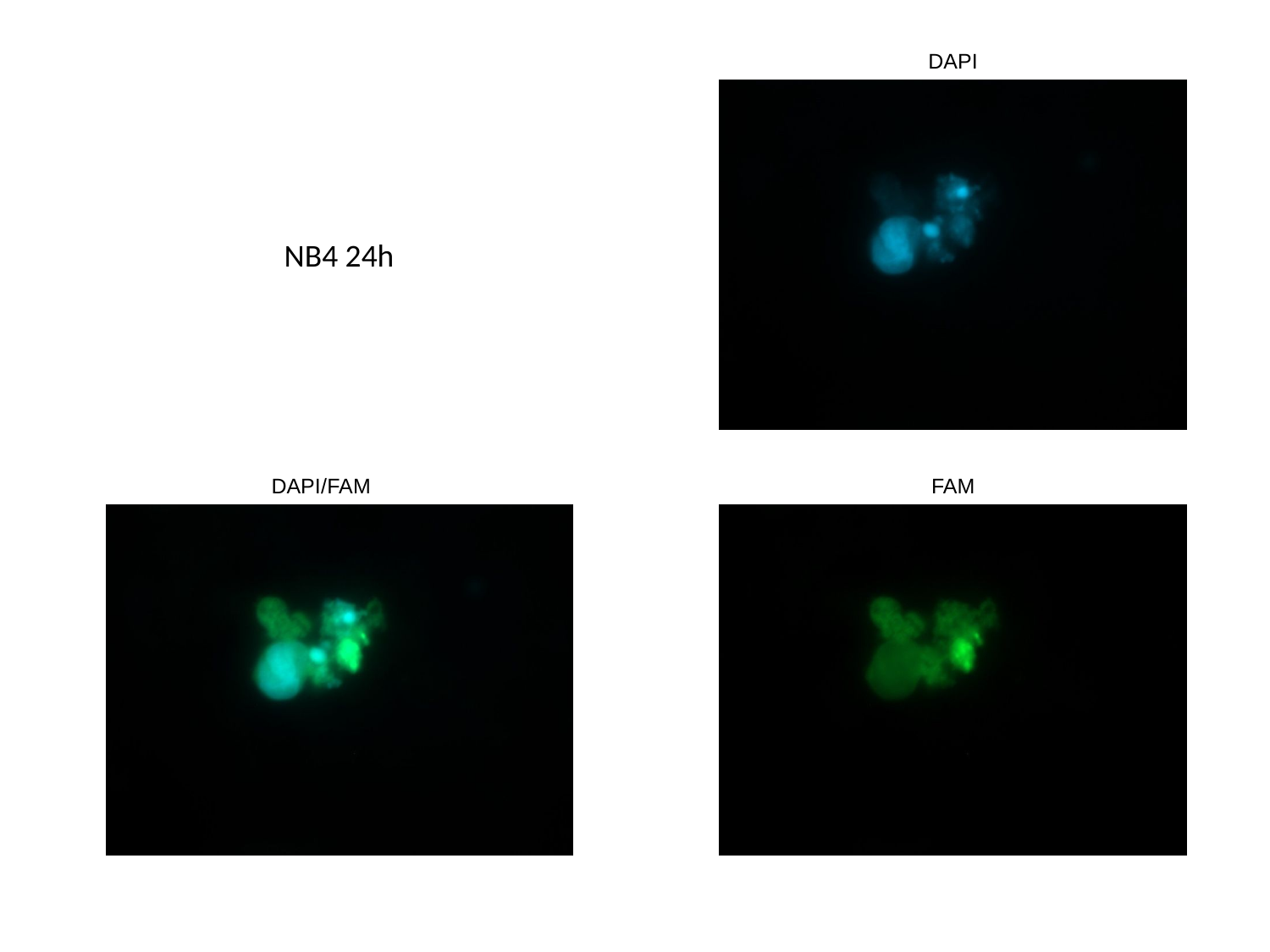

DAPI
NB4 24h
FAM
DAPI/FAM

## Slide 26
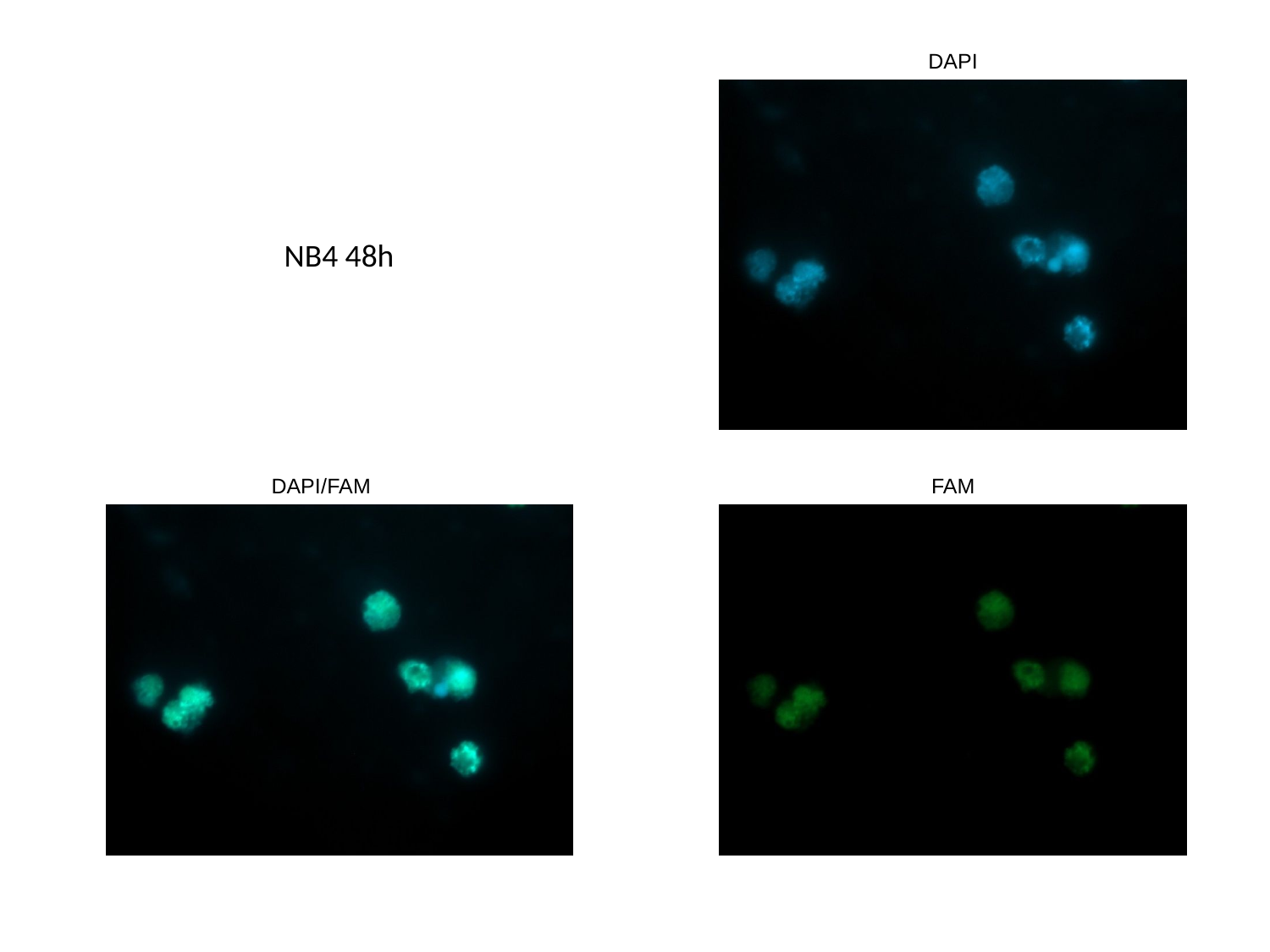

DAPI
NB4 48h
FAM
DAPI/FAM

## Slide 27
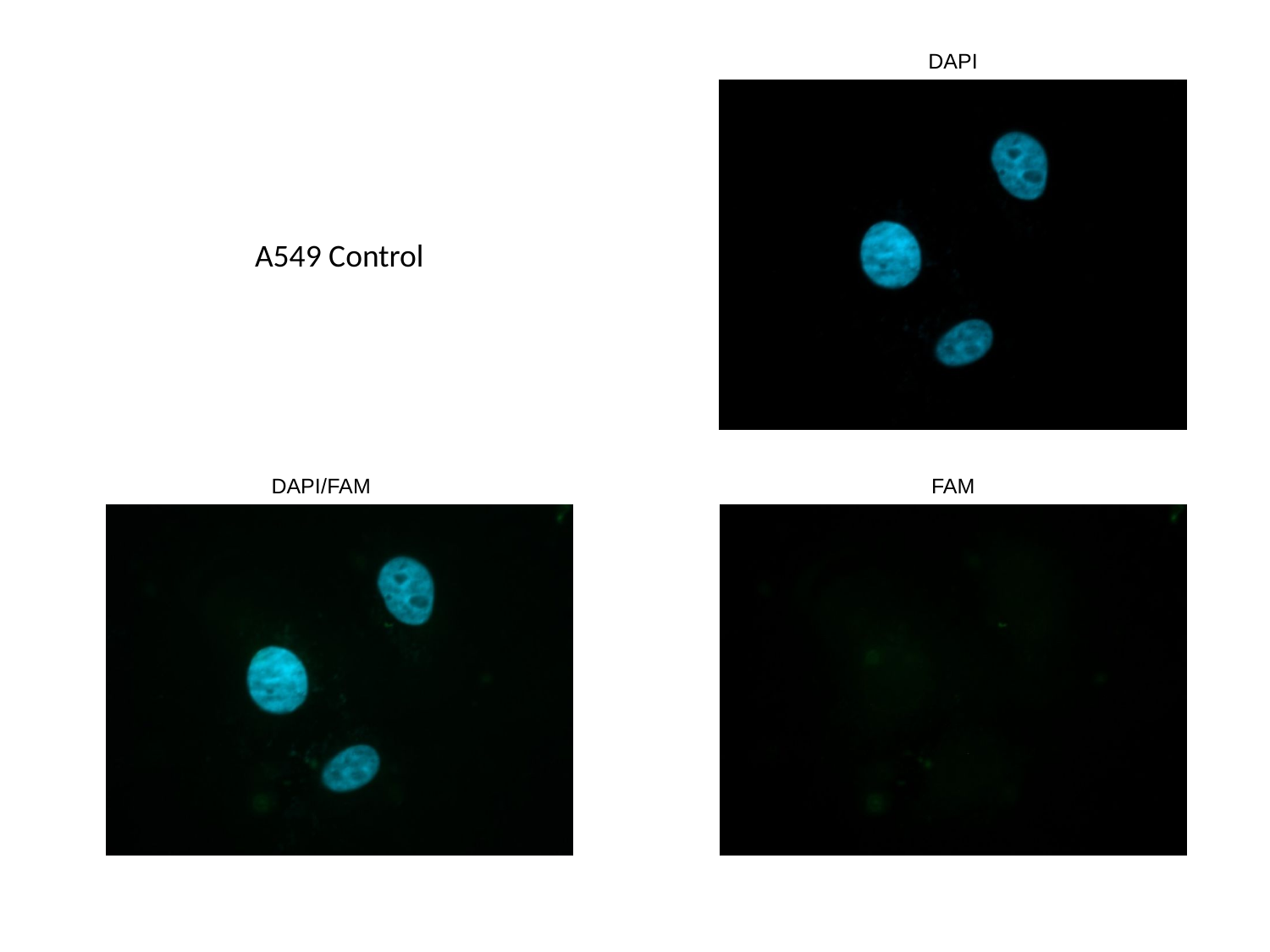

DAPI
A549 Control
FAM
DAPI/FAM

## Slide 28
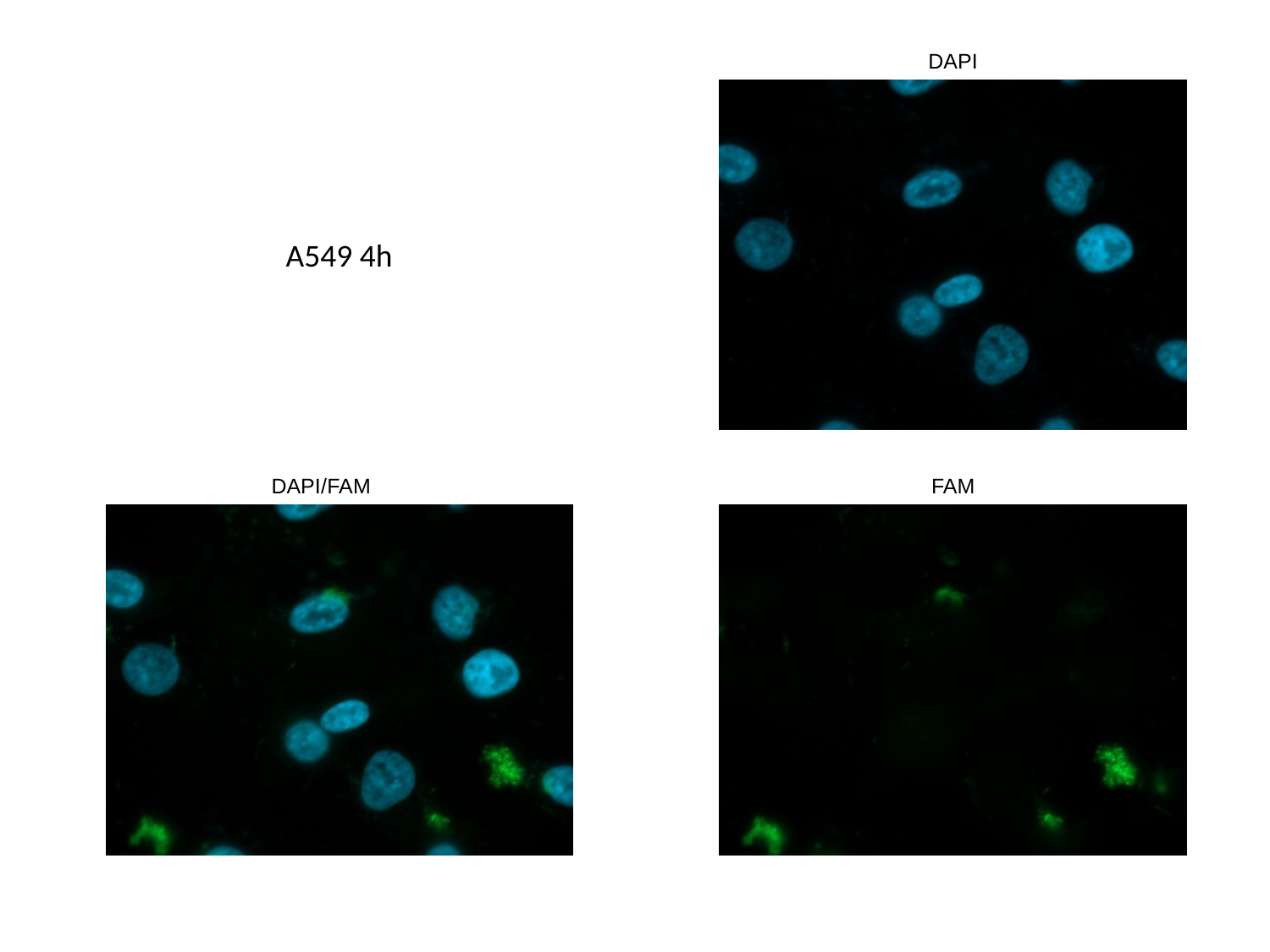

DAPI
A549 4h
FAM
DAPI/FAM

## Slide 29
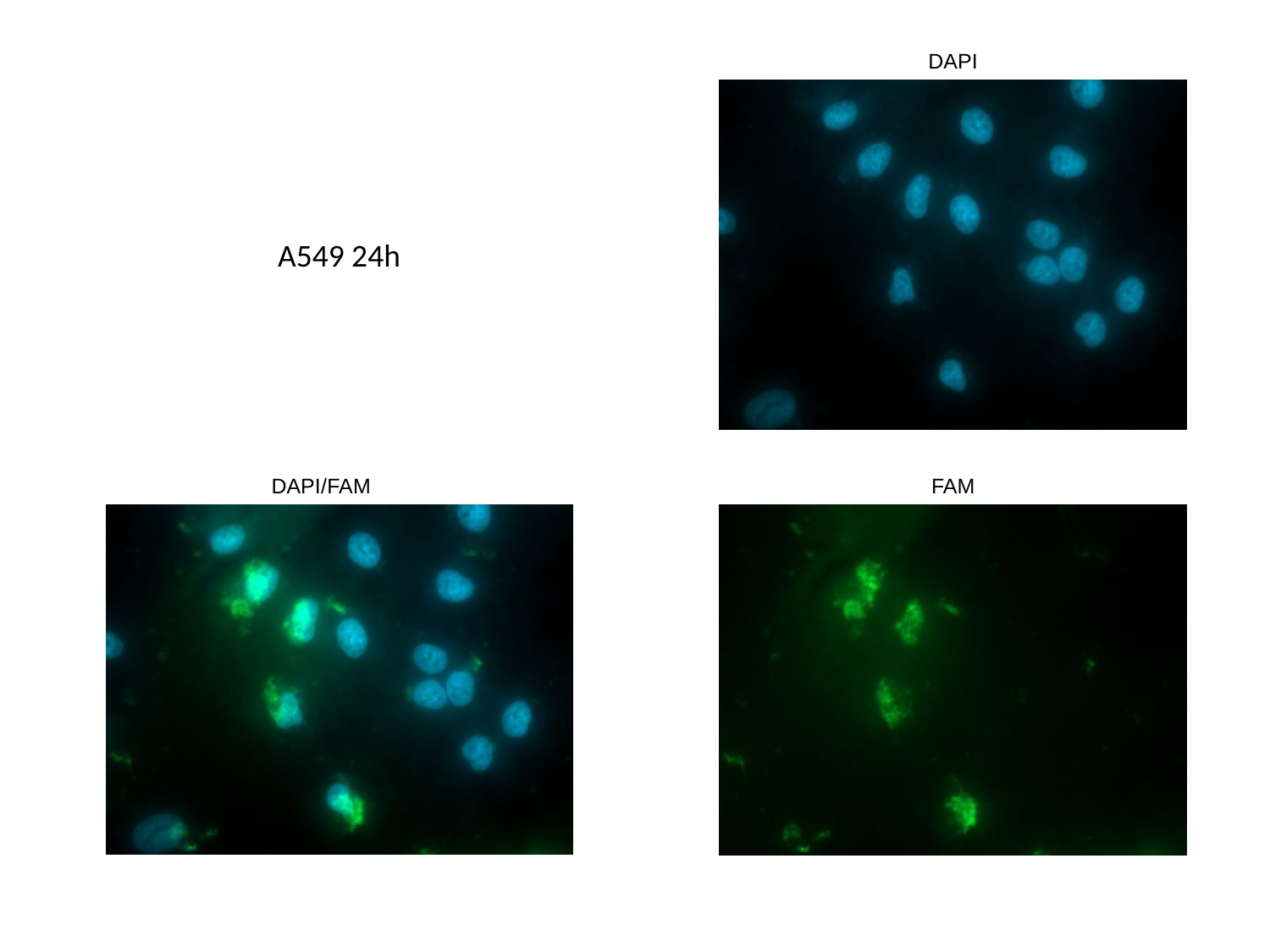

DAPI
A549 24h
FAM
DAPI/FAM

## Slide 30
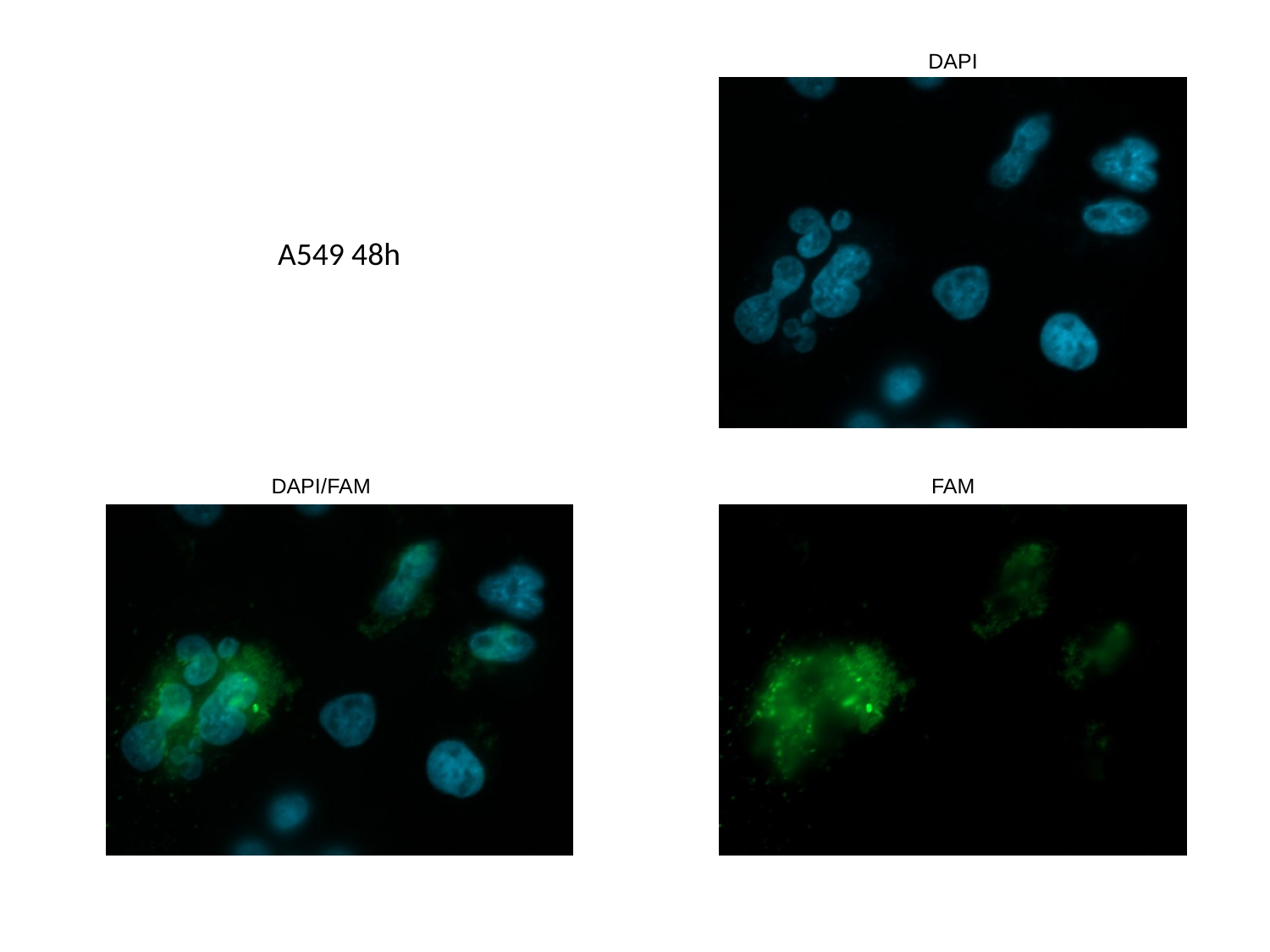

DAPI
A549 48h
FAM
DAPI/FAM

## Slide 31
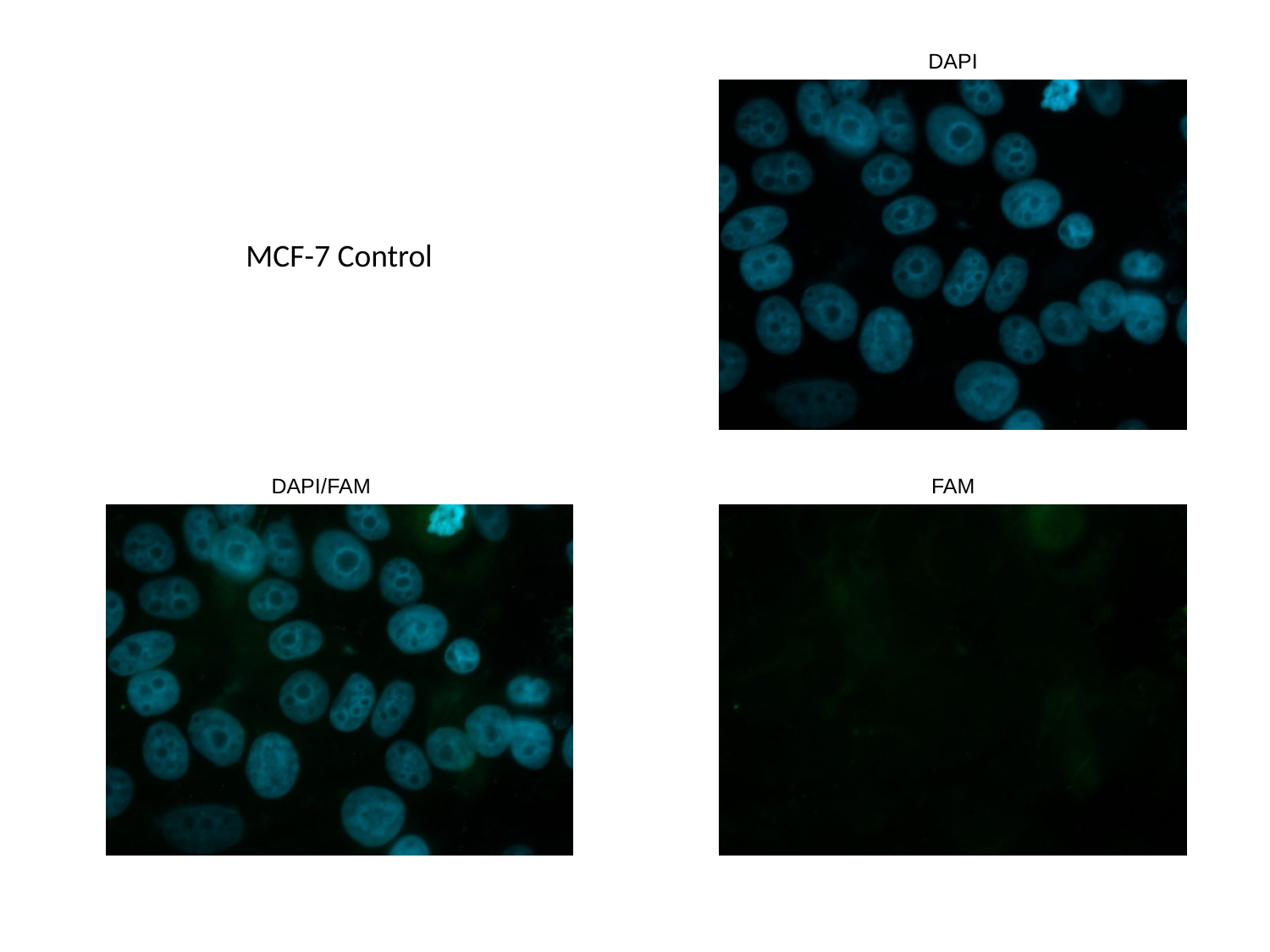

DAPI
MCF-7 Control
FAM
DAPI/FAM

## Slide 32
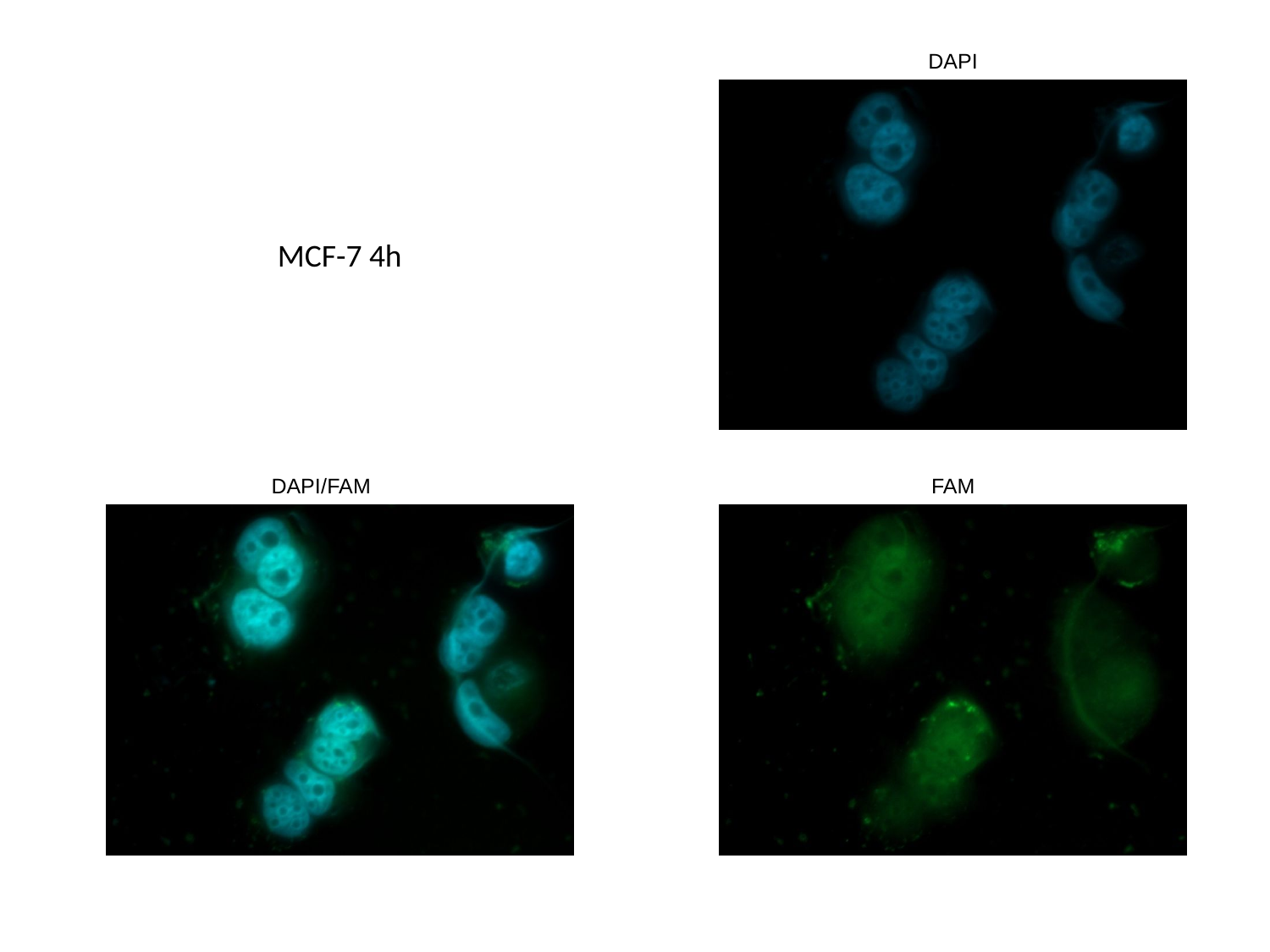

DAPI
MCF-7 4h
FAM
DAPI/FAM

## Slide 33
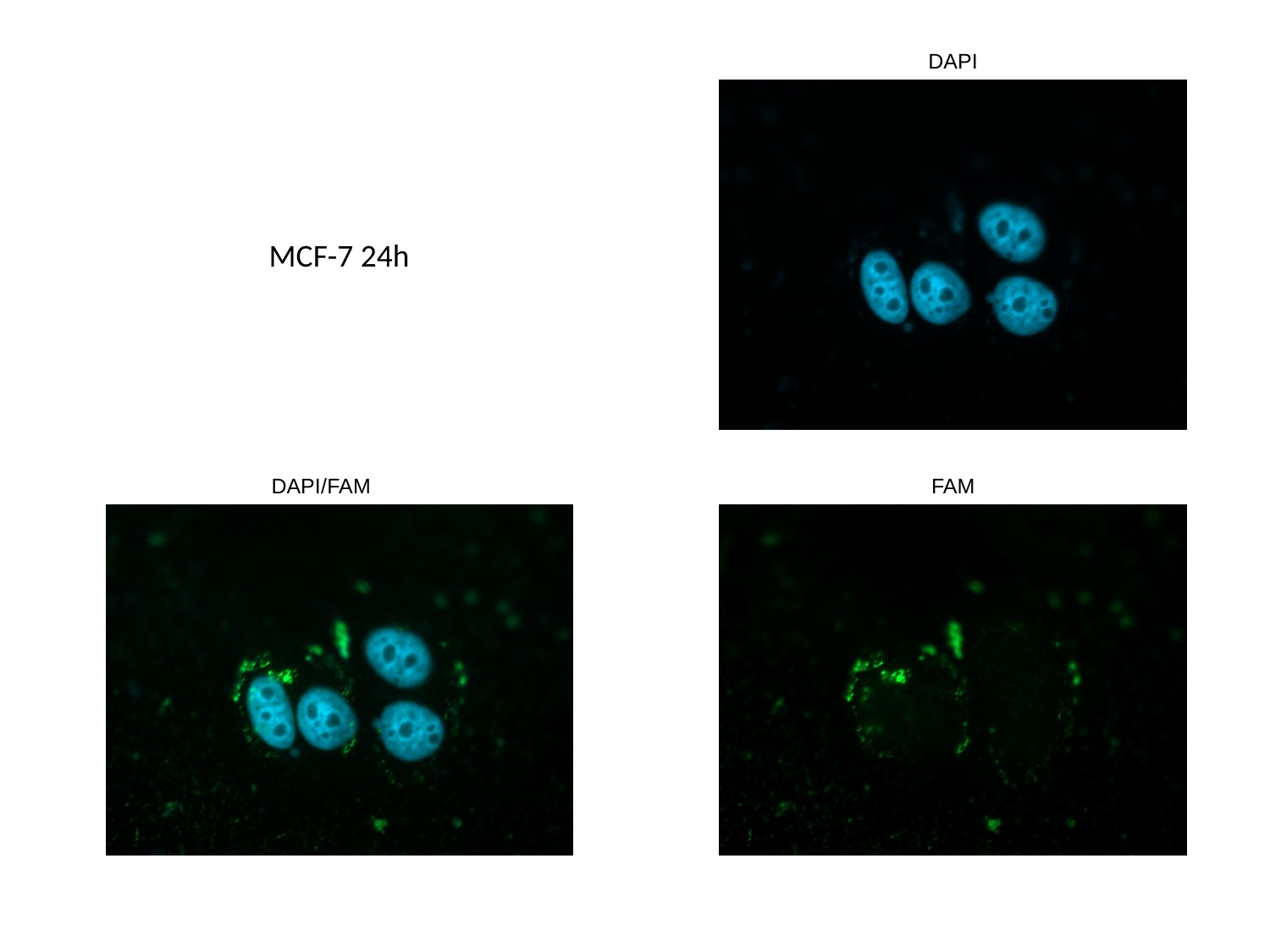

DAPI
MCF-7 24h
FAM
DAPI/FAM

## Slide 34
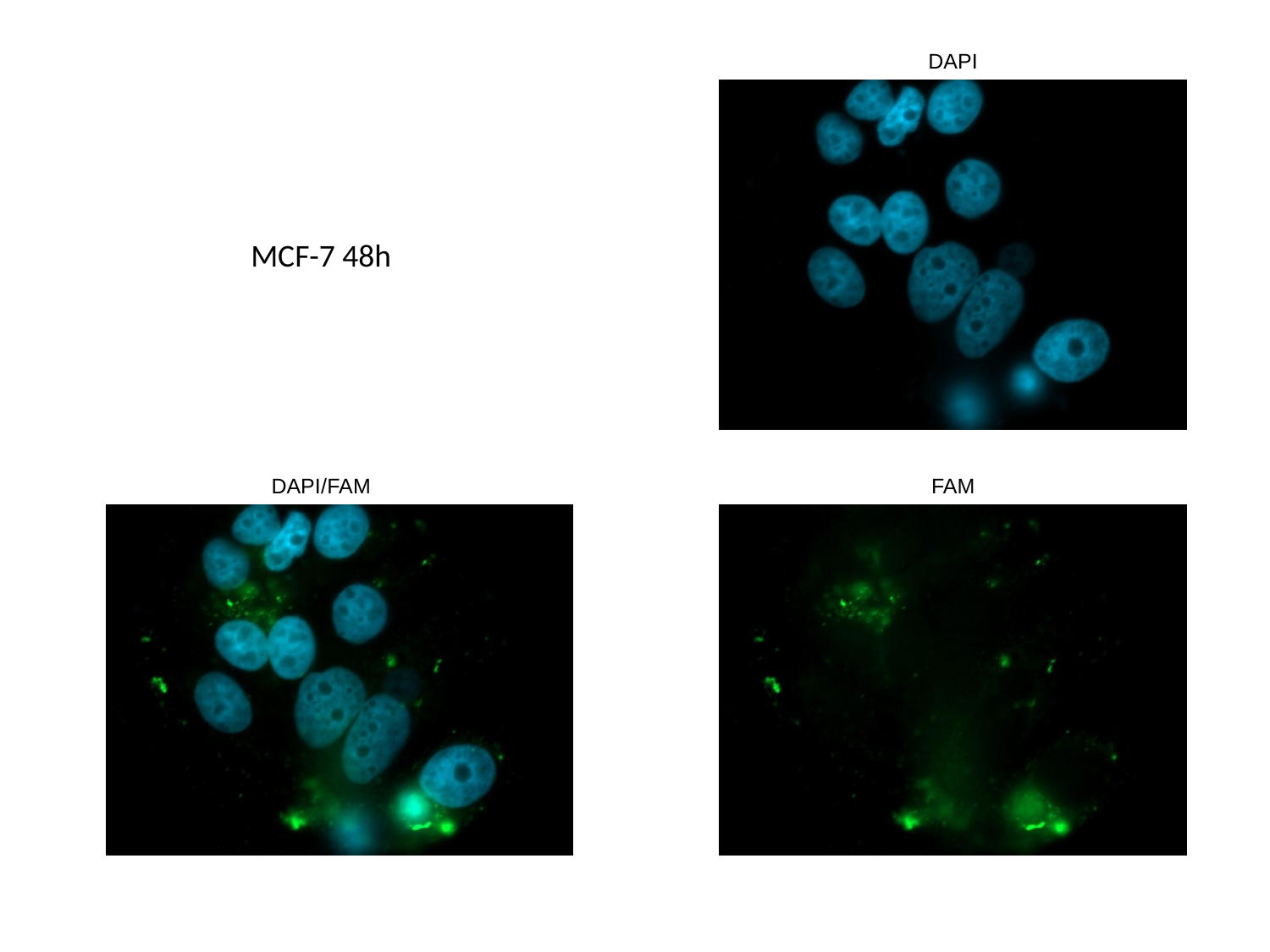

DAPI
MCF-7 48h
FAM
DAPI/FAM
